# Identification of Crucial lncRNAs, miRNAs, mRNAs, and Potential Therapeutic Compounds for Polycystic Ovary Syndrome by Bioinformatics Analysis

**DOI:** 10.1155/2020/1817094

**Published:** 2020-11-06

**Authors:** Zhi Zeng, Xia Lin, Tingting Xia, Wenxiu Liu, Xiaohui Tian, Manchao Li

**Affiliations:** ^1^Center of Reproductive Medicine, The Sixth Affiliated Hospital, Sun Yat-sen University, Guangzhou 510655, China; ^2^Department of Gynecology, The Third Affiliated Hospital of Sun Yat-sen University, Guangzhou 510630, China; ^3^Center for Reproductive Medicine, The Third Affiliated Hospital of Sun Yat-sen University, Guangzhou 510630, China; ^4^Department of Gynecology, The First Affiliated Hospital of Guangzhou University of Chinese Medicine, Guangzhou 510407, China; ^5^Department of Obstetrics and Gynecology, The Seventh Affiliated Hospital of Sun Yat-sen University, Shenzhen 518107, China

## Abstract

**Background:**

This study was aimed at mining crucial long noncoding RNAs (lncRNAs), microRNAs (miRNAs), and messenger RNAs (mRNAs) for the development of polycystic ovary syndrome (PCOS) based on the coexpression and the competitive endogenous RNA (ceRNA) theories and investigating the underlying therapeutic drugs that may function by reversing the expression of lncRNAs, miRNAs, and mRNAs.

**Methods:**

RNA (GSE106724, GSE114419, GSE137684, and GSE138518) or miRNA (GSE84376 and GSE138572) expression profile datasets of PCOS patients were downloaded from the Gene Expression Omnibus database. The weighted gene coexpression network analysis (WGCNA) using four RNA datasets was conducted to construct the lncRNA-mRNA coexpression networks, while the common differentially expressed miRNAs in two miRNA datasets and module RNAs were used to establish the ceRNA network. A protein-protein interaction (PPI) network was created to explore the potential interactions between genes. Gene Ontology and KEGG pathway enrichment analyses were performed to explore the functions of genes in networks. Connectivity Map (CMap) and Comparative Toxicogenomics Database (CTD) analyses were performed to identify potential therapeutic agents for PCOS.

**Results:**

Three modules (black, magenta, and yellow) were identified to be PCOS-related after WGCNA analysis, in which KLF3-AS1-PLCG2, MAPKAPK5-AS1-MAP3K14, and WWC2-AS2-TXNIP were important coexpression relationship pairs. WWC2-AS2-hsa-miR-382-PLCG2 was a crucial ceRNA loop in the ceRNA network. The PPI network showed that MAP3K14 and TXNIP could interact with hub genes PLK1 (degree = 21) and TLR1 (degree = 18), respectively. These genes were enriched into mitosis (PLK1), immune response (PLCG2 and TLR1), and cell cycle (TXNIP and PLK1) biological processes. Ten small molecule drugs (especially quercetin) were considered to be therapeutical for PCOS.

**Conclusion:**

Our study may provide a novel insight into the mechanisms and therapy for PCOS.

## 1. Introduction

Polycystic ovary syndrome (PCOS), characterized by polycystic ovary morphology, hyperandrogenism, and ovulatory disturbance, is the most frequent endocrine disorder in women of the reproductive age, affecting approximately 6%-9% of women worldwide [1]. PCOS is not only a leading cause of female infertility [2] but also an important risk factor for insulin resistance, diabetes, obesity, hypertension, metabolic syndrome, cardiovascular disease, and endometrial cancer [3]. However, the treatment of PCOS remains a challenge; thus, it is of significance to investigate the mechanisms and develop more effective preventative and therapeutic strategies.

Although the pathogenesis of PCOS has not been fully understood, apoptosis of ovarian granulosa cells (OGCs) is considered to be a potential contributor [4, 5]. By binding to the receptor on the OGCs, a follicle-stimulating hormone induces the expression of cholesterol side-chain cleavage cytochrome P450 (CYP19A1) and 17*β*-hydroxysteroid dehydrogenase (CYP17A1) which are rate-limiting enzymes to catalyze conversion from estrogen to estradiol [6]. Apoptosis of OGCs leads to the deficiency of estradiol and blocks the follicle development and maturation, ultimately forming more numbers of hypogenetic secondary follicles undergoing atresia and antral follicles [7]. Therefore, exploration of molecular mechanisms associated with the apoptosis of OGCs may provide underlying targets for developing drugs.

Accumulating evidence has suggested that long noncoding RNAs (lncRNAs), microRNAs (miRNAs), and messenger RNA (mRNAs) are important regulators of cell physiological and pathological processes (such as proliferation and apoptosis): mRNAs that encode the proteins were directly functional; miRNAs, approximately 22 nucleotide-long noncoding RNAs, act by binding to complementary sequences in the 3′ untranslated region (UTR) of mRNAs and then to negatively regulate the expressions of these mRNAs [8]; lncRNAs, >200 bp in length, can interact with the miRNA as a competing endogenous RNA (ceRNA) to regulate the expression of target genes [9] or directly regulate the transcription of mRNAs via a coexpression manner [10], indicating that aberrant expression of lncRNAs, miRNAs, and mRNAs in OGCs may be the primary contributors for PCOS development and potential targets for the treatment of PCOS. This theory has been demonstrated in some studies. For example, Yang et al. found that the expression level of lncRNA BANCR was significantly higher in OGCs of patients with PCOS compared with non-PCOS patients. Transfection with BANCR expression vector significantly inhibited proliferation, promoted apoptosis of ovarian granulosa-like tumor cell line KGN, and significantly enhanced the expression of proapoptotic Bax and p53 [5]. Li et al. reported that lncRNA SRLR was upregulated in PCOS patients compared with heathy females. Overexpression of lncRNA SRLR promoted apoptosis of KGN cells by upregulation of interleukin-6 (IL-6) [11]. The study of Fu et al. revealed that miR-16 expression was downregulated in ovarian cortex tissues of PCOS patients. Overexpression of miR-16 inhibited apoptosis of OGC *in vitro* and alleviated PCOS *in vivo*, which was associated with its function to downregulate its direct target programmed cell death 4 (PDCD4). Enforced expression of PDCD4 reversed the effects of miR-16 on OGC apoptosis [12]. Liu et al. demonstrated that lncRNA PVT1 may regulate the progression of PCOS by modulating miR-17-5p-PTEN: overexpressed PVT1 could bind with and inhibit the expression of miR-17-5p in OGCs of PCOS, thereby preventing the inhibitory effects of miR-17-5p for its target gene PTEN and leading to the elevated expression of PTEN. shRNA-mediated silencing of PVT1 and transfection with miR-17-5p mimics could decelerate apoptosis, while accelerate colony formation ability and proliferation of OGCs [13]. Wang et al. observed that cotreatment with metformin and sitagliptin attenuated the apoptosis in PCOS model cells by inducing lncRNA H19 expression and proposed the three-combined strategy (metformin-sitagliptin-H19-expressing lentiviruses) for the treatment of PCOS [14]. However, crucial lncRNAs, miRNAs, mRNAs, and drugs that target these molecules remain rarely reported for PCOS.

Recently, with the development of the sequencing technique, there were some studies to mine the key lncRNAs [15], miRNAs [16–18], and mRNAs [15, 17, 19] in OGCs of PCOS patients using the gene or miRNA expression profile data. However, all of them focused only on the differentially expressed mRNAs (coding genes) (DEGs) [15, 19], lncRNAs (DELs) [15], miRNAs (DEMs) alone [16, 18], or DEM-DEG [17]. Also, the small molecule drugs that targeted them were not investigated. In this study, we aimed to screen pivotal lncRNAs, miRNAs, and mRNAs based on lncRNA-mRNA coexpression and lncRNA-miRNA-mRNA ceRNA mechanisms via integrated analysis of multiple high-throughput datasets. In addition, the Connectivity Map (CMap) and Comparative Toxicogenomics Database (CTD) analyses were performed to acquire bioactive compounds that may have potential for the treatment of PCOS by regulating the expression of DEGs, DELs, and DEMs.

## 2. Materials and Methods

### 2.1. Microarray Data

Multiple high-throughput datasets of PCOS were downloaded from the Gene Expression Omnibus (GEO, http://www. http://ncbi.nlm.nih.gov/geo/) database on February 25, 2020: (a) mRNA+lncRNA expression profile, including (1) GSE106724: it contained 12 samples of ovarian granulosa cells from normal control (n = 4) and PCOS (n = 8). Its analysis platform was Agilent-062918 Human lncRNA array V4.0 (Probe Name version). (2) GSE114419: 6 subjects [normal control (*n* = 3) and PCOS (*n* = 3)] were enrolled for obtaining their ovarian granulosa cells. Its analysis platform was Affymetrix Human Transcriptome Array 2.0 [transcript (gene) version] [20]. (3) GSE137684: ovarian granulosa cells of 4 normal control and 8 PCOS patients were available. Its analysis platform was Agilent-039494 SurePrint G3 Human GE v2 8x60K Microarray 039381 (Probe Name version). (4) GSE138518: RNA sequencing in ovarian granulosa cells of PCOS patients (*n* = 3) and normal people (*n* = 3), which were run on an Illumina HiSeq 2000 platform. (b) miRNA expression profile, including (1) GSE138572: small RNA sequencing by an Illumina HiSeq 2000 platform was applied in ovarian granulosa cells of PCOS patients (*n* = 5) and normal people (*n* = 5) and (2) GSE84376: granulosa cells of 15 PCOS patients and 13 control individuals were obtained. Its miRNA analysis was carried out using the Affymetrix GeneChip miRNA 3.0 array.

### 2.2. Differential Expression Analysis

The mRNAs and lncRNAs in the above four datasets were reannotated according to the official nomenclature in the HUGO Gene Nomenclature Committee (HGNC; http://www.genenames.org/) [21]. Only the shared mRNAs and lncRNAs in all of the included datasets were obtained for the differential analysis. The DELs and DEGs between PCOS and normal controls were identified using the MetaDE.ES function in the MetaDE package (version 1.0.5, https://cran.r-project.org/web/packages/MetaDE/). The threshold was set based on three aspects: (1) no heterogeneity was present among these datasets, which was assessed by tau^2^ statistic (=0) and chi-square-based *Q*-test (*p* > 0.05); (2) false discovery rate (FDR) was less than 0.05; and (3) the differential trend was consistent based on log_2_FC (fold change).

The Linear Models for Microarray Data (LIMMA) package (version 3.34.7; https://bioconductor.org/packages/release/bioc/html/limma.html) [22] for R was used for identification of DEMs in GSE138572 and GSE84376 datasets, with ∣logFC | >0.263 and false discovery rate (FDR) < 0.05 defined as the cut-off point. The DEMs commonly screened in two datasets were shown by Venn diagram. The volcano plot was visualized using the ggplot2 package (version 3.3.0; https://cran.r-project.org/web/packages/ggplot2) in R. A heat map was created using the “pheatmap” package (version: 1.0.8; https://cran.r-project.org/web/packages/pheatmap) in R.

### 2.3. Crucial lncRNA and mRNA Identification for PCOS

Weighted gene coexpression network analysis (WGCNA) package in R (version 1.61; https://cran.r-project.org/web/packages/WGCNA/index.html) [23] was used to cluster mRNAs and lncRNAs into several coexpression modules in which genes were highly correlated and considered to be crucial. During this WGCNA analysis, four steps were included: (1) calculating the expression and connectivity correlations of lncRNAs and mRNAs between any two datasets (GSE106724, GSE114419, GSE137684, and GSE138518), with correlationcoefficient > 0.6 and *p* value > 0.001 defined as significantly correlated; (2) using the GSE106724 dataset to select the soft threshold power (*β*) based on the scale-free topology criterion (that is, when the *R*^2^ reached 0.9 for the first time); (3) generating the dendrogram by calculation of the topological overlap matrix dissimilarity between genes in the GSE106724 dataset and identifying gene modules (cutHeight = 0.99 and minSize ≥ 100) by the dynamicTreeCut method [24]; (4) assessing the preservation (*Z*‐score > 5 and *p* < 0.05) of the identified modules in the GSE114419, GSE137684, and GSE138518 datasets using the modulePreservation statistics [25]; (5) mapping the DEGs and DELs into the modules and screening the modules with more DEGs and DELs (*p* < 0.05, foldenrichment > 1) by using the hypergeometric algorithm [f(*k*, *N*, *M*, *n*) = *C*(*k*, *M*)∗*C*(*n* − *k*, *N* − *M*)/*C*(*n*, *N*)] [26]; and (6) correlating coexpression gene modules with PCOS development by moduleTraitCor and moduleTraitPvalue algorithms.

### 2.4. lncRNA-mRNA Coexpression Network Visualization

The associations between the DEGs and DELs screened in the crucial WGCNA modules were evaluated by using the cor.test function (https://stat.ethz.ch/R-manual/R-devel/library/stats/html/cor.test.html) in R, and then the coexpression network was constructed based on their Pearson correlation coefficients (PCC). The coexpression network was visualized using Cytoscape (version 3.6.1; http://www.cytoscape.org/).

### 2.5. lncRNA-miRNA-mRNA ceRNA Network Construction

Interaction relationships of module DELs and DEGs with miRNAs were, respectively, predicted with the DIANA-LncBase (v2.0; http://carolina.imis.athena-innovation.gr/diana_tools/web/index.php?r=lncbasev2/index-predicted) [27] and starBase (v2.0; http://starbase.sysu.edu.cn/) [28] databases. lncRNA-miRNA-mRNA interaction axes were selected according to the overlapped miRNAs interacted with DEGs and DELs. The Cytoscape (version 3.6.1; http://www.cytoscape.org/) software was used for visualization of the ceRNA regulatory network.

### 2.6. Protein-Protein Interaction (PPI) Network Construction

The interactions between proteins coding by the module DEGs were predicted using the STRING (Search Tool for the Retrieval of Interacting Genes; version 10.0; https://string-db.org) database [29]. PPIs with a confidencescore ≥ 0.4 were chosen to construct the network. The PPI network was visualized by Cytoscape software (version 3.6.1; http://www.cytoscape.org/). Furthermore, the CytoNCA plugin in Cytoscape software (http://apps.cytoscape.org/apps/cytonca) [30] was applied to explore the hub genes by calculating the topological features of each protein in the PPI network, including degree centrality (DC), betweenness centrality (BC), and closeness centrality (CC). The proteins with high DC, BC, and CC were considered as hubs and essential for PCOS.

### 2.7. Function Enrichment Analysis

The Gene Ontology (GO) annotation, Kyoto Encyclopedia of Genes and Genomes (KEGG), and REACTOME pathway analyses were conducted for the DEGs in the PPI network using the online Database for Annotation, Visualization and Integrated Discovery (DAVID) (version 6.8; http://david.abcc.ncifcrf.gov) [31]. *p* value < 0.05 under hypergeometric test was considered as statistically significant.

### 2.8. Small Molecule Drug Analysis

The CMap (https://portals.broadinstitute.org/cmap/) was used to identify candidate small molecule drugs that may have potential to treat PCOS patients. The identified module DEGs were uploaded into the CMap web tool, and then the resultant small molecules with negative connectivity scores were considered to be therapeutic agents because it could reverse the expression of the query signature. The small molecules with the enrichment score approximate to -1 may be especially effective. Furthermore, CTD (http://ctdbase.org) was also searched using the gene name, after which a serial of chemical-gene interaction pairs were obtained. The small molecules identified by CMap and CTD were then compared with these chemical-gene interaction pairs to screen the common. The gene-drug interaction networks were visualized using Cytoscape (version 3.6.1; http://www.cytoscape.org/).

## 3. Results

### 3.1. Identification of DELs, DEGs, and DEMs

**Figure 1:**
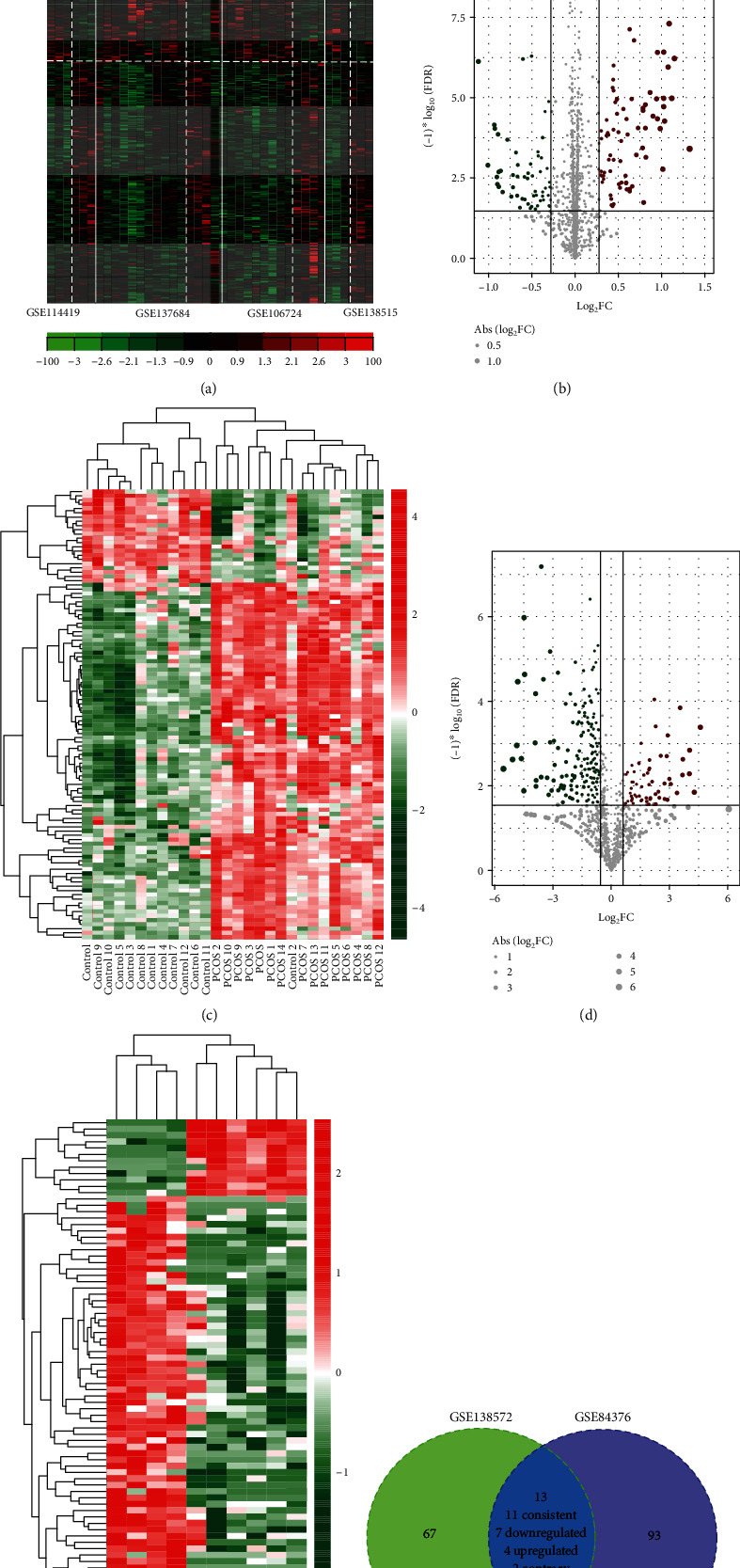
Differentially expressed RNAs and miRNAs. (a) The heat map of differentially expressed RNA (including mRNAs and lncRNAs) identified in four datasets (GSE106724, GSE114419, GSE137684, and GSE138518); (b, d) the volcano plot of differentially expressed miRNAs identified in the GSE84376 (b) and GSE138572 (d) datasets, respectively; (c, e) the heat map of differentially expressed miRNAs identified in the GSE84376 (c) and GSE138572 (e) datasets, respectively; (f) Venn diagram to identify the common differentially expressed miRNAs between GSE84376 and GSE138572 datasets.

A total of 70 lncRNAs and 10,481 protein-encoding mRNAs were annotated in all GSE106724, GSE114419, GSE137684, and GSE138518 datasets, which then underwent MetaDE analysis to identify DELs and DEGs. As a result, 754 differentially expressed RNAs were found between PCOS patients and normal controls, including 19 DELs (upregulated, 18; downregulated, 1) and 735 DEGs (upregulated, 551; downregulated, 184) (Table S1). Heat map analysis showed that these DELs and DEGs can obviously cluster the samples into two groups in any one dataset (Figure 1(a)).

Furthermore, LIMMA analysis was used to screen DEMs in GSE138572 and GSE84376, respectively. The results showed that 80 DEMs (upregulated, 16; downregulated, 64) were identified for the GSE138572 dataset (Figure 1(b)) (Table S2), while 106 (upregulated, 74; downregulated, 32) for the GSE84376 dataset (Figure 1(d)) (Table S3). These DEMs also can obviously distinguish the PCOS patients from normal controls in the GSE138572 (Figure 1(c)) and GSE84376 (Figure 1(e)) datasets. The Venn diagram revealed that there were 11 DEMs (including 4 upregulated and 7 downregulated) consistently expressed in these two datasets (Figure 1(f)).

### 3.2. Construction of lncRNA-mRNA Coexpression Network Using Module Genes

**Figure 2:**
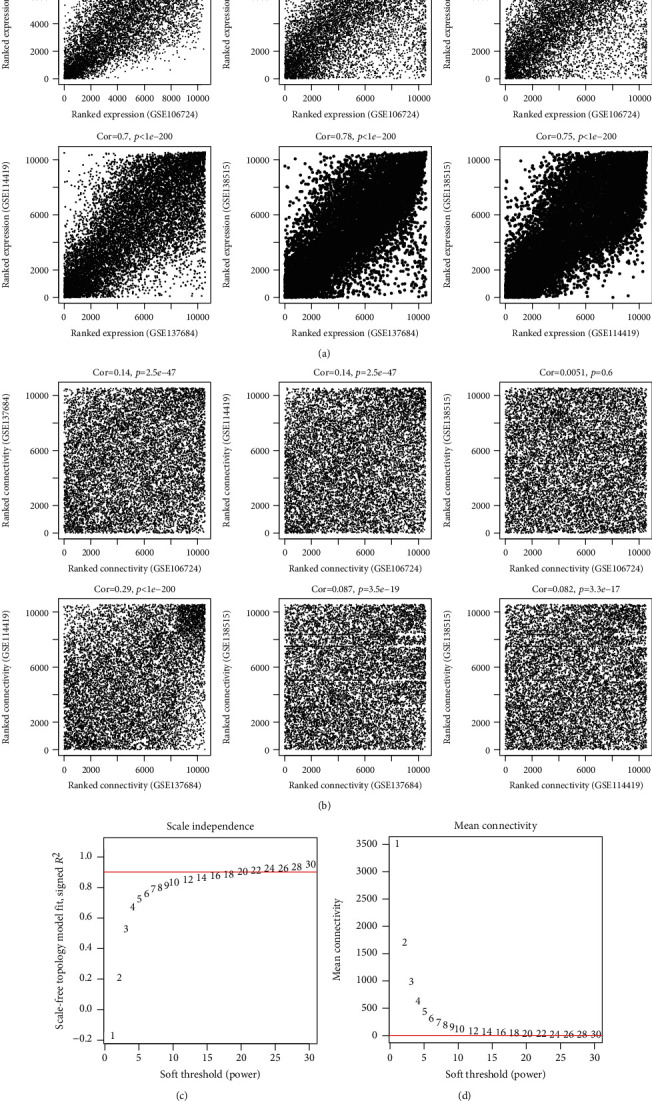
Assessment of the correlation between datasets and selection of soft threshold power *β* based on the training dataset. (a) The correlation of the RNA expression levels; (b) the correlation of the connectivity; (c) selection of power when the square value was equal to the red standard line (0.9) for the first time; (d) calculation of mean connectivity according to *β* values.

**Figure 3:**
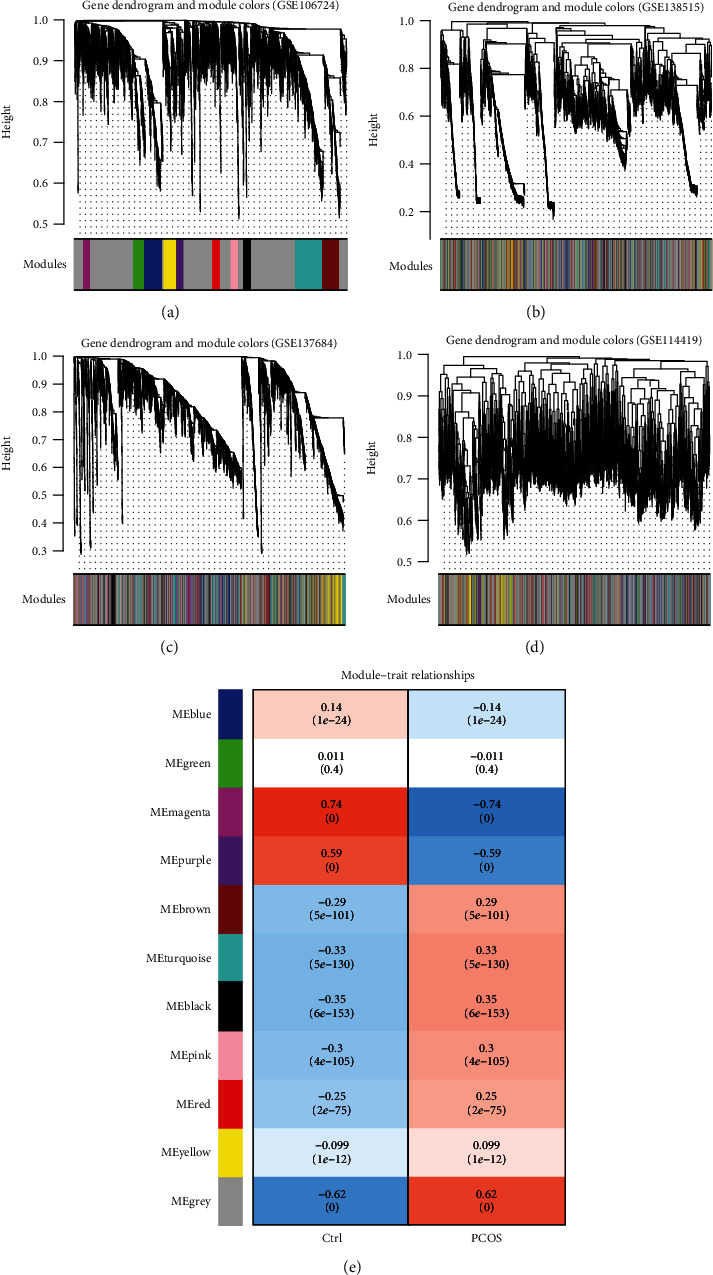
WGCNA analysis. (a–d) Clustering of differentially expressed lncRNAs and mRNAs of GSE106724 (a), GSE138518 (b), GSE137684 (c), and GSE114419 (d) datasets; (e) the correlation between gene modules and PCOS development.

**Figure 4:**
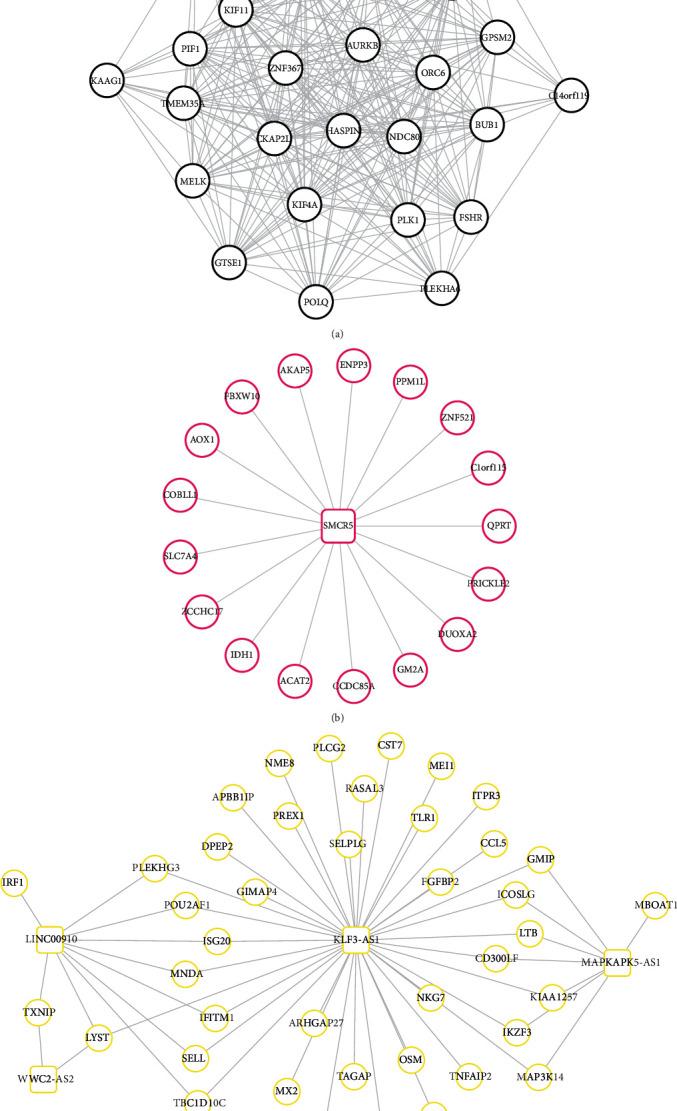
The coexpression relationships in three crucial modules: (a) black module; (b) magenta module; (c) yellow module. Genes in boxes indicate the lncRNAs; genes in circles indicate the mRNAs.

Based on GSE106724, GSE114419, GSE137684, and GSE138518 datasets, WGCNA was applied to detect the PCOS-related modules which included the potential interactions between lncRNAs and mRNAs. These four datasets were considered to be comparable because there were significantly positive correlations of RNAs (coefficient > 0.6 and *p* value < 1*e*-200) between any two datasets irrespective of their expression levels (Figure 2(a)) or the connectivity (Figure 2(b)). As shown in Figures 2(c) and 2(d), power = 20 was chosen as the soft-thresholding to ensure a scale-free network (*R*^2^ = 0.9; meanconnectivity = 1). A cluster dendrogram showed that eleven colors of modules (black, blue, brown, green, grey, magenta, pink, purple, red, turquoise, and yellow, ranging in size from 136 to 2,812) were identified using the GSE106724 dataset (Figure 3(a); Table 1). These modules were also observed in the analysis of GSE138518, GSE137684, and GSE114419 datasets (Figures 3(b)–3(d)). Among these eleven modules, black, blue, magenta, purple, turquoise, and yellow may be preserved because of their *Z*‐score > 5 and *p* value < 0.05 (Table 1). Black, magenta, and yellow modules were further considered to be crucial for PCOS because there were relatively many DEGs or DELs enriched in them (enrichmentfold > 1 and *p* value < 0.05) (Table 1). This conclusion was also demonstrated according to the results of a correlation heat map in which black (*p* = 1*e*‐24), magenta (*p* = 0), and yellow (*p* = 1*e*‐12) modules were significantly, positively related with the development of PCOS (Figure 3(e)). Subsequently, the expressions of DELs and DEGs in these three modules were extracted and the PCC were calculated to construct the coexpression network. As shown in Figure 4, all the included genes in the black module were DEGs (26) (Figure 4(a)), while the magenta module (Figure 4(b)) had 1 DEL (SMCR5) and 20 DEGs; the yellow module (Figure 4(c)) had 4 DELs (KLF3-AS1, LINC00910, MAPKAPK5-AS1, and WWC2-AS2) and 45 DEGs, indicating the 160 interaction pairs of the magenta and yellow modules may be especially pivotal [such as LINC00910-thioredoxin interacting protein (TXNIP), KLF3-AS1-phospholipase C gamma 2 (PLCG2), MAPKAPK5-AS1-mitogen-activated protein kinase kinase kinase 14 (MAP3K14), and WWC2-AS2-TXNIP] for PCOS.

### 3.3. Construction of the ceRNA Regulatory Network

**Figure 5:**
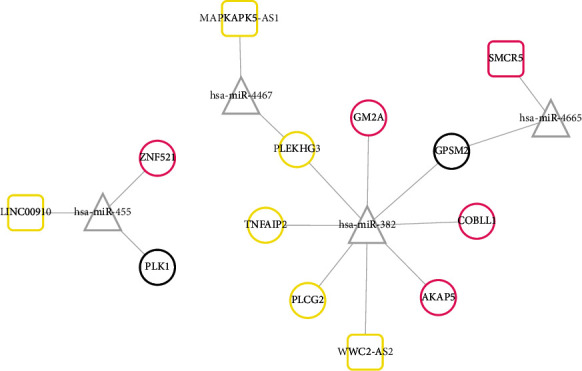
Construction of the ceRNA network using the lncRNAs, mRNAs in three crucial modules, and common miRNAs identified by GSE84376 and GSE138572 datasets. Genes in boxes indicate the lncRNAs; genes in circles indicate the mRNAs; genes in triangles indicate the miRNAs. Different colors for lncRNAs and mRNAs represent different modules.

The ceRNA regulatory network was also constructed using the DELs and DEGs in the black, magenta, and yellow modules. A total of 4 interaction relationship pairs between 4 DELs (LINC00910, MAPKAPK5-AS1, SMCR5, and WWC2-AS2) and 4 DEMs (hsa-miR-455, hsa-miR-4467, hsa-miR-4665, and hsa-miR-382) were predicted by the DIANA-LncBase database, while 12 paired interactions were predicted between 4 DEMs and 9 DEGs by the starBase database. Then, the lncRNA-miRNA-mRNA network was established based on these ceRNA loops (Figure 5), including SMCR5-hsa-miR-4665-G protein signaling modulator 2 (GPSM2), LINC00910-hsa-miR-455-polo like kinase 1 (PLK1), MAPKAPK5-AS1-hsa-miR-4467-PLEKHG3, and WWC2-AS2-hsa-miR-382-PLCG2. However, only WWC2-AS2-hsa-miR-382-PLCG2 may be believable because the expressions of WWC2-AS2 (upregulated) and PLCG2 (upregulated) were opposite to hsa-miR-382 (downregulated).

### 3.4. Construction of the PPI Network

**Figure 6:**
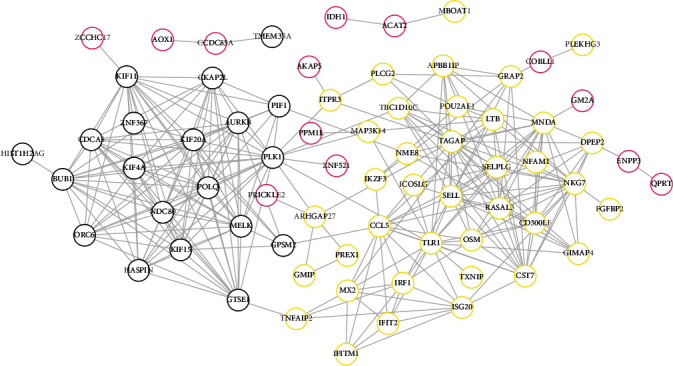
Construction of the PPI network for all the differentially expressed mRNAs in three crucial modules. Different colors for mRNAs represent different modules.

In order to provide the potential interpretations for the DEGs that may have no function enrichment, a PPI network was constructed for the DEGs in crucial modules. As a result, a total of 280 interaction pairs were identified, such as MAP3K14-PLK1 and TXNIP-toll-like receptor 1 (TLR1) (Figure 6). PLK1 and TLR1 were considered as hub genes in the PPI because they belonged to the top 20 genes ranked by DC, BC, and CC (Table 2).

### 3.5. Function Enrichment Analysis

A total of 18 GO biological process terms were enriched for the DEGs in the PPI network (Table 3), such as GO:0000280 ~ nuclear division (PLK1), GO:0007067 ~ mitosis (PLK1), GO:0006955 ~ immune response (PLCG2 and TLR1), and GO:0007049 ~ cell cycle (TXNIP and PLK1). Two KEGG pathways were also obtained (Table 3), including hsa05120: epithelial cell signaling in Helicobacter pylori infection (PLCG2 and MAP3K14).

### 3.6. Identification of Small Molecule Drugs

**Figure 7:**
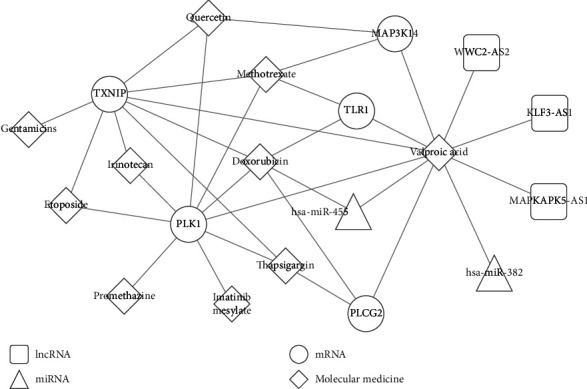
The target relationships between differentially expressed RNAs and small molecular drugs.

A total of 629 small molecules were found to have the negative enrichment score, indicating they may be potential therapeutical drugs for PCOS. Ten small molecule drugs enriched by CMap (irinotecan, score = −0.902; doxorubicin, score = −0.787; imatinib, score = −0.714; etoposide, score = −0.696; methotrexate, score = −0.6; promethazine, score = −0.563; gentamicin, score = −0.562; thapsigargin, score = −0.548; quercetin, score = −0.538; and valproic acid, score = −0.192) were identified to target crucial genes in CTD database analysis (Figure 7). Among them, valproic acid could target any genes in the ceRNA axes of WWC2-AS2-hsa-miR-382-PLCG2 and coexpression axes of KLF3-AS1-PLCG2, MAPKAPK5-AS1-MAP3K14, and WWC2-AS2-TXNIP. Furthermore, doxorubicin (PLK1, PLCG2, TXNIP, and TLR1) and methotrexate (PLK1, TXNIP, MAP3K14, and TLR1) targeted 4 mRNAs; quercetin (PLK1, TXNIP, and MAP3K14) and thapsigargin (PLK1, PLCG2, and TXNIP) targeted 3 mRNAs; etoposide and irinotecan targeted 2 mRNAs (PLK1 and TXNIP); and imatinib (PLK1), gentamicin (TXNIP), and promethazine (PLK1) targeted 1 mRNA.

## 4. Discussion

Although previous studies have attempted to reveal the molecular mechanisms of PCOS by using high-throughput microarray or sequencing data [15–19], all of them focused on the single dataset (mRNA: GSE95728 [15], miRNA: GSE84376 [16, 18], miRNA: GSE84376 and mRNA: GSE34526 [17], and mRNA: GSE34526 [19]) which may result in a high probability of false positive results. In order to prevent this shortcoming, in this study, we analyzed four lncRNA-mRNA expression profile datasets (GSE106724, GSE114419, GSE137684, and GSE138518) using the MetaDE package and only selected the DELs and DEGs that overlapped in the four datasets. The crucial DELs and DEGs were screened via WGCNA analysis which used GSE106724 as the training dataset and GSE114419, GSE137684, and GSE138518 as the validation datasets. Similarly, two miRNA expression profile datasets were used. Although some key miRNAs and mRNAs identified in the previous studies (miR-3135, miR-3188 [16, 18], and miR-486 [17]; aquaporin 9, free fatty acid receptor 2, and S100 calcium binding protein A8 [15]) were also found in our study, they were not the focus because they were only identified in one dataset (miRNA, GSE84376) or not included in preserved modules. More interestingly, we, for the first time, integrated the DEL-DEG and DEL-DEM-DEG data to construct the coexpression and ceRNA network. As expected, we identified three new coexpression relationships between 3 DELs and their target genes (KLF3-AS1-PLCG2, MAPKAPK5-AS1-MAP3K14, and WWC2-AS2-TXNIP) and one ceRNA axis among WWC2-AS2, hsa-miR-382, and PLCG2. These mRNAs were predicted to be involved in PCOS by influencing cell mitosis, cell cycle, and immune response. These three DELs (KLF3-AS1, MAPKAPK5-AS1, and WWC2-AS2), one DEM (hsa-miR-382), and five DEGs [including 3 in the coexpression or ceRNA axes (PLCG2, MAP3K14, and TXNIP) and 2 interacted genes (PLK and TLR1) in the PPI network] were predicted to be regulated by 10 small molecular drugs for the treatment of PCOS via CMap and CTD analyses.

Among all these DELs, DEMs, and DEGs, only TXNIP, TXNIP-interacted TLR1, and miR-382-5p were directly reported to be associated with PCOS. TXNIP expression was downregulated during the time of *in vitro* culture of the OGCs [32–34]. Upregulation of TXNIP in the OGCs resulted in the patients suffering PCOS [35]. Higher serum TXNIP was also indicated to be associated with impaired *β* cell function and insulin resistance in PCOS patients [36]. The expressions of toll-like receptor signaling pathway genes (including TLR1, TLR2, TLR4, TLR8, and CD14) were found to be significantly increased in OGC samples of PCOS patients compared with controls [19, 37]. Thus, TXNIP and TLR1 may be involved in PCOS by activating the inflammatory pathway and then inducing cell apoptosis as reported in other diseases. For example, Shan et al. proved that knockdown of TXNIP decreased the inflammatory damage in kidney tissues induced by 2,2′,4,4′-tetrabromodiphenyl ether [38]. The study of Chen et al. revealed that hypoxia induced pancreatic *β* cell death by upregulating the reactive oxygen species- (ROS-) TXNIP-NLR family pyrin domain containing 3 (NLRP3) inflammasome axis [39]. Also, the traditional Chinese medicine Lycium barbarum polysaccharide was demonstrated to attenuate hepatocyte apoptosis induced by ethanol through inhibiting the TXNIP-NLRP3 inflammasome pathway [40]. By using the littermate model, Mohamed et al. verified that TXNIP deletion ameliorated the inflammatory response in high fat diet-induced nonalcoholic steatohepatitis via deactivation of the TLR2-NLRP3 inflammasome axis [41]. Furthermore, miR-382-5p was shown to be negatively correlated with a free androgen index [42]. The PCOS group had a significantly higher free androgen index than controls [43]. In line with these studies, we also identified that TXNIP and TLR1 were expressed higher, while miR-382-5p was expressed lower, in the OGCs of PCOS patients than in normal controls.

Furthermore, *in vitro* exposure of human luteinized mural granulosa cells to dibutyl phthalate was found to induce the high expression of PLK1 [44]. Gestational exposure to dibutyl phthalate was revealed to induce polycystic ovaries and a hormonal profile similar to PCOS [45]. Mass spectrometry analysis showed that culture of OGCs with 10 ng/mL fibroblast growth factor- (FGF-) 8 and FGF-18 significantly triggered upregulation of several proteins, including PLCG2 [46]. Increased intrafollicular FGF-13 levels were positively correlated with elevated total testosterone and increased ovarian volume, but negatively associated with the MII oocyte rate in PCOS patients [47]. These findings indirectly demonstrated their possible roles in PCOS. In agreement with these studies, we also identified that PLK1 and PLCG2 were upregulated in the OGCs of PCOS patients than in normal controls.

Although KLF3-AS1, WWC2-AS, MAPKAPK5-AS1, and MAP3K14 were not previously verified to be associated with PCOS, they may also be important for PCOS because they could, respectively, interact with PCOS-related PLCG2, TXNIP, miR-382-PLCG2, and PLK1 as described above. Also, the expression trend of KLF3-AS1, WWC2-AS, MAPKAPK5-AS1, and MAP3K14 (upregulated) was similar to their interacted genes, while the expression of WWC2-AS2 was inversely correlated with miR-382. These findings indirectly explain the possible regulatory relationships between them.

More importantly, valproic acid, doxorubicin, methotrexate, quercetin, thapsigargin, etoposide, irinotecan, imatinib, gentamicin, and promethazine were predicted to treat PCOS by reversing the expressions of the above crucial DELs, DEMs, and DEGs in this study. Some of them were confirmed to have therapeutic potential for PCOS previously. For example, Khorshidi et al. observed that supplementation of 1,000 mg/day quercetin for 12 weeks significantly decreased testosterone, fasting blood glucose, insulin, and homeostatic model assessment of insulin resistance of PCOS patients compared with the placebo group [48]. Quercetin alleviated the PCOS by suppressing the levels of inflammatory cytokines (IL-1*β*, IL-6, and tumor necrosis factor *α*) [49], increasing the activity of antioxidant enzymes (superoxide dismutase, catalase, glutathione-S-transferase, and reduced glutathione) and improving granulosa cell apoptosis (upregulation of Bcl-2 and downregulation of Bax) [50]. In this study, we predicted that quercetin targeted PLK1, TXNIP, and MAP3K14. Thus, we recommended combining quercetin supplementation and siRNA knockdown of PLK1, TXNIP, and MAP3K14 for the treatment of PCOS, which may be more effective than quercetin-alone treatment.

Some limitations of our study should be acknowledged. First, all of the lncRNAs were not previously confirmed to be associated with PCOS by wet experiments. Clinical samples should be collected to further validate their expressions and associations with androgen, MII oocyte rate, and infertility rate. *In vitro* and *in vivo* studies should be performed to explore their influence on apoptosis and proliferation of OGCs. Second, the coexpression relationship between lncRNAs and mRNAs and the ceRNA axes among lncRNAs, miRNAs, and mRNAs should be verified by immunoprecipitation, cooverexpression, or coknockdown experiments. Third, the target mechanisms of small molecular drugs on lncRNAs, miRNAs, and mRNAs and the effects of their combination therapy also needed further investigation. Fourth, the significantly differential lncRNAs, miRNAs, and mRNAs that are not commonly expressed in multiple datasets or enriched in WGCNA modules also should be given attention to confirm their importance.

## 5. Conclusion

Based on coexpression and ceRNA network analyses, the present study identified that several lncRNAs (KLF3-AS1, WWC2-AS, and MAPKAPK5-AS1), miRNAs (miR-382), and DEGs (PLK1, PLCG2, TXNIP, TLR1, and MAP3K14) were associated with the development of PCOS. In addition, ten small molecular drugs (such as quercetin) were predicted to be therapeutic agents for PCOS by reversing the expressions of these crucial genes. Our study may provide a novel insight into the mechanisms and therapy for PCOS.

## Figures and Tables

**Table 1:** Identified modules by WGCNA analysis.

ID	Color	Module size	Preservation information		#DEGs	Enrichment information	
			*Z*-score	*p* value		Enrichment fold [95% CI]	*P*_hyper_
Module 1	Black	150	7.0208	2.00*E*-10	26	1.998 [1.250-3.084]	2.95*E*-03
Module 2	Blue	332	10.5576	1.90*E*-03	10	0.347 [0.164-0.653]	2.23*E*-04
Module 3	Brown	327	1.5476	1.90*E*-06	17	0.599 [0.342-0.986]	3.85*E*-02
Module 4	Green	213	0.3623	8.60*E*-01	9	0.487 [0.218-0.951]	2.99*E*-02
Module 5	Grey	2,812	0.9776	3.10*E*-01	259	1.046 [0.887-1.231]	5.93*E*-01
Module 6	Magenta	137	5.1445	8.60*E*-06	21	1.767 [1.049-2.844]	2.52*E*-02
Module 7	Pink	143	0.4311	5.10*E*-01	12	0.968 [0.4845-1.761]	9.91*E*-01
Module 8	Purple	136	7.9183	1.40*E*-06	1	0.0848 [0.00213-0.483]	2.89*E*-04
Module 9	Red	152	0.7328	8.20*E*-01	9	0.683 [0.304-1.343]	3.72*E*-01
Module 10	Turquoise	519	6.5919	9.80*E*-04	43	0.955 [0.673-1.327]	8.70*E*-01
Module 11	Yellow	245	19.8755	2.10*E*-11	45	2.118 [1.484-2.967]	4.30*E*-05

DEGs: differentially expressed genes; CI: confidence interval.

**Table 2:** The top 20 genes ranked by topological features.

Symbol	DC	Symbol	CC	Symbol	BC
PLK1	21	CCDC85A	1	CCDC85A	1
TLR1	18	ACAT2	1	ACAT2	1
TAGAP	18	TMEM35A	0.66666667	PLK1	0.35111963
SELL	18	IDH1	0.66666667	TLR1	0.21116561
KIF11	18	AOX1	0.66666667	TAGAP	0.11351916
NKG7	17	MBOAT1	0.66666667	ARHGAP27	0.09620181
KIF20A	17	TLR1	0.51612903	CCL5	0.0824075
MELK	16	PLK1	0.512	MNDA	0.07259953
AURKB	16	SELL	0.46043165	GRAP2	0.07184075
MNDA	16	TAGAP	0.45390071	TBC1D10C	0.06873543
KIF15	15	NKG7	0.45070423	DPEP2	0.06150794
CST7	15	CST7	0.45070423	NKG7	0.06000948
GTSE1	15	TBC1D10C	0.45070423	KIF11	0.0523198
POLQ	15	MNDA	0.44137931	ITPR3	0.05041989
BUB1	15	CCL5	0.43243243	SELL	0.04587418
CCL5	15	RASAL3	0.43243243	GPSM2	0.04246021
NDC80	14	CD300LF	0.42666667	MAP3K14	0.03769369
KIF4A	14	SELPLG	0.42384106	CST7	0.03612451
RASAL3	14	OSM	0.42384106	BUB1	0.03137401
CDCA5	14	MAP3K14	0.42105263	ENPP3	0.03125

DC: degree centrality; BC: betweenness centrality; CC: closeness centrality.

**Table 3:** Function enrichment analysis for the DEGs in the PPI network.

Category	Term	*p* value	Genes
GOTERM_BP_FAT	GO:0000280 ~ nuclear division	2.58*E*-04	KIF11, PLK1, KIF15, BUB1, NDC80, AURKB, CDCA5
GOTERM_BP_FAT	GO:0007067 ~ mitosis	2.58*E*-04	KIF11, PLK1, KIF15, BUB1, NDC80, AURKB, CDCA5
GOTERM_BP_FAT	GO:0000087 ~ M phase of mitotic cell cycle	2.84*E*-04	KIF11, PLK1, KIF15, BUB1, NDC80, AURKB, CDCA5
GOTERM_BP_FAT	GO:0048285 ~ organelle fission	3.20*E*-04	KIF11, PLK1, KIF15, BUB1, NDC80, AURKB, CDCA5
GOTERM_BP_FAT	GO:0000278 ~ mitotic cell cycle	7.09*E*-04	KIF11, PLK1, KIF15, BUB1, NDC80, AURKB, CDCA5, GTSE1
GOTERM_BP_FAT	GO:0001775 ~ cell activation	1.05*E*-03	PREX1, PLCG2, TLR1, IRF1, NFAM1, LTB, ICOSLG
GOTERM_BP_FAT	GO:0022403 ~ cell cycle phase	1.36*E*-03	KIF11, PLK1, KIF15, BUB1, NDC80, AURKB, CDCA5, GTSE1
GOTERM_BP_FAT	GO:0006955 ~ immune response	1.71*E*-03	OSM, POU2AF1, CST7, ENPP3, PLCG2, TLR1, CD300LF, CCL5, LTB, ICOSLG
GOTERM_BP_FAT	GO:0000279 ~ M phase	2.10*E*-03	KIF11, PLK1, KIF15, BUB1, NDC80, AURKB, CDCA5
GOTERM_BP_FAT	GO:0045321 ~ leukocyte activation	2.95*E*-03	PREX1, PLCG2, TLR1, IRF1, NFAM1, ICOSLG
GOTERM_BP_FAT	GO:0051056 ~ regulation of small GTPase-mediated signal transduction	3.51*E*-03	PLEKHG3, TBC1D10C, GMIP, PREX1, RASAL3, ARHGAP27
GOTERM_BP_FAT	GO:0007017 ~ microtubule-based process	3.57*E*-03	KIF4A, KIF11, KIF15, NDC80, GTSE1, KIF20A
GOTERM_BP_FAT	GO:0051301 ~ cell division	6.81*E*-03	KIF11, PLK1, BUB1, NDC80, AURKB, CDCA5
GOTERM_BP_FAT	GO:0022402 ~ cell cycle process	7.62*E*-03	KIF11, PLK1, KIF15, BUB1, NDC80, AURKB, CDCA5, GTSE1
GOTERM_BP_FAT	GO:0046578 ~ regulation of Ras protein signal transduction	1.04*E*-02	PLEKHG3, TBC1D10C, GMIP, PREX1, ARHGAP27
GOTERM_BP_FAT	GO:0007049 ~ cell cycle	1.26*E*-02	TXNIP, KIF11, PLK1, KIF15, BUB1, NDC80, AURKB, CDCA5, GTSE1
GOTERM_BP_FAT	GO:0007242 ~ intracellular signaling cascade	2.84*E*-02	OSM, GMIP, PREX1, PLCG2, TLR1, PPM1L, NDC80, NFAM1, GRAP2, LTB, GTSE1
GOTERM_BP_FAT	GO:0006952 ~ defense response	3.79*E*-02	AOX1, TLR1, MNDA, NFAM1, CCL5, MX2, ICOSLG
KEGG_pathway	hsa00760: nicotinate and nicotinamide metabolism	5.08*E*-03	ENPP3, AOX1, QPRT
KEGG_pathway	hsa05120: epithelial cell signaling in Helicobacter pylori infection	3.72*E*-02	PLCG2, MAP3K14, CCL5

DEGs: differentially expressed genes; PPI: protein-protein interaction; GO: Gene Ontology; KEGG:, Kyoto Encyclopedia of Genes and Genomes.

## Data Availability

All data were downloaded from the Gene Expression Omnibus (GEO, http://www.ncbi.nlm.nih.gov/geo/) under GSE106724, GSE114419, GSE137684, GSE138518, GSE138572, and GSE84376 accession numbers.

## Supplementary Materials

Supplementary 1 Table S1: all differential lncRNAs and miRNAs in four datasets.

Supplementary 2 Table S2: all differential miRNAs in GSE13572 dataset.

Supplementary 3 Table S3: all differential miRNAs in GSE84376 dataset.
