# Supplementary material for: Identification of Crucial lncRNAs, miRNAs, mRNAs, and Potential Therapeutic Compounds for Polycystic Ovary Syndrome by Bioinformatics Analysis
Source: Biomed Res Int. 2020 Nov 6;2020:1817094. doi: 10.1155/2020/1817094 (PMC7666708; doi:10.1155/2020/1817094)
Supplement: Supplementary 1 — Table S1: all differential lncRNAs and miRNAs in four datasets. [file 1817094.f1.docx]

**Table S1 Differentially expressed mRNAs in four datasets**

| Symbol | Type | p | FDR | Q | Qp | tau2 | GSE114419-logFC | GSE106724-logFC | GSE137684-logFC | GSE138515-logFC |
| --- | --- | --- | --- | --- | --- | --- | --- | --- | --- | --- |
| BCAR4 | lncRNA | 1.77E-05 | 0.007183395 | 0.562888634 | 0.904873072 | 0 | 0.036624462 | 0.041287224 | 0.030982353 | 1.053423916 |
| BVES-AS1 | lncRNA | 6.00E-05 | 0.024312032 | 2.508371711 | 0.473780027 | 0 | 0.036091946 | 0.004306117 | 0.089705255 | 1.155415944 |
| CYTOR | lncRNA | 2.35E-05 | 0.009524832 | 2.566222452 | 0.463441982 | 0 | 0.074436277 | 0.083353922 | 0.124325822 | 4.876209676 |
| DLGAP1-AS1 | lncRNA | 5.91E-05 | 0.023954625 | 1.716257128 | 0.633325533 | 0 | 0.066221151 | 0.040613529 | 0.007934683 | 0.050747234 |
| EWSAT1 | lncRNA | 1.68E-05 | 0.006808217 | 2.154288581 | 0.541008122 | 0 | 0.025125231 | 0.022147305 | 0.035083795 | 1.621626657 |
| FAM182A | lncRNA | 2.81E-05 | 0.011377713 | 2.338378871 | 0.505208431 | 0 | 0.018809169 | 0.048869431 | 0.078618741 | 2.808723472 |
| FBXL19-AS1 | lncRNA | 3.44E-06 | 0.001392925 | 2.282793199 | 0.515825286 | 0 | -0.0053226 | -0.44104372 | -0.08075524 | -0.11469118 |
| GNG12-AS1 | lncRNA | 2.54E-05 | 0.010288101 | 0.899671925 | 0.82550698 | 0 | 0.028156627 | 0.026558094 | 0.024020412 | 0.677605515 |
| KLF3-AS1 | lncRNA | 4.38E-06 | 0.001774287 | 1.608944318 | 0.65736346 | 0 | 0.025004194 | 0.025310156 | 0.162421512 | 0.221745279 |
| LINC00663 | lncRNA | 2.48E-05 | 0.010052934 | 1.18853968 | 0.755754262 | 0 | 0.008963238 | 0.153899228 | 0.002940594 | 0.748966293 |
| LINC00910 | lncRNA | 1.17E-05 | 0.004733651 | 0.934518105 | 0.817090806 | 0 | 0.007949735 | 0.202395806 | 0.34404153 | 0.17109367 |
| LINC00937 | lncRNA | 1.61E-05 | 0.00652642 | 2.577139706 | 0.461511354 | 0 | 0.014723861 | 0.081767129 | 0.186292665 | 0.70078651 |
| LINC02145 | lncRNA | 2.51E-05 | 0.010158469 | 1.526369757 | 0.676197714 | 0 | 0.000106947 | 0.012404213 | 0.103112818 | 1.649536707 |
| MAPKAPK5-AS1 | lncRNA | 1.59E-05 | 0.006453796 | 2.2059772 | 0.530772009 | 0 | 0.052331395 | 0.026461551 | 0.001022328 | 0.253570799 |
| MIR181A1HG | lncRNA | 1.50E-05 | 0.006094043 | 0.696565286 | 0.87401153 | 0 | 0.067020751 | 0.019156296 | 0.175270627 | 0.4835309 |
| MYCNOS | lncRNA | 2.74E-05 | 0.011093522 | 0.157760831 | 0.984101504 | 0 | 0.001423806 | 0.028889752 | 0.006897838 | 1.21581988 |
| SMCR5 | lncRNA | 5.10E-05 | 0.020644963 | 2.992192776 | 0.392830464 | 0 | 0.020066113 | 0.394012563 | 0.17317666 | 0.125298954 |
| TPT1-AS1 | lncRNA | 2.13E-06 | 0.000862904 | 2.567878996 | 0.463148621 | 0 | 0.010101594 | 0.093073683 | 0.026899829 | 0.099249866 |
| WWC2-AS2 | lncRNA | 3.98E-05 | 0.016118923 | 1.265751734 | 0.737281279 | 0 | 0.028734751 | 0.165307591 | 0.095014008 | 0.087504454 |
| 2-Mar | mRNA | 7.74E-06 | 0.00313667 | 1.153048975 | 0.764285696 | 0 | -0.02452741 | -0.17906783 | -0.23887159 | -0.70759113 |
| A2ML1 | mRNA | 3.80E-05 | 0.015390011 | 0.533534987 | 0.911464513 | 0 | 0.005087595 | 0.219542518 | 0.707282635 | 1.492040569 |
| AACS | mRNA | 4.33E-05 | 0.017540518 | 0.218493404 | 0.974550021 | 0 | -0.01558574 | -0.04042223 | -0.09182126 | -0.70522826 |
| ABHD12B | mRNA | 3.72E-05 | 0.01507061 | 0.344117024 | 0.951528485 | 0 | 0.031923303 | 0.048123794 | 0.194575365 | 2.235246562 |
| ABHD2 | mRNA | 3.56E-05 | 0.014417591 | 0.411685438 | 0.937818118 | 0 | 0.046217278 | 0.069594796 | 0.124030502 | 0.480412759 |
| ABLIM3 | mRNA | 9.83E-05 | 0.039792911 | 0.62722125 | 0.890173384 | 0 | 0.032764168 | 0.048148291 | 0.062089308 | 0.127082935 |
| ACAT2 | mRNA | 2.28E-05 | 0.009233248 | 0.284841017 | 0.962853799 | 0 | -0.04986115 | -0.10206946 | -0.096015 | -0.4994877 |
| ACRV1 | mRNA | 5.37E-05 | 0.021755284 | 2.626003386 | 0.452949044 | 0 | 0.038195592 | 0.080801696 | 0.281549902 | 0.463551487 |
| ACTR10 | mRNA | 2.88E-06 | 0.001167662 | 2.154612814 | 0.540943467 | 0 | 0.010225438 | 0.166500261 | 0.059679652 | 0.262914146 |
| ADAM32 | mRNA | 6.26E-05 | 0.025362051 | 2.37153371 | 0.49895561 | 0 | -0.02728846 | -0.07030959 | -0.0934743 | -1.93563849 |
| ADAM8 | mRNA | 2.39E-06 | 0.000967681 | 0.425600462 | 0.934904716 | 0 | 0.052089176 | 0.156273497 | 0.169523874 | 1.291774374 |
| ADCK2 | mRNA | 3.91E-05 | 0.015854422 | 0.760272135 | 0.858943865 | 0 | 0.022785831 | 0.151185876 | 0.112580565 | 0.169306888 |
| ADGRB2 | mRNA | 1.70E-05 | 0.006866648 | 0.267795331 | 0.965966964 | 0 | 0.008350666 | 0.081715809 | 0.053693791 | 0.365242366 |
| ADGRD1 | mRNA | 1.84E-05 | 0.007460904 | 0.846628776 | 0.838284559 | 0 | -0.01999578 | -0.03970673 | -0.06113886 | -0.75795452 |
| ADGRE3 | mRNA | 1.36E-05 | 0.005519382 | 0.490212011 | 0.921036468 | 0 | 0.125202017 | 0.152919711 | 0.288310528 | 3.241725876 |
| ADGRG3 | mRNA | 1.94E-05 | 0.007861814 | 1.476962301 | 0.687597535 | 0 | 0.12113451 | 0.03345799 | 0.060181723 | 2.467953511 |
| ADIRF | mRNA | 8.79E-05 | 0.035608947 | 0.638745227 | 0.887508028 | 0 | 0.050371022 | 0.051489332 | 0.118454369 | 2.476617785 |
| ADORA2B | mRNA | 9.06E-07 | 0.00036679 | 1.256619134 | 0.739459142 | 0 | -0.02108383 | -0.2069088 | -0.11262894 | -0.49889559 |
| ADPRHL2 | mRNA | 6.96E-05 | 0.028176476 | 0.233327634 | 0.972037787 | 0 | -0.0699135 | -0.05442219 | -0.03118982 | -0.16103116 |
| AGO4 | mRNA | 5.86E-06 | 0.002375131 | 0.265141208 | 0.966445363 | 0 | 0.044800594 | 0.134773799 | 0.159954727 | 0.78641066 |
| AIM2 | mRNA | 3.16E-05 | 0.012802104 | 0.138926859 | 0.986787969 | 0 | 0.060830124 | 0.221326576 | 0.312172286 | 2.256520762 |
| AKAP12 | mRNA | 1.02E-06 | 0.000412283 | 2.371928018 | 0.498881604 | 0 | 0.133190283 | 0.071691511 | 0.100839013 | 1.366399034 |
| AKAP5 | mRNA | 7.95E-08 | 0.0000322 | 1.712075354 | 0.634252513 | 0 | -0.03811285 | -0.30467975 | -0.38572062 | -1.5926881 |
| AKNA | mRNA | 6.34E-05 | 0.025687139 | 1.949390663 | 0.582977021 | 0 | 0.009639409 | 0.052035787 | 0.232639665 | 1.2178364 |
| ALDOC | mRNA | 3.98E-07 | 0.000161122 | 0.873258447 | 0.831875889 | 0 | -0.085229 | -0.12897915 | -0.121379 | -0.69807953 |
| ALG1L2 | mRNA | 9.09E-05 | 0.036812151 | 0.476434656 | 0.924037261 | 0 | -0.0246054 | -0.01432762 | -0.04693208 | -0.24445237 |
| ALKAL2 | mRNA | 5.47E-05 | 0.022144821 | 0.66125874 | 0.882276189 | 0 | 0.025124955 | 0.188428337 | 0.272754914 | 0.136476766 |
| ALPL | mRNA | 4.42E-06 | 0.001790352 | 1.518565942 | 0.677991998 | 0 | 0.184772557 | 0.267435417 | 0.292130881 | 0.504154475 |
| ANGPTL4 | mRNA | 3.02E-05 | 0.012223486 | 0.434148301 | 0.933101332 | 0 | -0.04626816 | -0.13484396 | -0.101779 | -1.52667838 |
| ANKRD24 | mRNA | 1.50E-06 | 0.000606104 | 1.842347653 | 0.605763254 | 0 | -0.02607807 | -0.11809936 | -0.07797045 | -0.49880829 |
| ANKRD52 | mRNA | 4.40E-05 | 0.017822955 | 0.681618428 | 0.877518726 | 0 | 0.042893171 | 0.058547804 | 0.014958345 | 0.597327065 |
| ANKUB1 | mRNA | 8.10E-05 | 0.032802578 | 0.049356109 | 0.997126516 | 0 | 0.017314674 | 0.296598716 | 0.662083717 | 1.814578445 |
| ANXA3 | mRNA | 7.98E-06 | 0.003233817 | 0.151632297 | 0.984991536 | 0 | 0.15333189 | 0.153477698 | 0.282090639 | 1.659550069 |
| AOX1 | mRNA | 2.34E-06 | 0.000945882 | 2.156175161 | 0.540632 | 0 | -0.13280103 | -0.14507801 | -0.2203121 | -0.32349028 |
| APBB1IP | mRNA | 3.33E-05 | 0.0134755 | 0.40140875 | 0.939951327 | 0 | 0.065944272 | 0.125776765 | 0.225814598 | 3.231335458 |
| APOL6 | mRNA | 2.80E-05 | 0.011331154 | 0.807210584 | 0.847741591 | 0 | 0.01079097 | 0.046114589 | 0.165822676 | 0.593390905 |
| APPBP2 | mRNA | 4.74E-05 | 0.019202446 | 2.508616631 | 0.473735878 | 0 | 0.016984891 | 0.066913568 | 0.005577305 | 0.715870042 |
| AQP9 | mRNA | 9.80E-06 | 0.003967397 | 1.187701311 | 0.755955531 | 0 | 0.116234921 | 0.100254004 | 0.240990902 | 4.744191686 |
| ARAP3 | mRNA | 6.14E-06 | 0.002487916 | 0.780210745 | 0.854194269 | 0 | 0.039715579 | 0.490794808 | 0.233427659 | 0.266267443 |
| ARF5 | mRNA | 8.64E-06 | 0.003498721 | 1.165145347 | 0.761375452 | 0 | 0.024236633 | 0.049738594 | 0.035139293 | 0.193913695 |
| ARHGAP27 | mRNA | 2.47E-05 | 0.010000474 | 0.198079625 | 0.977898715 | 0 | 0.025816793 | 0.089412567 | 0.155358811 | 0.990007745 |
| ARHGAP9 | mRNA | 5.71E-05 | 0.023129088 | 2.133285201 | 0.545208345 | 0 | 0.005000293 | 0.077318356 | 0.087743298 | 1.714383118 |
| ARHGEF6 | mRNA | 9.70E-06 | 0.00392759 | 0.282746034 | 0.963240137 | 0 | 0.043305614 | 0.084190457 | 0.102699178 | 0.367961261 |
| ARID4B | mRNA | 4.53E-05 | 0.018346602 | 0.100470222 | 0.991780936 | 0 | 0.024665356 | 0.030686382 | 0.051836399 | 0.275100731 |
| ARL8B | mRNA | 6.17E-05 | 0.024995735 | 0.476055293 | 0.924119574 | 0 | -0.02462235 | -0.05104489 | -0.0275424 | -0.08494659 |
| ARRB2 | mRNA | 7.74E-05 | 0.031329258 | 1.057411906 | 0.787363665 | 0 | 0.005251244 | 0.012513126 | 0.158077565 | 1.462698679 |
| ASXL2 | mRNA | 8.33E-05 | 0.033750356 | 0.18511845 | 0.979955164 | 0 | 0.024534803 | 0.030170217 | 0.014937168 | 0.748534677 |
| ATP2B4 | mRNA | 5.48E-05 | 0.022179415 | 0.169815769 | 0.982308346 | 0 | 0.038822486 | 0.082080081 | 0.049146967 | 0.406223521 |
| ATP6V0D2 | mRNA | 3.98E-05 | 0.016124064 | 0.294454879 | 0.961067972 | 0 | 0.019360533 | 0.269485205 | 0.3544879 | 3.041265007 |
| ATP9A | mRNA | 7.46E-06 | 0.003021515 | 1.935568366 | 0.585886828 | 0 | 0.001291727 | 0.282352604 | 0.192466259 | 0.8051175 |
| AURKB | mRNA | 2.28E-05 | 0.009251256 | 0.884321268 | 0.829209705 | 0 | 0.039982598 | 0.30502319 | 0.31188478 | 0.61418662 |
| AVIL | mRNA | 1.05E-04 | 0.042720595 | 0.694618583 | 0.874468977 | 0 | 0.028587492 | 0.100782209 | 0.026388465 | 0.497708203 |
| AVPI1 | mRNA | 9.58E-05 | 0.038779263 | 0.521789845 | 0.914078852 | 0 | -0.03565608 | -0.02566304 | -0.0637127 | -0.71655486 |
| AZGP1 | mRNA | 1.80E-05 | 0.00728367 | 0.439136781 | 0.932044211 | 0 | 0.016755331 | 0.114445938 | 0.064222071 | 0.81327618 |
| B3GNT8 | mRNA | 2.04E-06 | 0.000825514 | 1.292967835 | 0.730803607 | 0 | 0.038284451 | 0.063700256 | 0.220746725 | 0.496368435 |
| B3GNTL1 | mRNA | 1.48E-05 | 0.00600417 | 1.148455714 | 0.765391401 | 0 | 0.007397518 | 0.045508944 | 0.059533128 | 0.836066424 |
| B4GALT5 | mRNA | 3.52E-06 | 0.001426405 | 1.772816992 | 0.620868052 | 0 | 0.018622664 | 0.134702108 | 0.102810166 | 0.436835721 |
| BATF | mRNA | 1.36E-06 | 0.000552081 | 0.429645911 | 0.9340525 | 0 | 0.015558783 | 0.271301693 | 0.195450924 | 2.042372462 |
| BCL2L1 | mRNA | 4.23E-05 | 0.017114018 | 1.26665586 | 0.737065784 | 0 | 0.01190404 | 0.040672588 | 0.083798772 | 0.442988206 |
| BCORL1 | mRNA | 6.04E-05 | 0.024442707 | 0.948506404 | 0.813709074 | 0 | 0.010384928 | 0.148870979 | 0.032445897 | 0.113932311 |
| BEST4 | mRNA | 2.24E-05 | 0.009053171 | 2.060319555 | 0.559982003 | 0 | -0.03497184 | -0.02270928 | -0.0929574 | -1.29360589 |
| BICDL1 | mRNA | 9.82E-07 | 0.000397593 | 0.322576989 | 0.955727912 | 0 | 0.01767185 | 0.224807517 | 0.501510784 | 1.411638906 |
| BICRAL | mRNA | 8.66E-06 | 0.003508199 | 0.945118829 | 0.814528167 | 0 | 0.030038084 | 0.34176497 | 0.059932422 | 0.424154333 |
| BMP2K | mRNA | 1.03E-05 | 0.004179699 | 2.75442312 | 0.43105774 | 0 | 0.013743569 | 0.070718176 | 0.040577161 | 0.600496369 |
| BMP4 | mRNA | 1.09E-04 | 0.044263577 | 0.614503774 | 0.893104187 | 0 | 0.005428365 | 0.125066256 | 0.537083884 | 0.558847528 |
| BNIP3 | mRNA | 1.84E-05 | 0.007457113 | 0.856745156 | 0.835851613 | 0 | -0.09826645 | -0.05449716 | -0.09429579 | -0.05716504 |
| BTG2 | mRNA | 1.04E-07 | 0.0000422 | 2.137648377 | 0.544333868 | 0 | 0.0547717 | 0.110267161 | 0.107508747 | 1.760180924 |
| BUB1 | mRNA | 1.02E-05 | 0.00414084 | 1.278144443 | 0.734329321 | 0 | 0.119243248 | 0.21814895 | 0.207390261 | 0.924866111 |
| C10orf82 | mRNA | 1.94E-05 | 0.007841437 | 1.005589741 | 0.799899407 | 0 | 0.00138227 | 0.04109601 | 0.135214455 | 0.599861562 |
| C11orf21 | mRNA | 1.10E-04 | 0.044634632 | 0.207835744 | 0.976314537 | 0 | 0.009386215 | 0.02706864 | 0.143753848 | 3.049894617 |
| C14orf119 | mRNA | 7.98E-05 | 0.032329637 | 2.50468659 | 0.474444707 | 0 | 0.010892483 | 0.034334525 | 0.232586477 | 0.543892774 |
| C16orf72 | mRNA | 2.69E-05 | 0.010894702 | 0.316422913 | 0.956910755 | 0 | 0.01179533 | 0.054325357 | 0.039697373 | 0.473682428 |
| C1orf115 | mRNA | 4.01E-06 | 0.001624017 | 1.826504153 | 0.609184389 | 0 | -0.01098645 | -0.11555369 | -0.22519642 | -0.75031677 |
| C1orf87 | mRNA | 9.97E-05 | 0.04036063 | 1.147359598 | 0.765655311 | 0 | 0.004052595 | 0.136222899 | 0.429046516 | 0.017990055 |
| C2orf40 | mRNA | 3.71E-06 | 0.001502701 | 0.86103919 | 0.834818284 | 0 | 0.044023911 | 0.028232691 | 0.260395885 | 3.153486769 |
| C4orf46 | mRNA | 3.14E-05 | 0.012696901 | 2.499501211 | 0.475381233 | 0 | 0.007883781 | 0.195093389 | 0.035105477 | 0.627118564 |
| C4orf47 | mRNA | 2.03E-06 | 0.000824093 | 0.489727476 | 0.921142375 | 0 | -0.05413307 | -0.25397799 | -0.28234117 | -1.43784941 |
| C4orf48 | mRNA | 9.69E-05 | 0.039226614 | 0.776904481 | 0.854982823 | 0 | -0.02016778 | -0.02380182 | -0.06513884 | -0.06936314 |
| C7 | mRNA | 5.87E-05 | 0.023778315 | 2.034752336 | 0.565225148 | 0 | 0.067272109 | 0.159344013 | 0.022392655 | 0.695964051 |
| CA14 | mRNA | 1.13E-05 | 0.004582978 | 0.531672196 | 0.911880062 | 0 | -0.02840928 | -0.08612071 | -0.0746585 | -1.18340074 |
| CA9 | mRNA | 1.06E-05 | 0.004301488 | 2.686144027 | 0.44258711 | 0 | -0.00436827 | -0.21431727 | -0.89018766 | -2.82292251 |
| CACHD1 | mRNA | 1.15E-05 | 0.004639371 | 2.899756169 | 0.407340426 | 0 | 0.064908327 | 0.112022666 | 0.012676131 | 0.526646357 |
| CAMK1 | mRNA | 1.88E-05 | 0.007607336 | 1.190758058 | 0.755221754 | 0 | 0.029096583 | 0.131688095 | 0.078470574 | 0.613067698 |
| CASTOR3 | mRNA | 7.93E-05 | 0.032112596 | 0.748767009 | 0.861677829 | 0 | 0.00333178 | 0.044259808 | 0.092391983 | 0.311006536 |
| CATSPERG | mRNA | 8.16E-05 | 0.033067482 | 1.193776318 | 0.754497391 | 0 | 0.019023767 | 0.053844699 | 0.004287409 | 0.650491499 |
| CBL | mRNA | 2.63E-06 | 0.00106388 | 2.065167918 | 0.558991607 | 0 | 0.036348016 | 0.100622589 | 0.065853323 | 0.756448606 |
| CBLB | mRNA | 1.07E-04 | 0.043244716 | 0.43719789 | 0.93245549 | 0 | 0.083496931 | 0.034816568 | 0.041073045 | 0.519190683 |
| CBLL1 | mRNA | 1.02E-04 | 0.04142214 | 1.118919569 | 0.772508899 | 0 | 0.013019714 | 0.043235073 | 0.009100534 | 1.141155104 |
| CCDC15 | mRNA | 8.11E-05 | 0.032855654 | 1.788946082 | 0.617343342 | 0 | 0.020587499 | 0.179020307 | 0.033458178 | 0.02753952 |
| CCDC25 | mRNA | 1.37E-05 | 0.005546394 | 1.199295994 | 0.753173166 | 0 | -0.0265726 | -0.05405117 | -0.13618715 | -0.24203964 |
| CCDC85A | mRNA | 8.28E-05 | 0.033519098 | 1.207175826 | 0.751283772 | 0 | -0.0050179 | -0.10533449 | -0.21790672 | -0.0560916 |
| CCL5 | mRNA | 9.65E-05 | 0.039080656 | 0.2736474 | 0.964906023 | 0 | 0.0795077 | 0.094613993 | 0.179485249 | 6.480954827 |
| CCND2 | mRNA | 3.88E-05 | 0.015699934 | 2.326611052 | 0.507442114 | 0 | 0.024263045 | 0.118803894 | 0.037424229 | 0.247747403 |
| CD177 | mRNA | 1.08E-05 | 0.004373993 | 0.52561535 | 0.91322885 | 0 | 0.132831243 | 0.156497708 | 0.592747245 | 1.776693027 |
| CD1D | mRNA | 1.98E-07 | 0.000080087 | 0.62383189 | 0.890955586 | 0 | 0.029586452 | 0.171385238 | 0.449948121 | 2.813407282 |
| CD274 | mRNA | 4.13E-05 | 0.016720216 | 0.242488057 | 0.970455229 | 0 | 0.078587922 | 0.149458076 | 0.279732896 | 0.606807473 |
| CD300LF | mRNA | 6.54E-05 | 0.026477585 | 0.686838623 | 0.876295185 | 0 | 0.019646146 | 0.116401455 | 0.296299464 | 3.87287417 |
| CDC42EP2 | mRNA | 1.04E-04 | 0.042217326 | 0.252137885 | 0.968763465 | 0 | 0.029838815 | 0.068649165 | 0.111382385 | 0.446484982 |
| CDCA5 | mRNA | 7.27E-05 | 0.029430386 | 0.095673017 | 0.992351574 | 0 | 0.007496637 | 0.38081602 | 0.143041911 | 0.50156529 |
| CDH1 | mRNA | 7.29E-06 | 0.002953275 | 0.08836785 | 0.993195816 | 0 | 0.042892545 | 0.152429962 | 0.843893776 | 3.866225208 |
| CDK14 | mRNA | 6.96E-07 | 0.000281964 | 2.058480731 | 0.560357951 | 0 | 0.017063586 | 0.085424095 | 0.091084546 | 0.798205097 |
| CEACAM3 | mRNA | 7.29E-07 | 0.000295233 | 0.340412478 | 0.952257109 | 0 | 0.10188776 | 0.296075794 | 0.504925276 | 3.045210315 |
| CELF2 | mRNA | 1.15E-04 | 0.04646479 | 0.529180765 | 0.912435315 | 0 | 0.05567047 | 0.039023707 | 0.12964621 | 1.142268143 |
| CENPT | mRNA | 7.24E-05 | 0.029313335 | 0.162259361 | 0.983438824 | 0 | -0.01921188 | -0.03490589 | -0.07038244 | -0.12573876 |
| CEP19 | mRNA | 1.43E-05 | 0.005798029 | 1.883692855 | 0.59689391 | 0 | 0.0532004 | 0.20190063 | 0.119788107 | 0.338352633 |
| CEP68 | mRNA | 2.00E-05 | 0.008101128 | 2.078245751 | 0.556326318 | 0 | 0.004988734 | 0.123053343 | 0.049075712 | 1.278529652 |
| CEP76 | mRNA | 4.20E-06 | 0.001700787 | 2.917912112 | 0.404455442 | 0 | 0.015604765 | 0.15836377 | 0.043842242 | 1.575863226 |
| CEP85L | mRNA | 1.29E-05 | 0.00522178 | 0.880872641 | 0.830041055 | 0 | 0.013080163 | 0.121210057 | 0.321102376 | 0.455059406 |
| CFAP43 | mRNA | 3.32E-05 | 0.013453227 | 1.386486061 | 0.708705509 | 0 | 0.018627388 | 0.041007574 | 0.046224341 | 2.219462246 |
| CFAP58 | mRNA | 1.63E-06 | 0.000658706 | 1.888197644 | 0.595932694 | 0 | 0.006203494 | 0.280288268 | 0.486670864 | 1.624109485 |
| CFHR1 | mRNA | 2.76E-05 | 0.011170505 | 1.082060303 | 0.781406467 | 0 | 0.055517143 | 0.095630044 | 0.112491839 | 0.569190114 |
| CHI3L1 | mRNA | 6.45E-06 | 0.002612549 | 0.290001037 | 0.961897914 | 0 | 0.025490129 | 0.374610865 | 0.680586193 | 1.934432487 |
| CHIC2 | mRNA | 5.35E-06 | 0.002167567 | 1.002114911 | 0.80074021 | 0 | 0.014614787 | 0.036387482 | 0.065720231 | 0.773189464 |
| CHP2 | mRNA | 7.03E-06 | 0.002848545 | 1.907334272 | 0.591860751 | 0 | 0.006009821 | 0.285142475 | 0.365875493 | 1.077579837 |
| CHPF | mRNA | 1.20E-04 | 0.048450384 | 0.750711003 | 0.86121624 | 0 | -0.03286593 | -0.02809234 | -0.01828886 | -0.51789257 |
| CHPT1 | mRNA | 1.94E-05 | 0.00785281 | 1.701569184 | 0.636584986 | 0 | 0.008100956 | 0.040566484 | 0.033601769 | 0.926827832 |
| CHRD | mRNA | 1.41E-05 | 0.005697564 | 1.690814063 | 0.638977967 | 0 | 0.003437426 | 0.043929426 | 0.109050322 | 1.222900322 |
| CHRNB4 | mRNA | 6.75E-06 | 0.002732443 | 2.068905492 | 0.558228962 | 0 | 0.037793376 | 0.526451718 | 0.246263901 | 0.869247298 |
| CHST1 | mRNA | 4.09E-05 | 0.016572837 | 1.401113623 | 0.705273713 | 0 | 0.044014094 | 0.356762951 | 0.042022557 | 0.678167863 |
| CHST6 | mRNA | 9.48E-05 | 0.038395413 | 0.201016607 | 0.977425016 | 0 | 0.013212467 | 0.01625566 | 0.043461632 | 0.326376691 |
| CIP2A | mRNA | 3.53E-05 | 0.014289167 | 1.211031076 | 0.750359838 | 0 | 0.018889547 | 0.134861975 | 0.09812109 | 1.647434954 |
| CKAP2L | mRNA | 3.28E-05 | 0.013300635 | 0.669232922 | 0.880415689 | 0 | 0.039088787 | 0.220001686 | 0.583520163 | 0.196480748 |
| CLC | mRNA | 9.29E-05 | 0.037638139 | 0.431873364 | 0.933582284 | 0 | 0.020453128 | 0.162813646 | 0.267786223 | 3.348237106 |
| CLDN23 | mRNA | 3.87E-05 | 0.015691404 | 1.476714903 | 0.687654851 | 0 | 0.008387304 | 0.40735346 | 0.101633601 | 1.748059028 |
| CLEC2B | mRNA | 1.36E-05 | 0.005520804 | 0.535295585 | 0.911071449 | 0 | 0.065391159 | 0.142194076 | 0.268648414 | 0.966934557 |
| CLIP2 | mRNA | 7.50E-05 | 0.030359208 | 0.043731018 | 0.997599439 | 0 | 0.012904469 | 0.093097636 | 0.064047663 | 0.322547344 |
| CMTM6 | mRNA | 6.69E-06 | 0.00271017 | 1.122766049 | 0.771581305 | 0 | 0.014117188 | 0.056317264 | 0.058576504 | 0.500236337 |
| CNGA4 | mRNA | 9.81E-05 | 0.039725145 | 0.270989591 | 0.965388903 | 0 | -0.01283583 | -0.12668158 | -0.16338469 | -2.06375057 |
| CNNM1 | mRNA | 2.97E-06 | 0.001201782 | 1.900860893 | 0.593236111 | 0 | -0.00504853 | -0.36106054 | -0.18571549 | -2.16140895 |
| CNOT10 | mRNA | 2.86E-05 | 0.011566203 | 1.586885298 | 0.662367133 | 0 | 0.005327264 | 0.008856055 | 0.01423181 | 0.611087785 |
| CNOT6 | mRNA | 7.14E-05 | 0.028922377 | 2.239323992 | 0.52424436 | 0 | 0.001242503 | 0.097568875 | 0.007261347 | 0.452674044 |
| CNTRL | mRNA | 9.50E-05 | 0.038474079 | 2.987440905 | 0.393565598 | 0 | 0.00849117 | 0.002883446 | 0.081995315 | 0.762075953 |
| COBLL1 | mRNA | 1.72E-06 | 0.000698038 | 2.921766544 | 0.403845182 | 0 | -0.06410097 | -0.13706818 | -0.1207488 | -0.1791452 |
| COL14A1 | mRNA | 4.78E-06 | 0.001936783 | 1.863101875 | 0.601300456 | 0 | 0.026919386 | 0.057920821 | 0.038731501 | 0.161641904 |
| COL24A1 | mRNA | 2.04E-05 | 0.00826462 | 0.018384165 | 0.999340688 | 0 | 0.017570845 | 0.073478734 | 0.205646856 | 1.20684035 |
| COL4A3 | mRNA | 6.83E-06 | 0.002765615 | 1.753075571 | 0.625199053 | 0 | 0.023090723 | 0.255835042 | 0.899661876 | 0.575941277 |
| COL4A4 | mRNA | 3.08E-05 | 0.012486968 | 0.226534019 | 0.973196231 | 0 | 0.010910585 | 0.116834987 | 0.242007686 | 0.479317437 |
| COL9A1 | mRNA | 2.55E-05 | 0.010317032 | 0.534137565 | 0.911330018 | 0 | 0.015886903 | 0.049345462 | 0.033148933 | 1.612427799 |
| CORO6 | mRNA | 6.10E-06 | 0.002470856 | 0.366739199 | 0.947024354 | 0 | -0.03192144 | -0.01635109 | -0.05379029 | -0.80233793 |
| COX20 | mRNA | 8.97E-06 | 0.003634253 | 2.074870913 | 0.557013254 | 0 | -0.02457166 | -0.07287236 | -0.07982607 | -0.00332366 |
| CPB1 | mRNA | 1.12E-04 | 0.045491423 | 0.692589998 | 0.874945457 | 0 | 0.011137385 | 0.158296401 | 0.25354498 | 1.38059424 |
| CPNE7 | mRNA | 8.18E-05 | 0.03311108 | 1.560381343 | 0.668406066 | 0 | -0.02762694 | -0.05768037 | -0.03920864 | -0.6129162 |
| CRIM1 | mRNA | 5.42E-05 | 0.021932992 | 0.104007191 | 0.991352247 | 0 | 0.031995535 | 0.112401045 | 0.100328507 | 0.743897671 |
| CRIP2 | mRNA | 7.04E-07 | 0.000285281 | 1.117690179 | 0.772805412 | 0 | 0.010892666 | 0.089956281 | 0.271373073 | 0.742299985 |
| CRISPLD2 | mRNA | 1.09E-04 | 0.044069283 | 0.553167633 | 0.907064728 | 0 | -0.06511598 | -0.07631973 | -0.07606537 | -0.02578271 |
| CRLF3 | mRNA | 5.51E-05 | 0.022323477 | 0.726366609 | 0.866985425 | 0 | 0.000385578 | 0.03108743 | 0.118204348 | 0.948171087 |
| CSF2RB | mRNA | 5.03E-06 | 0.00203867 | 0.663454333 | 0.881764297 | 0 | 0.115790287 | 0.143373151 | 0.26131436 | 8.065715954 |
| CST7 | mRNA | 1.23E-05 | 0.004981045 | 0.123807465 | 0.988834825 | 0 | 0.149308028 | 0.177193143 | 0.322348656 | 7.47704531 |
| CTDP1 | mRNA | 6.70E-05 | 0.027135817 | 1.926836374 | 0.587730068 | 0 | -0.03941134 | -0.02613842 | -0.00121501 | -0.32930742 |
| CXCR1 | mRNA | 4.87E-07 | 0.000197138 | 0.432559951 | 0.933437206 | 0 | 0.226550234 | 0.086983435 | 0.302174651 | 3.613848354 |
| CXCR6 | mRNA | 8.59E-05 | 0.034774429 | 0.978113425 | 0.806547654 | 0 | 0.04987215 | 0.022015826 | 0.313299185 | 2.338833911 |
| CXorf38 | mRNA | 8.74E-05 | 0.035410862 | 0.339435618 | 0.952448808 | 0 | 0.004584212 | 0.022274923 | 0.034047888 | 0.310532905 |
| CYP3A5 | mRNA | 1.03E-04 | 0.041570468 | 1.074280893 | 0.783286123 | 0 | 0.046154849 | 0.402407245 | 0.097482847 | 1.636653601 |
| CYP3A7 | mRNA | 9.54E-06 | 0.003865037 | 1.655509309 | 0.646869959 | 0 | 0.093599868 | 0.180892133 | 0.129810182 | 2.394021457 |
| CYP4F12 | mRNA | 4.69E-05 | 0.018975927 | 0.454021148 | 0.928870206 | 0 | 0.021873567 | 0.207838438 | 0.181638181 | 1.140273573 |
| CYP51A1 | mRNA | 1.07E-04 | 0.043385461 | 0.354462455 | 0.949480164 | 0 | -0.01795978 | -0.05041274 | -0.0679681 | -0.27253333 |
| DAPK1 | mRNA | 1.76E-06 | 0.000714151 | 1.188169069 | 0.755843234 | 0 | 0.031274996 | 0.225568547 | 0.238758516 | 0.732997775 |
| DAPK2 | mRNA | 1.78E-07 | 0.000072031 | 1.273798918 | 0.735363984 | 0 | 0.018887285 | 0.332570677 | 0.347017119 | 1.918272533 |
| DDIT4L | mRNA | 4.09E-05 | 0.016556725 | 0.498263958 | 0.919272647 | 0 | 0.048597618 | 0.515812259 | 0.170827474 | 0.483056836 |
| DDX10 | mRNA | 1.16E-04 | 0.047033457 | 0.281265149 | 0.963512609 | 0 | -0.0351081 | -0.01738379 | -0.05165481 | -0.14980834 |
| DDX41 | mRNA | 1.58E-05 | 0.006383755 | 0.399861483 | 0.940271108 | 0 | -0.07109284 | -0.06076583 | -0.02081048 | -0.24415416 |
| DDX55 | mRNA | 1.18E-04 | 0.047741447 | 1.718657289 | 0.632793851 | 0 | -0.02446963 | -0.03219825 | -0.00117599 | -0.60854628 |
| DDX60 | mRNA | 6.19E-05 | 0.025069188 | 1.424272418 | 0.699855135 | 0 | 0.0173685 | 0.031956959 | 0.050227531 | 1.110745427 |
| DENND3 | mRNA | 3.85E-05 | 0.01558241 | 0.63888031 | 0.887476733 | 0 | 0.110083219 | 0.031150279 | 0.111780857 | 0.301755885 |
| DES | mRNA | 7.61E-05 | 0.030834044 | 0.312368884 | 0.957685688 | 0 | 0.031977548 | 0.062657291 | 0.039706895 | 0.555618472 |
| DEUP1 | mRNA | 4.20E-05 | 0.017023979 | 0.327307614 | 0.954813441 | 0 | 0.017159234 | 0.226540424 | 0.292494384 | 2.56473789 |
| DHRS7 | mRNA | 1.26E-05 | 0.005115629 | 0.305973217 | 0.958901193 | 0 | 0.015500077 | 0.042334294 | 0.071603756 | 0.760480156 |
| DIABLO | mRNA | 3.27E-05 | 0.013254194 | 0.382065179 | 0.943921975 | 0 | -0.02936951 | -0.01558947 | -0.03605522 | -0.40930865 |
| DIP2B | mRNA | 3.73E-06 | 0.001509336 | 0.992665976 | 0.803026568 | 0 | 0.034404582 | 0.103462448 | 0.078966896 | 0.70481808 |
| DKC1 | mRNA | 1.88E-05 | 0.007594067 | 0.952143058 | 0.812829672 | 0 | -0.04205149 | -0.08015847 | -0.07336501 | -0.1187775 |
| DNAH2 | mRNA | 7.58E-05 | 0.030682874 | 0.334599314 | 0.953395189 | 0 | -0.0907603 | -0.05339235 | -0.09096449 | -0.70708842 |
| DOCK8 | mRNA | 9.35E-05 | 0.037879348 | 0.865357015 | 0.833778869 | 0 | 0.032449445 | 0.019013033 | 0.098145721 | 1.826074132 |
| DPEP2 | mRNA | 7.22E-05 | 0.029244148 | 0.129657743 | 0.988054996 | 0 | 0.020494802 | 0.162396389 | 0.264336479 | 1.716870256 |
| DPY19L2 | mRNA | 2.73E-05 | 0.011062933 | 0.224248884 | 0.973582907 | 0 | -0.05636511 | -0.06925362 | -0.07428948 | -1.15303533 |
| DRD5 | mRNA | 5.08E-05 | 0.020583831 | 1.34655935 | 0.718107897 | 0 | 0.002593627 | 0.092460613 | 0.223855547 | 0.965903475 |
| DTNBP1 | mRNA | 9.97E-05 | 0.040378637 | 0.404888197 | 0.939230867 | 0 | 0.014884126 | 0.016848609 | 0.050801192 | 0.29204952 |
| DUOX2 | mRNA | 4.86E-05 | 0.019673965 | 1.13059175 | 0.769694714 | 0 | -0.04984447 | -0.05145055 | -0.18158891 | -1.11264767 |
| DUOXA2 | mRNA | 2.04E-06 | 0.000825988 | 1.081374936 | 0.781572044 | 0 | -0.04078457 | -0.18381273 | -0.27132054 | -0.56785051 |
| DUSP1 | mRNA | 1.27E-05 | 0.005161596 | 1.172832286 | 0.759527351 | 0 | 0.007359787 | 0.058285417 | 0.045233553 | 0.864529298 |
| EBP | mRNA | 9.62E-06 | 0.00389584 | 1.077941874 | 0.782401493 | 0 | -0.01384897 | -0.08602061 | -0.08137591 | -0.88470771 |
| EBPL | mRNA | 2.02E-05 | 0.008174107 | 2.403767745 | 0.492933644 | 0 | 0.050458603 | 0.032685463 | 0.018241391 | 1.252710543 |
| ECHDC3 | mRNA | 1.18E-04 | 0.047988343 | 0.600261701 | 0.89637246 | 0 | -0.00785126 | -0.04462377 | -0.04669799 | -0.16198567 |
| EFNB1 | mRNA | 1.65E-06 | 0.000669131 | 2.325307709 | 0.507689967 | 0 | 0.042192865 | 0.255594383 | 0.092019439 | 0.480636038 |
| EIF4E1B | mRNA | 5.72E-05 | 0.023169368 | 0.521792364 | 0.914078293 | 0 | 0.008232605 | 0.24514631 | 0.362842408 | 0.988431151 |
| EML1 | mRNA | 1.54E-05 | 0.006239693 | 2.128303945 | 0.546207939 | 0 | 0.043000391 | 0.09112322 | 0.058979437 | 1.291286235 |
| ENDOG | mRNA | 4.54E-05 | 0.018396361 | 2.321821849 | 0.508353315 | 0 | -0.07853637 | -0.00721226 | -0.08825167 | -0.09360977 |
| ENO2 | mRNA | 1.58E-05 | 0.006407924 | 0.280240492 | 0.963700838 | 0 | -0.10359352 | -0.09459951 | -0.10904046 | -0.72429581 |
| ENPP3 | mRNA | 6.99E-06 | 0.002830538 | 1.61299403 | 0.656447113 | 0 | -0.05295672 | -0.18136045 | -0.22172493 | -1.06471799 |
| ENTPD5 | mRNA | 1.84E-05 | 0.007455218 | 0.954393456 | 0.812285448 | 0 | 0.006399688 | 0.088612257 | 0.050246415 | 0.798292552 |
| EPHA1 | mRNA | 1.19E-04 | 0.048220074 | 0.265030194 | 0.966465335 | 0 | 0.021785256 | 0.255264247 | 0.318138351 | 0.226369366 |
| EPS8L2 | mRNA | 9.33E-06 | 0.003778789 | 0.868843857 | 0.832939238 | 0 | 0.011032756 | 0.051035319 | 0.065535653 | 0.618225971 |
| ERAS | mRNA | 2.87E-05 | 0.011633495 | 0.770421841 | 0.856527858 | 0 | -0.01399327 | -0.19691881 | -0.12854671 | -1.95527193 |
| ERCC6L | mRNA | 1.17E-04 | 0.047450005 | 0.27584567 | 0.964505345 | 0 | 0.018141785 | 0.019211639 | 0.310666021 | 0.980942947 |
| ERG28 | mRNA | 6.76E-05 | 0.027395508 | 0.539232396 | 0.910191449 | 0 | -0.01490586 | -0.04062907 | -0.07712693 | -0.08103353 |
| ESF1 | mRNA | 8.00E-05 | 0.032385082 | 0.785919632 | 0.852831822 | 0 | -0.01291555 | -0.10570451 | -0.10006238 | -0.12090725 |
| ESRRA | mRNA | 5.95E-05 | 0.024083499 | 0.782505816 | 0.853646672 | 0 | 0.000373587 | 0.03709597 | 0.040911858 | 0.533606441 |
| ETNK2 | mRNA | 2.61E-05 | 0.010551607 | 0.665776046 | 0.88122269 | 0 | 0.007869987 | 0.161496425 | 0.065631536 | 0.222617542 |
| EXOC8 | mRNA | 9.88E-05 | 0.040003317 | 0.241256563 | 0.970669328 | 0 | 0.00658009 | 0.043623842 | 0.03037817 | 0.629886513 |
| F2RL3 | mRNA | 9.43E-05 | 0.038192115 | 0.518643011 | 0.914776943 | 0 | -0.01093833 | -0.06120397 | -0.03551892 | -1.82218634 |
| F5 | mRNA | 8.96E-06 | 0.003629988 | 0.913906803 | 0.822070547 | 0 | 0.097174782 | 0.173357772 | 0.099551782 | 0.917189852 |
| FADS1 | mRNA | 1.17E-04 | 0.047467065 | 0.164665583 | 0.983081174 | 0 | -0.01642326 | -0.02766888 | -0.08506029 | -0.62963698 |
| FADS2 | mRNA | 9.39E-05 | 0.038013933 | 0.539901726 | 0.910041685 | 0 | -0.04515856 | -0.01498857 | -0.10471214 | -0.58456968 |
| FAM120B | mRNA | 4.76E-05 | 0.019284902 | 1.246550427 | 0.741862592 | 0 | 0.015761356 | 0.035129187 | 0.004265469 | 0.434436816 |
| FAM129A | mRNA | 4.57E-05 | 0.018520046 | 0.334111568 | 0.953490382 | 0 | 0.171093157 | 0.0507705 | 0.120945663 | 0.876874533 |
| FAM13C | mRNA | 9.07E-05 | 0.036734907 | 0.073857404 | 0.994778354 | 0 | 0.074824147 | 0.176444864 | 0.684378963 | 1.054500336 |
| FAM151B | mRNA | 5.81E-05 | 0.023535684 | 0.496290824 | 0.919705542 | 0 | -0.01154086 | -0.16360494 | -0.1101169 | -0.66096103 |
| FAM162A | mRNA | 1.79E-05 | 0.007246707 | 0.906492791 | 0.823860673 | 0 | -0.08691946 | -0.05782092 | -0.06721639 | -0.15126067 |
| FAM192A | mRNA | 1.48E-05 | 0.00598711 | 0.18692554 | 0.979671844 | 0 | 0.003326227 | 0.026424725 | 0.029104958 | 0.535638578 |
| FAM19A2 | mRNA | 3.03E-07 | 0.000122737 | 2.549270838 | 0.466452507 | 0 | 0.117949388 | 0.385978841 | 0.353183126 | 0.476417368 |
| FAM210B | mRNA | 1.34E-06 | 0.000543077 | 0.083374094 | 0.993755053 | 0 | 0.029902507 | 0.036751029 | 0.067318727 | 0.388501393 |
| FAM217A | mRNA | 2.07E-05 | 0.00838404 | 0.661622989 | 0.882191286 | 0 | 0.023091198 | 0.035926616 | 0.045383778 | 2.951588135 |
| FAM78A | mRNA | 1.16E-05 | 0.004713771 | 1.422664293 | 0.7002308 | 0 | 0.002280509 | 0.214833582 | 0.181142536 | 0.482183835 |
| FASN | mRNA | 9.98E-05 | 0.040419392 | 0.344319231 | 0.95148864 | 0 | -0.03610498 | -0.03521025 | -0.08817885 | -0.65629679 |
| FAXDC2 | mRNA | 8.09E-06 | 0.003276941 | 1.516403338 | 0.678489656 | 0 | 0.041945517 | 0.072940154 | 0.065986122 | 0.286512195 |
| FBF1 | mRNA | 1.13E-04 | 0.045798029 | 0.736080521 | 0.864686396 | 0 | 0.004498975 | 0.067195848 | 0.040254213 | 0.520332781 |
| FBXL3 | mRNA | 2.62E-05 | 0.010595204 | 1.045935648 | 0.790138785 | 0 | -0.01435261 | -0.03242455 | -0.05177773 | -0.81453074 |
| FBXW10 | mRNA | 8.38E-05 | 0.033925221 | 0.963964876 | 0.809970441 | 0 | -0.00036593 | -0.08518166 | -0.26334026 | -5.20417264 |
| FCAR | mRNA | 5.40E-05 | 0.021889869 | 0.774390425 | 0.855582182 | 0 | 0.179618262 | 0.027123069 | 0.291158153 | 4.762121524 |
| FER1L6 | mRNA | 1.00E-05 | 0.004063122 | 1.292719769 | 0.730862561 | 0 | 0.032482161 | 0.198818535 | 0.169645399 | 3.154711291 |
| FFAR2 | mRNA | 6.09E-08 | 0.00002465 | 1.413280986 | 0.702424568 | 0 | 0.333101099 | 0.059992836 | 0.325119553 | 5.103659461 |
| FGFBP2 | mRNA | 3.17E-05 | 0.012823903 | 0.837094547 | 0.840575463 | 0 | 0.073404319 | 0.13461737 | 0.325923477 | 0.549046146 |
| FIBIN | mRNA | 2.35E-08 | 0.0000095 | 1.96310662 | 0.580099265 | 0 | 0.210416282 | 0.65633269 | 0.742653062 | 0.701895974 |
| FJX1 | mRNA | 1.16E-04 | 0.046947683 | 0.563284708 | 0.904783597 | 0 | -0.02442475 | -0.04378054 | -0.13022459 | -0.59588627 |
| FLOT2 | mRNA | 2.15E-05 | 0.008707706 | 0.595511965 | 0.897459034 | 0 | 0.016875552 | 0.038076548 | 0.110081277 | 0.246449899 |
| FLRT1 | mRNA | 7.95E-05 | 0.032185575 | 0.791839798 | 0.851417834 | 0 | 0.042218663 | 0.02606471 | 0.441002101 | 1.065632521 |
| FMN2 | mRNA | 8.95E-05 | 0.036261018 | 0.11645903 | 0.989791642 | 0 | 0.013277043 | 0.051233713 | 0.2081888 | 1.755597471 |
| FOXO1 | mRNA | 1.10E-04 | 0.04444555 | 1.105887123 | 0.775653102 | 0 | 0.061743177 | 0.059819621 | 0.022190809 | 0.014338147 |
| FRAT1 | mRNA | 1.43E-05 | 0.005807507 | 0.41843966 | 0.936407496 | 0 | 0.024407257 | 0.312659089 | 0.218116884 | 0.585420288 |
| FRAT2 | mRNA | 1.94E-05 | 0.007848072 | 0.223241358 | 0.97375291 | 0 | 0.064979209 | 0.023228529 | 0.19579865 | 0.276983208 |
| FRY | mRNA | 2.17E-06 | 0.000879538 | 0.297123566 | 0.960568549 | 0 | 0.113838416 | 0.088474663 | 0.286436622 | 2.751444551 |
| FSHR | mRNA | 5.93E-07 | 0.000240262 | 0.514354229 | 0.915726721 | 0 | 0.130157471 | 0.353994543 | 0.774604379 | 1.037694988 |
| FYN | mRNA | 2.41E-05 | 0.00976353 | 0.392461028 | 0.941795429 | 0 | 0.075914333 | 0.069903101 | 0.109994615 | 0.285548811 |
| GAL3ST2 | mRNA | 1.08E-04 | 0.043557009 | 0.491940313 | 0.920658488 | 0 | -0.00558561 | -0.04709959 | -0.03854401 | -0.70055044 |
| GALNT15 | mRNA | 9.68E-05 | 0.039185859 | 0.142148771 | 0.986338811 | 0 | -0.05795162 | -0.13917551 | -0.09377695 | -1.04568434 |
| GASK1A | mRNA | 9.22E-05 | 0.037359966 | 0.564269833 | 0.904560993 | 0 | 0.010710613 | 0.373462237 | 0.217467481 | 0.091868318 |
| GATA1 | mRNA | 4.66E-05 | 0.018885414 | 1.23669913 | 0.744216464 | 0 | 0.022967057 | 0.139745708 | 0.116466911 | 1.671212948 |
| GATM | mRNA | 4.44E-05 | 0.017992608 | 1.689796342 | 0.63920468 | 0 | 0.015024936 | 0.243189133 | 0.057749559 | 0.909052106 |
| GBP1 | mRNA | 1.20E-04 | 0.048628566 | 0.962803894 | 0.810251268 | 0 | 0.033402038 | 0.016562207 | 0.075559025 | 0.729474277 |
| GBP2 | mRNA | 5.62E-05 | 0.022750924 | 0.02240887 | 0.999113802 | 0 | 0.104238395 | 0.092827859 | 0.136366007 | 0.806111986 |
| GBP4 | mRNA | 1.59E-05 | 0.006440622 | 0.909310889 | 0.823180316 | 0 | 0.0435464 | 0.049473117 | 0.126474699 | 0.263997544 |
| GIMAP4 | mRNA | 9.11E-05 | 0.036886551 | 0.497739188 | 0.919387821 | 0 | 0.077048603 | 0.092164586 | 0.157385958 | 4.613091805 |
| GIMAP8 | mRNA | 6.20E-05 | 0.025113734 | 0.087411209 | 0.993304095 | 0 | 0.040997441 | 0.273095559 | 0.218243381 | 2.698859208 |
| GIPC2 | mRNA | 5.87E-05 | 0.02376552 | 0.919926253 | 0.820616677 | 0 | 0.113758779 | 0.108526904 | 0.189222542 | 0.865099009 |
| GLIS2 | mRNA | 2.81E-05 | 0.011388968 | 0.579556667 | 0.90109604 | 0 | -0.03034968 | -0.07553114 | -0.16057523 | -0.14076332 |
| GLT1D1 | mRNA | 1.34E-06 | 0.000541655 | 0.488388332 | 0.921434938 | 0 | 0.196068896 | 0.307515247 | 0.231854802 | 3.30424561 |
| GM2A | mRNA | 2.19E-05 | 0.008889205 | 1.07873108 | 0.782210807 | 0 | -0.04439097 | -0.13993884 | -0.11085035 | -0.34731466 |
| GMIP | mRNA | 2.23E-05 | 0.009032319 | 0.075019985 | 0.994656436 | 0 | 0.048619232 | 0.076896633 | 0.181725323 | 0.541043075 |
| GNA14 | mRNA | 7.37E-05 | 0.029829874 | 0.43195854 | 0.933564289 | 0 | 0.030094861 | 0.106164701 | 0.464023773 | 0.66243537 |
| GPBP1L1 | mRNA | 2.36E-05 | 0.00957587 | 1.300929692 | 0.728912323 | 0 | 0.001162737 | 0.061848112 | 0.027225181 | 0.214272901 |
| GPC5 | mRNA | 7.40E-06 | 0.002998768 | 2.103513326 | 0.551202321 | 0 | 0.08693398 | 0.315844229 | 0.110637505 | 0.855272186 |
| GPR132 | mRNA | 6.43E-05 | 0.026058194 | 1.001276904 | 0.800942984 | 0 | 0.005310843 | 0.186633954 | 0.178998159 | 2.241911782 |
| GPR150 | mRNA | 9.87E-05 | 0.03995972 | 0.576373283 | 0.901819219 | 0 | 0.024330858 | 0.004867555 | 0.045594398 | 0.553971958 |
| GPR25 | mRNA | 1.05E-04 | 0.042549996 | 0.500661145 | 0.918746137 | 0 | 0.015516118 | 0.050046038 | 0.019703302 | 1.323136738 |
| GPR27 | mRNA | 1.18E-05 | 0.004794806 | 1.534147129 | 0.67441191 | 0 | 0.013295901 | 0.292814342 | 0.754559543 | 0.820455046 |
| GPSM2 | mRNA | 2.96E-05 | 0.01200218 | 0.533277206 | 0.911522039 | 0 | 0.029908924 | 0.084121501 | 0.165616755 | 0.961017297 |
| GPSM3 | mRNA | 8.41E-06 | 0.003404417 | 0.07412589 | 0.994750277 | 0 | 0.019239402 | 0.029196685 | 0.163793149 | 2.192592486 |
| GRAP | mRNA | 2.59E-05 | 0.010475785 | 1.367483769 | 0.713174099 | 0 | 0.001299564 | 0.167441575 | 0.164493661 | 1.877606957 |
| GRAP2 | mRNA | 6.19E-05 | 0.025080561 | 0.112752535 | 0.990264341 | 0 | 0.046921514 | 0.153613249 | 0.499178439 | 0.96175754 |
| GRIK1 | mRNA | 3.70E-06 | 0.001499858 | 2.406669771 | 0.492394255 | 0 | 0.036463646 | 0.400146379 | 0.342395593 | 0.665858914 |
| GRIN3B | mRNA | 5.77E-05 | 0.023363662 | 1.121971904 | 0.771772801 | 0 | 0.006148588 | 0.090705592 | 0.20747109 | 1.595163736 |
| GSTT2 | mRNA | 5.81E-05 | 0.023531419 | 0.398151817 | 0.940624021 | 0 | -0.07309018 | -0.17215541 | -0.15970371 | -0.31459255 |
| GTF2E1 | mRNA | 2.56E-05 | 0.010360156 | 0.959514201 | 0.811046969 | 0 | 0.028168838 | 0.121256487 | 0.022923768 | 0.471761072 |
| GTSE1 | mRNA | 5.77E-05 | 0.023348972 | 0.767248752 | 0.857283574 | 0 | 0.014356183 | 0.550755903 | 0.132924205 | 0.59980414 |
| GZF1 | mRNA | 1.11E-04 | 0.044762582 | 1.493539852 | 0.68376213 | 0 | 0.011596249 | 0.237711738 | 0.011547551 | 0.27562653 |
| GZMH | mRNA | 4.81E-05 | 0.019484883 | 0.579597677 | 0.901086718 | 0 | 0.046366974 | 0.273460584 | 0.146650882 | 2.768292812 |
| H2AFJ | mRNA | 3.12E-05 | 0.012631978 | 2.26965193 | 0.518359709 | 0 | 0.030044831 | 0.035261624 | 0.012266898 | 0.41635516 |
| H3F3B | mRNA | 8.70E-05 | 0.035233154 | 1.996568657 | 0.573119198 | 0 | 0.003071723 | 0.002195675 | 0.050976808 | 0.473560298 |
| HABP4 | mRNA | 4.12E-05 | 0.016682779 | 1.791089727 | 0.616875828 | 0 | -0.04582879 | -0.01917247 | -0.0770065 | -0.48057928 |
| HASPIN | mRNA | 4.99E-05 | 0.020192399 | 0.618298289 | 0.892230918 | 0 | 0.052860927 | 0.515030805 | 0.204167384 | 1.498071394 |
| HCLS1 | mRNA | 1.15E-04 | 0.046775187 | 1.619277845 | 0.65502664 | 0 | 0.035940848 | 0.002696579 | 0.17876839 | 3.252605817 |
| HDAC3 | mRNA | 2.40E-07 | 0.000097147 | 0.353827944 | 0.949606358 | 0 | -0.02932334 | -0.03745381 | -0.03449601 | -0.46208878 |
| HELB | mRNA | 3.70E-05 | 0.015001422 | 0.601566617 | 0.896073639 | 0 | 0.048400412 | 0.127152385 | 0.047455627 | 0.680983258 |
| HERC1 | mRNA | 6.48E-05 | 0.026237324 | 0.486855362 | 0.921769595 | 0 | 0.024130951 | 0.040242908 | 0.047475497 | 0.733150815 |
| HERC5 | mRNA | 1.13E-05 | 0.004566866 | 1.080358796 | 0.781817539 | 0 | 0.01619657 | 0.157985523 | 0.119629004 | 0.771649232 |
| HGSNAT | mRNA | 2.95E-05 | 0.011950526 | 0.296539151 | 0.960678053 | 0 | 0.010410072 | 0.060523766 | 0.022030322 | 0.430102308 |
| HIPK4 | mRNA | 6.42E-05 | 0.025989954 | 1.704653528 | 0.635899706 | 0 | 0.00406967 | 0.240738048 | 0.154695778 | 1.112406029 |
| HIST1H2AG | mRNA | 6.22E-05 | 0.025199034 | 0.252048463 | 0.968779255 | 0 | 0.066170165 | 0.04810443 | 0.202412404 | 0.356453568 |
| HIST1H3C | mRNA | 1.12E-05 | 0.004537011 | 0.985303221 | 0.804808083 | 0 | 0.019585404 | 0.012819633 | 0.054894502 | 2.499184515 |
| HIST1H4E | mRNA | 3.84E-05 | 0.015542129 | 1.295489366 | 0.730204449 | 0 | 0.004914806 | 0.049957711 | 0.058687437 | 0.268002784 |
| HIST1H4H | mRNA | 6.00E-05 | 0.024299593 | 0.04232003 | 0.997713714 | 0 | 0.049318894 | 0.028875132 | 0.032639682 | 0.81523202 |
| HIST2H4B | mRNA | 3.93E-05 | 0.015920292 | 0.833718165 | 0.841386245 | 0 | 0.073389317 | 0.023806749 | 0.026013858 | 0.695415846 |
| HIVEP1 | mRNA | 3.48E-06 | 0.001409819 | 1.4573752 | 0.692142464 | 0 | 0.005653988 | 0.07646963 | 0.12868567 | 1.139107667 |
| HIVEP2 | mRNA | 1.92E-05 | 0.007772723 | 1.07388484 | 0.783381831 | 0 | 0.040218266 | 0.111983473 | 0.071002595 | 0.259273855 |
| HLA-DOB | mRNA | 1.18E-04 | 0.047907782 | 0.405740127 | 0.939054183 | 0 | 0.039410251 | 0.01363441 | 0.095379069 | 1.672970638 |
| HLF | mRNA | 9.97E-05 | 0.040389063 | 0.552715188 | 0.907166528 | 0 | 0.013273274 | 0.18171148 | 0.070285465 | 0.583176152 |
| HMGCL | mRNA | 1.23E-04 | 0.04963558 | 0.686080914 | 0.876472873 | 0 | -0.00433651 | -0.04422275 | -0.05548044 | -0.09904441 |
| HSBP1L1 | mRNA | 6.38E-05 | 0.025846839 | 0.09969007 | 0.991874591 | 0 | -0.04372537 | -0.05041334 | -0.1220087 | -0.43334678 |
| HSD17B12 | mRNA | 9.00E-06 | 0.003645626 | 0.947802769 | 0.813879214 | 0 | -0.01313592 | -0.0460426 | -0.0908343 | -0.23937733 |
| HSPA1B | mRNA | 1.69E-05 | 0.00684011 | 0.785482625 | 0.852936154 | 0 | 0.011964052 | 0.090347753 | 0.062472048 | 0.671492937 |
| HTR1A | mRNA | 8.12E-05 | 0.032881718 | 2.084466208 | 0.555061742 | 0 | 0.009873678 | 0.205641892 | 0.000864506 | 2.687888057 |
| HTRA3 | mRNA | 6.74E-06 | 0.002730073 | 1.295176529 | 0.730278775 | 0 | -0.03170897 | -0.06198236 | -0.19681434 | -0.7462621 |
| HTRA4 | mRNA | 1.75E-05 | 0.007069472 | 0.52835598 | 0.912618996 | 0 | 0.018190118 | 0.196933372 | 0.186978472 | 4.169851927 |
| HUNK | mRNA | 7.34E-05 | 0.029743626 | 0.883954221 | 0.829298197 | 0 | -0.01027723 | -0.18020503 | -0.01141469 | -0.01772644 |
| ICOSLG | mRNA | 5.19E-05 | 0.021014122 | 1.437253508 | 0.696826003 | 0 | -0.05822294 | -0.24832824 | -0.20724464 | -0.26912786 |
| ID3 | mRNA | 1.03E-04 | 0.041714056 | 0.375039658 | 0.945349052 | 0 | 0.010193969 | 0.03299302 | 0.058570653 | 1.058314704 |
| IDH1 | mRNA | 1.01E-05 | 0.004092029 | 1.250894127 | 0.740825432 | 0 | -0.06483983 | -0.11512435 | -0.10853766 | -0.0308645 |
| IDI1 | mRNA | 3.21E-05 | 0.013001138 | 0.441414077 | 0.931560499 | 0 | -0.02391937 | -0.05188482 | -0.05636898 | -0.53135767 |
| IFIH1 | mRNA | 6.80E-05 | 0.027558052 | 0.164935697 | 0.983040888 | 0 | 0.02685918 | 0.055319049 | 0.048326624 | 0.430424322 |
| IFIT1 | mRNA | 1.19E-04 | 0.0483049 | 0.30831299 | 0.958457524 | 0 | 0.121438449 | 0.043120778 | 0.171991313 | 1.007140164 |
| IFIT2 | mRNA | 1.95E-05 | 0.007887878 | 1.214764233 | 0.749465461 | 0 | 0.237147927 | 0.046847954 | 0.217935792 | 2.357759284 |
| IFITM1 | mRNA | 2.69E-05 | 0.010910814 | 0.595431681 | 0.897477385 | 0 | 0.01333093 | 0.059939695 | 0.13900976 | 1.51555813 |
| IFITM10 | mRNA | 2.47E-05 | 0.010018482 | 2.145768735 | 0.542709071 | 0 | -0.02132086 | -0.22548098 | -0.0123757 | -1.3468534 |
| IFNLR1 | mRNA | 4.59E-05 | 0.018600133 | 0.86592515 | 0.833642077 | 0 | 0.000290832 | 0.030956896 | 0.237828369 | 1.500711731 |
| IGSF9 | mRNA | 3.02E-05 | 0.012239598 | 1.383842779 | 0.709326406 | 0 | 0.016781778 | 0.01711954 | 0.116202011 | 1.481890034 |
| IKZF3 | mRNA | 4.56E-05 | 0.018479765 | 0.917743629 | 0.821143889 | 0 | 0.044830868 | 0.323424561 | 0.569558643 | 3.330576914 |
| IL17RA | mRNA | 2.52E-05 | 0.010223676 | 0.201632138 | 0.977325386 | 0 | 0.034103694 | 0.080340735 | 0.098358412 | 0.379318437 |
| IL1R2 | mRNA | 8.98E-06 | 0.003637096 | 0.167525904 | 0.982653182 | 0 | 0.213506381 | 0.251358521 | 0.326199912 | 0.597294707 |
| IL6R | mRNA | 5.47E-05 | 0.022154772 | 1.016629989 | 0.797228078 | 0 | 0.03674637 | 0.082895066 | 0.28504234 | 1.501021383 |
| ILKAP | mRNA | 1.02E-04 | 0.0412795 | 0.311936571 | 0.957768122 | 0 | -0.01817878 | -0.02883057 | -0.02400688 | -0.17814012 |
| IMPG2 | mRNA | 9.56E-05 | 0.038717183 | 0.62241625 | 0.891282054 | 0 | -0.00835823 | -0.16377579 | -0.11630402 | -0.96113954 |
| INKA2 | mRNA | 1.20E-04 | 0.048643731 | 0.690579352 | 0.875417512 | 0 | 0.043085456 | 0.015153105 | 0.079042987 | 0.124223214 |
| INPP5A | mRNA | 2.28E-05 | 0.009236092 | 1.495682618 | 0.683267138 | 0 | 0.011916831 | 0.082961415 | 0.09279308 | 0.083910517 |
| INTS8 | mRNA | 5.76E-05 | 0.023327173 | 1.219804958 | 0.748258289 | 0 | 0.014876845 | 0.028858536 | 0.031357012 | 1.378253283 |
| IRF1 | mRNA | 7.11E-06 | 0.002878874 | 0.559223877 | 0.905700302 | 0 | 0.104094284 | 0.081183073 | 0.201630395 | 0.886528188 |
| IRF2 | mRNA | 6.51E-05 | 0.02636243 | 0.451341876 | 0.929443694 | 0 | 0.016714399 | 0.03224275 | 0.095860876 | 0.416373292 |
| ISG15 | mRNA | 6.88E-05 | 0.027858497 | 0.646561234 | 0.885695304 | 0 | 0.007587889 | 0.0627431 | 0.081716026 | 0.402304337 |
| ISG20 | mRNA | 6.35E-06 | 0.002570847 | 0.107497319 | 0.990922784 | 0 | 0.043546493 | 0.112879626 | 0.149415797 | 0.677668488 |
| ITIH2 | mRNA | 4.18E-05 | 0.016908824 | 1.741384746 | 0.627772551 | 0 | 0.012851022 | 0.383445365 | 0.18384243 | 0.255910539 |
| ITM2B | mRNA | 2.32E-05 | 0.009405744 | 1.622665738 | 0.654261506 | 0 | 0.003570206 | 0.047279489 | 0.037909827 | 1.129927957 |
| ITPR3 | mRNA | 4.78E-06 | 0.00193631 | 0.890163054 | 0.827801019 | 0 | 0.03950224 | 0.329272432 | 0.327464855 | 2.286311729 |
| IWS1 | mRNA | 6.37E-05 | 0.025807033 | 1.363989864 | 0.713996984 | 0 | -0.01873935 | -0.02205732 | -0.09573468 | -0.08109331 |
| JADE2 | mRNA | 1.20E-04 | 0.048719079 | 0.64068763 | 0.887057906 | 0 | 0.00361134 | 0.167640854 | 0.053987162 | 0.423673872 |
| JAG1 | mRNA | 2.93E-06 | 0.001184722 | 1.073600846 | 0.78345046 | 0 | 0.040492748 | 0.228832282 | 0.130883371 | 1.460920261 |
| JAK1 | mRNA | 8.28E-05 | 0.033539949 | 0.280506627 | 0.963651973 | 0 | 0.006665212 | 0.025246469 | 0.04214151 | 0.669015285 |
| KAAG1 | mRNA | 1.11E-04 | 0.044932234 | 0.909135042 | 0.823222772 | 0 | 0.0466835 | 0.088231328 | 0.04382137 | 1.380897841 |
| KAT2A | mRNA | 5.98E-05 | 0.02422614 | 0.255132306 | 0.968233496 | 0 | -0.03997573 | -0.04864333 | -0.06705795 | -0.20804571 |
| KAT2B | mRNA | 4.24E-05 | 0.017168041 | 0.668942779 | 0.880483449 | 0 | 0.010967028 | 0.12662214 | 0.115096686 | 0.944792601 |
| KATNBL1 | mRNA | 9.76E-05 | 0.03951379 | 0.492310626 | 0.920577457 | 0 | 0.009692987 | 0.069712851 | 0.03375758 | 0.831622986 |
| KBTBD7 | mRNA | 1.44E-05 | 0.005811772 | 2.723574461 | 0.436235916 | 0 | 0.003962299 | 0.14272534 | 0.066728694 | 1.382827173 |
| KCNA5 | mRNA | 1.71E-05 | 0.006932992 | 0.283322685 | 0.963133898 | 0 | 0.009762739 | 0.305297449 | 0.402306287 | 3.792642259 |
| KCNJ6 | mRNA | 2.94E-05 | 0.011920671 | 0.337394053 | 0.952848856 | 0 | -0.01823241 | -0.0842298 | -0.03457317 | -0.81713052 |
| KCNK10 | mRNA | 3.10E-05 | 0.012545257 | 2.144113067 | 0.543040067 | 0 | 0.054393811 | 0.060473854 | 0.415849866 | 0.877906919 |
| KCNN2 | mRNA | 1.18E-04 | 0.047913942 | 1.714172974 | 0.63378743 | 0 | -0.00106072 | -0.00508015 | -0.14245098 | -0.71964993 |
| KCTD12 | mRNA | 1.54E-05 | 0.006240167 | 0.307643131 | 0.958584663 | 0 | -0.09860547 | -0.06238505 | -0.03610456 | -0.53549272 |
| KEL | mRNA | 4.41E-05 | 0.017871292 | 2.448918841 | 0.48459336 | 0 | 0.016724862 | 0.19571474 | 0.07162338 | 0.364784992 |
| KIAA1211 | mRNA | 5.57E-05 | 0.022555682 | 1.182507158 | 0.757202803 | 0 | 0.01852485 | 0.27032372 | 0.26838823 | 0.402072733 |
| KIAA1257 | mRNA | 1.09E-07 | 0.00004405 | 1.984364525 | 0.57565824 | 0 | 0.015133111 | 0.497036296 | 0.391401183 | 2.05327444 |
| KIAA1671 | mRNA | 6.64E-05 | 0.026907402 | 2.081314391 | 0.555702229 | 0 | 0.019571348 | 0.061346934 | 0.008126027 | 0.248912666 |
| KIAA1841 | mRNA | 9.91E-05 | 0.040126529 | 0.850501484 | 0.837353443 | 0 | 0.047073676 | 0.044365376 | 0.078664717 | 0.47727403 |
| KIF11 | mRNA | 8.32E-05 | 0.033716236 | 0.759286886 | 0.859178189 | 0 | 0.010593303 | 0.171858771 | 0.090303642 | 0.954036031 |
| KIF14 | mRNA | 8.06E-05 | 0.032624396 | 0.717056249 | 0.869185003 | 0 | 0.033858905 | 0.271524507 | 0.06424675 | 2.219439576 |
| KIF15 | mRNA | 7.49E-05 | 0.030333144 | 1.037787637 | 0.792109523 | 0 | 0.023689048 | 0.10095765 | 0.134927851 | 2.114145073 |
| KIF20A | mRNA | 6.66E-05 | 0.026967586 | 1.121986386 | 0.771769308 | 0 | 0.067073805 | 0.065791676 | 0.152801365 | 3.367789593 |
| KIF4A | mRNA | 5.03E-05 | 0.02038622 | 0.281061549 | 0.96355003 | 0 | 0.036042071 | 0.165314459 | 0.226787526 | 1.969711413 |
| KLF12 | mRNA | 1.63E-05 | 0.006606957 | 0.687811829 | 0.876066917 | 0 | 0.083164201 | 0.110072765 | 0.080020049 | 0.20654186 |
| KLF2 | mRNA | 1.55E-05 | 0.006266231 | 1.799442618 | 0.615056236 | 0 | 0.001685584 | 0.094803438 | 0.105373203 | 1.188569052 |
| KLHDC8A | mRNA | 3.40E-06 | 0.001378069 | 0.418032267 | 0.93649277 | 0 | 0.08054178 | 0.283111634 | 0.254445039 | 0.782607112 |
| KLHL18 | mRNA | 5.18E-05 | 0.020990428 | 0.705699247 | 0.871862617 | 0 | 0.010480965 | 0.06399701 | 0.016062315 | 0.485384983 |
| KLHL24 | mRNA | 2.57E-05 | 0.010388589 | 2.458351801 | 0.482864886 | 0 | 0.003844009 | 0.279601768 | 0.064014365 | 0.137761946 |
| KLK13 | mRNA | 4.01E-05 | 0.016240167 | 0.745757604 | 0.862392097 | 0 | 0.022766313 | 0.040723167 | 0.429489546 | 0.774729741 |
| KMT2B | mRNA | 1.97E-05 | 0.007972704 | 0.373992 | 0.945561151 | 0 | 0.015726359 | 0.027306862 | 0.04381463 | 0.197746517 |
| KMT2D | mRNA | 3.30E-07 | 0.000133637 | 0.985731068 | 0.804704562 | 0 | 0.064706185 | 0.23032864 | 0.047822074 | 0.873065146 |
| KRR1 | mRNA | 1.05E-04 | 0.042335798 | 1.494497462 | 0.683540894 | 0 | -0.01678139 | -0.0558312 | -0.0207385 | -0.18669305 |
| KRT38 | mRNA | 9.10E-05 | 0.036837741 | 0.988223235 | 0.804101556 | 0 | 0.000111095 | 0.082157985 | 0.018223183 | 1.088938867 |
| KSR1 | mRNA | 5.41E-05 | 0.021922093 | 0.215804145 | 0.974998526 | 0 | 0.047997737 | 0.092302007 | 0.101670728 | 0.238313875 |
| LBH | mRNA | 6.86E-05 | 0.027775093 | 0.926570779 | 0.819011392 | 0 | 0.009451488 | 0.107823885 | 0.059659809 | 1.280304393 |
| LBR | mRNA | 3.06E-05 | 0.01239456 | 0.263818189 | 0.966683179 | 0 | 0.029137729 | 0.050617134 | 0.123474792 | 0.701901418 |
| LGI3 | mRNA | 5.50E-05 | 0.022256658 | 0.704352508 | 0.872179721 | 0 | 0.00957181 | 0.028647551 | 0.092371959 | 0.556462346 |
| LHFPL4 | mRNA | 7.91E-07 | 0.000320349 | 1.543208263 | 0.672334383 | 0 | -0.01539058 | -0.19756612 | -0.05405304 | -2.55348822 |
| LHX4 | mRNA | 8.02E-05 | 0.032482229 | 1.935249682 | 0.585954031 | 0 | 0.025556129 | 0.013841219 | 0.048415228 | 1.777442749 |
| LMBR1L | mRNA | 7.88E-05 | 0.031912141 | 1.414221935 | 0.702204443 | 0 | 0.013844754 | 0.078312158 | 0.05253302 | 0.005745858 |
| LNPK | mRNA | 9.39E-06 | 0.003803905 | 2.460557 | 0.482461507 | 0 | -0.07328383 | -0.06642054 | -0.06234801 | -0.16418635 |
| LOXL1 | mRNA | 5.27E-05 | 0.021333523 | 1.423572188 | 0.700018701 | 0 | 0.006610759 | 0.124392098 | 0.43953148 | 0.50764085 |
| LPIN1 | mRNA | 4.17E-05 | 0.016875652 | 0.435217585 | 0.932875024 | 0 | -0.02105747 | -0.05546904 | -0.05523723 | -0.45105168 |
| LRFN1 | mRNA | 4.81E-06 | 0.001947683 | 2.059738632 | 0.560100754 | 0 | 0.027576323 | 0.188261189 | 0.277528436 | 0.307582197 |
| LRGUK | mRNA | 1.17E-04 | 0.047452374 | 0.857635892 | 0.835637294 | 0 | 0.059015065 | 0.135903907 | 0.098504217 | 0.160471117 |
| LRRC6 | mRNA | 1.10E-04 | 0.044349825 | 1.556864387 | 0.669209587 | 0 | 0.005222537 | 0.082122503 | 0.013314737 | 0.373223209 |
| LRRC8C | mRNA | 8.41E-06 | 0.003404417 | 1.905746993 | 0.592197795 | 0 | 0.068946222 | 0.034220756 | 0.045299885 | 1.452456917 |
| LRRK2 | mRNA | 1.30E-06 | 0.000525069 | 0.570949528 | 0.903049398 | 0 | 0.160468703 | 0.335215366 | 0.282523237 | 2.533387159 |
| LRRN4 | mRNA | 1.91E-06 | 0.000773861 | 1.728606797 | 0.63059269 | 0 | -0.01423076 | -0.15245522 | -0.51058218 | -2.07855095 |
| LRWD1 | mRNA | 1.85E-05 | 0.007482703 | 0.30162437 | 0.959722691 | 0 | 0.004783155 | 0.037527355 | 0.064478181 | 0.190462643 |
| LTB | mRNA | 2.51E-05 | 0.010163966 | 0.438435342 | 0.93219306 | 0 | 0.029724063 | 0.343028449 | 0.187625613 | 3.510383471 |
| LUZP6 | mRNA | 1.89E-05 | 0.007650934 | 1.73290919 | 0.629642284 | 0 | 0.001106797 | 0.050546571 | 0.075213104 | 0.512758539 |
| LYST | mRNA | 7.57E-05 | 0.030651123 | 0.359064838 | 0.948562653 | 0 | 0.092447098 | 0.063960906 | 0.199427561 | 0.675726024 |
| LZIC | mRNA | 8.35E-05 | 0.033834234 | 1.555250105 | 0.669578572 | 0 | -0.02592433 | -0.00737102 | -0.01818828 | -0.01910144 |
| MAGEB2 | mRNA | 6.19E-05 | 0.025058289 | 0.060495753 | 0.996113686 | 0 | -0.02638359 | -0.03655605 | -0.10177297 | -2.82920991 |
| MAGEE1 | mRNA | 4.92E-05 | 0.019921809 | 1.468319234 | 0.689601297 | 0 | 0.03412478 | 0.057408467 | 0.026474127 | 0.19858978 |
| MAMDC2 | mRNA | 4.28E-05 | 0.017322055 | 0.423288538 | 0.935390705 | 0 | 0.066231983 | 0.289865163 | 0.243133004 | 0.439856028 |
| MAML2 | mRNA | 9.90E-05 | 0.040080561 | 1.20886575 | 0.750878734 | 0 | 0.004353373 | 0.049036617 | 0.017210133 | 0.699020491 |
| MAN1A2 | mRNA | 2.49E-05 | 0.010073453 | 1.160664288 | 0.762453259 | 0 | -0.00696357 | -0.05181066 | -0.03517511 | -0.08629085 |
| MAN2B2 | mRNA | 3.97E-05 | 0.016098474 | 1.675219889 | 0.642456961 | 0 | 0.014905419 | 0.046428647 | 0.062904606 | 0.373062908 |
| MANSC1 | mRNA | 5.78E-06 | 0.002339589 | 1.391195028 | 0.707599958 | 0 | 0.015739837 | 0.392616938 | 0.220979362 | 0.466508558 |
| MAOB | mRNA | 2.85E-05 | 0.011561464 | 2.104371407 | 0.551028904 | 0 | 0.015275957 | 0.325182491 | 0.319462522 | 0.047549914 |
| MAP3K14 | mRNA | 2.33E-05 | 0.009429438 | 1.540910011 | 0.672861012 | 0 | 0.001350522 | 0.121271024 | 0.103858577 | 0.299279908 |
| MAP3K5 | mRNA | 1.02E-04 | 0.04135627 | 2.247206552 | 0.522710101 | 0 | 0.003795187 | 0.06771567 | 0.019807221 | 0.050894711 |
| MAP3K6 | mRNA | 1.99E-05 | 0.008058004 | 1.396497838 | 0.706355863 | 0 | -0.07738266 | -0.01003341 | -0.06076759 | -0.62394468 |
| MAP7D1 | mRNA | 6.30E-05 | 0.025516065 | 1.445667956 | 0.69486568 | 0 | 0.001752697 | 0.040611694 | 0.040722504 | 0.063978253 |
| MBD2 | mRNA | 7.60E-05 | 0.030775756 | 0.321979596 | 0.955843072 | 0 | 0.005302412 | 0.013864806 | 0.023776202 | 0.123554613 |
| MBOAT1 | mRNA | 5.85E-05 | 0.023677377 | 2.471479671 | 0.480467402 | 0 | 0.031901007 | 0.027191477 | 0.156848148 | 1.515820139 |
| MBTD1 | mRNA | 8.75E-05 | 0.035441665 | 0.368209077 | 0.946728547 | 0 | 0.005323391 | 0.030590061 | 0.031913699 | 0.884559212 |
| MCF2L2 | mRNA | 2.15E-05 | 0.008699176 | 1.068696368 | 0.784635766 | 0 | 0.035083124 | 0.08705995 | 0.317569986 | 0.449265519 |
| MED12 | mRNA | 3.33E-05 | 0.013482135 | 1.04627971 | 0.790055575 | 0 | 0.024900713 | 0.036537666 | 0.088695608 | 0.640127131 |
| MED13L | mRNA | 1.33E-05 | 0.005372477 | 0.960930487 | 0.810704409 | 0 | 0.021811302 | 0.066288522 | 0.083683698 | 0.629106611 |
| MEGF9 | mRNA | 2.83E-07 | 0.000114681 | 1.979256591 | 0.576723221 | 0 | 0.096467667 | 0.147453867 | 0.161954188 | 0.880667743 |
| MEI1 | mRNA | 5.22E-05 | 0.021130699 | 0.044334253 | 0.99755004 | 0 | 0.017575622 | 0.181552392 | 0.215419551 | 1.821029859 |
| MELK | mRNA | 5.59E-05 | 0.022646669 | 1.865875395 | 0.600705681 | 0 | 0.051188519 | 0.103658546 | 0.027278816 | 1.525488038 |
| METAP2 | mRNA | 7.67E-05 | 0.031061037 | 1.144317193 | 0.766387921 | 0 | -0.00210446 | -0.05880244 | -0.03843705 | -0.29675503 |
| METTL22 | mRNA | 3.21E-05 | 0.012983604 | 2.0898266 | 0.553973652 | 0 | 0.002744524 | 0.053964442 | 0.015292462 | 0.577109321 |
| MFSD10 | mRNA | 4.15E-05 | 0.016789404 | 1.139851964 | 0.767463401 | 0 | -0.04522792 | -0.03579733 | -0.06425702 | -0.02670581 |
| MGAM | mRNA | 7.72E-07 | 0.000312767 | 1.2848088 | 0.7327435 | 0 | 0.258815668 | 0.324234099 | 0.293812222 | 6.715433379 |
| MGAT5 | mRNA | 6.04E-05 | 0.024480618 | 0.816999857 | 0.84539672 | 0 | -0.04695581 | -0.02190459 | -0.07263576 | -1.05075873 |
| MIA3 | mRNA | 9.66E-05 | 0.039116672 | 0.700611404 | 0.873060131 | 0 | -0.0175577 | -0.03157826 | -0.03811512 | -0.03179334 |
| MIEN1 | mRNA | 9.20E-05 | 0.037278457 | 0.024426545 | 0.998992067 | 0 | 0.008515013 | 0.021545325 | 0.01119692 | 0.420219167 |
| MKRN1 | mRNA | 6.95E-06 | 0.002813478 | 1.365659703 | 0.713603655 | 0 | 0.006782145 | 0.053021469 | 0.064915132 | 0.227693227 |
| MLLT11 | mRNA | 1.83E-05 | 0.007410198 | 1.417739855 | 0.70138173 | 0 | 0.017646964 | 0.054968852 | 0.028138394 | 0.693437573 |
| MLLT6 | mRNA | 9.20E-07 | 0.000372477 | 2.458491686 | 0.48283929 | 0 | 0.008544873 | 0.057965586 | 0.079568351 | 0.272980239 |
| MLXIPL | mRNA | 1.35E-06 | 0.000547342 | 2.288717769 | 0.514685728 | 0 | -0.00973998 | -0.34924211 | -0.24797274 | -3.07567204 |
| MMP15 | mRNA | 2.50E-05 | 0.010119894 | 1.091386138 | 0.779153901 | 0 | -0.02551052 | -0.11145301 | -0.11224061 | -0.42738175 |
| MMP25 | mRNA | 1.88E-07 | 0.000076296 | 1.579393077 | 0.664071275 | 0 | 0.071177758 | 0.138806181 | 0.2386996 | 3.25367522 |
| MNDA | mRNA | 1.90E-05 | 0.007714909 | 0.76343345 | 0.858191759 | 0 | 0.103081571 | 0.151688812 | 0.20639447 | 3.665362254 |
| MPP3 | mRNA | 8.60E-05 | 0.034837456 | 1.062423512 | 0.78615205 | 0 | -0.01338764 | -0.07877916 | -0.06684711 | -0.13861271 |
| MRAP2 | mRNA | 4.51E-05 | 0.018256564 | 1.070621459 | 0.78417049 | 0 | -0.0034194 | -0.10059809 | -0.44150455 | -0.45370635 |
| MSRB1 | mRNA | 3.87E-05 | 0.015655862 | 0.260453784 | 0.967285959 | 0 | 0.046355545 | 0.045411418 | 0.124836614 | 0.323725136 |
| MT1A | mRNA | 8.87E-05 | 0.035910341 | 1.160700403 | 0.762444571 | 0 | -0.02889557 | -0.05515784 | -0.02037664 | -1.04341379 |
| MT1F | mRNA | 2.41E-05 | 0.009773008 | 0.090777666 | 0.992920693 | 0 | -0.03308262 | -0.05364126 | -0.0660948 | -0.72485406 |
| MT1M | mRNA | 6.01E-05 | 0.024342243 | 0.437981862 | 0.932289255 | 0 | -0.07556676 | -0.04046134 | -0.09028964 | -0.54398651 |
| MT1X | mRNA | 9.34E-05 | 0.037817743 | 0.343270684 | 0.951695175 | 0 | -0.01942355 | -0.05649606 | -0.03786571 | -0.30489679 |
| MTNR1B | mRNA | 2.40E-05 | 0.00972941 | 0.148989562 | 0.985370692 | 0 | 0.018204525 | 0.206989833 | 0.564680222 | 2.003900921 |
| MTRNR2L8 | mRNA | 7.98E-05 | 0.032313051 | 0.74906181 | 0.86160784 | 0 | -0.01812623 | -0.0306152 | -0.07074785 | -0.00840031 |
| MTURN | mRNA | 5.87E-07 | 0.000237892 | 1.545719593 | 0.671759173 | 0 | 0.041784597 | 0.103177636 | 0.138577587 | 0.819736588 |
| MVD | mRNA | 9.27E-06 | 0.003754621 | 1.427555289 | 0.69908852 | 0 | -0.05147498 | -0.06807292 | -0.10660741 | -1.65536323 |
| MX2 | mRNA | 7.54E-05 | 0.030543077 | 1.48780501 | 0.685087774 | 0 | 0.053338786 | 0.01050704 | 0.238874078 | 1.915206955 |
| MXD3 | mRNA | 1.27E-05 | 0.00515354 | 2.73448023 | 0.434399507 | 0 | 0.01487197 | 0.071872884 | 0.312273671 | 0.05729177 |
| MXRA5 | mRNA | 1.02E-04 | 0.041423562 | 0.574089171 | 0.902337588 | 0 | -0.09021802 | -0.11539011 | -0.41260376 | -0.26038592 |
| MYBPC3 | mRNA | 4.56E-05 | 0.018451806 | 1.540846268 | 0.672875621 | 0 | 0.011514051 | 0.0313589 | 0.316211912 | 0.06002896 |
| MYDGF | mRNA | 7.50E-05 | 0.030381481 | 1.474601004 | 0.688144689 | 0 | -0.05976746 | -0.01999231 | -0.05472382 | -0.02070806 |
| MYH9 | mRNA | 4.30E-05 | 0.017428206 | 2.946902588 | 0.399884411 | 0 | 0.039153793 | 0.007973422 | 0.088372893 | 0.111785479 |
| MYLIP | mRNA | 8.35E-06 | 0.00338167 | 1.852997442 | 0.603470558 | 0 | 0.062430765 | 0.181269373 | 0.100544535 | 0.982553871 |
| MYO5B | mRNA | 8.65E-05 | 0.035024642 | 1.180210865 | 0.757754371 | 0 | 0.058193506 | 0.049174195 | 0.013136147 | 1.046258761 |
| MYO5C | mRNA | 7.81E-05 | 0.031650081 | 1.299744377 | 0.729193777 | 0 | 0.023742167 | 0.028024465 | 0.155517492 | 2.701500427 |
| NAALADL1 | mRNA | 4.98E-05 | 0.020158279 | 1.359546673 | 0.715043997 | 0 | 0.013661482 | 0.220854121 | 0.015416559 | 0.754906872 |
| NAP1L2 | mRNA | 2.92E-05 | 0.011834424 | 0.834852197 | 0.841113957 | 0 | 0.022000349 | 0.068135164 | 0.607171861 | 0.224703365 |
| NAT9 | mRNA | 6.93E-05 | 0.028076012 | 1.301127374 | 0.728865388 | 0 | -0.05466542 | -0.02960769 | -0.03834754 | -0.0650261 |
| NCAPD2 | mRNA | 4.05E-05 | 0.016383755 | 0.846903942 | 0.838218411 | 0 | 0.014010093 | 0.133028284 | 0.059908559 | 0.203768384 |
| NCF4 | mRNA | 2.76E-05 | 0.011193726 | 0.181090466 | 0.980582621 | 0 | 0.071397369 | 0.038309857 | 0.26677583 | 2.66932062 |
| NDC80 | mRNA | 3.73E-05 | 0.015096674 | 1.890159578 | 0.595514381 | 0 | 0.064336021 | 0.097175115 | 0.050681309 | 1.280010118 |
| NDST1 | mRNA | 6.11E-05 | 0.024747418 | 0.065430255 | 0.995635064 | 0 | 0.005195065 | 0.050279607 | 0.050521414 | 0.726189451 |
| NEMF | mRNA | 2.37E-05 | 0.009599564 | 0.4451419 | 0.930767183 | 0 | -0.00862421 | -0.07275018 | -0.00946589 | -0.86411028 |
| NEU3 | mRNA | 3.57E-05 | 0.014442233 | 1.633078421 | 0.651912984 | 0 | 0.005916654 | 0.047009244 | 0.161833183 | 0.718828157 |
| NFAM1 | mRNA | 2.43E-05 | 0.009856412 | 0.249644387 | 0.969202987 | 0 | 0.044889373 | 0.232999046 | 0.224188505 | 4.109546208 |
| NFIL3 | mRNA | 2.95E-06 | 0.001193726 | 2.495960372 | 0.476021577 | 0 | 0.058046075 | 0.090542657 | 0.035968378 | 0.661882986 |
| NHLRC3 | mRNA | 8.78E-06 | 0.003557483 | 0.638673694 | 0.8875246 | 0 | -0.06585336 | -0.06287291 | -0.03060019 | -0.39913234 |
| NHSL2 | mRNA | 9.22E-07 | 0.000373425 | 2.605970593 | 0.456443786 | 0 | 0.094380051 | 0.185833932 | 0.688638836 | 1.913401903 |
| NIPSNAP3B | mRNA | 3.81E-05 | 0.015410862 | 1.337012785 | 0.720363348 | 0 | -0.05436284 | -0.21700571 | -0.14355279 | -0.13562283 |
| NKG7 | mRNA | 5.48E-05 | 0.022176097 | 0.447980305 | 0.930161905 | 0 | 0.048272554 | 0.090816083 | 0.179950009 | 4.6139458 |
| NKPD1 | mRNA | 5.28E-05 | 0.021383281 | 0.265436887 | 0.966392155 | 0 | -0.01010055 | -0.09340535 | -0.19111388 | -0.31695051 |
| NLRP6 | mRNA | 2.52E-06 | 0.001020757 | 0.667987223 | 0.880706575 | 0 | 0.044794325 | 0.52921652 | 0.587488573 | 4.414918137 |
| NME4 | mRNA | 2.85E-05 | 0.011543456 | 0.953425317 | 0.812519581 | 0 | -0.01047262 | -0.04287015 | -0.0959733 | -0.23630063 |
| NME8 | mRNA | 8.08E-05 | 0.032738603 | 0.765009454 | 0.857816676 | 0 | 0.064933196 | 0.035213541 | 0.30023281 | 2.601291077 |
| NODAL | mRNA | 1.76E-05 | 0.007117809 | 2.848601221 | 0.415561656 | 0 | 0.052842014 | 0.237058597 | 0.008903027 | 1.322527219 |
| NOG | mRNA | 6.21E-05 | 0.025143589 | 0.077922669 | 0.994348226 | 0 | 0.029384213 | 0.056102353 | 0.407785246 | 3.236226086 |
| NOTUM | mRNA | 8.09E-06 | 0.003275993 | 0.966245654 | 0.809418732 | 0 | 0.010730541 | 0.165469381 | 0.014245389 | 2.360536811 |
| NPAS1 | mRNA | 6.58E-05 | 0.026646764 | 1.677733818 | 0.64189537 | 0 | 0.014853189 | 0.264182627 | 0.01061179 | 0.705446823 |
| NPC1 | mRNA | 1.13E-04 | 0.045756801 | 0.797700636 | 0.850016932 | 0 | 0.015447926 | 0.051260683 | 0.069447702 | 0.043740029 |
| NPTXR | mRNA | 1.19E-04 | 0.048294001 | 1.17704312 | 0.75851542 | 0 | 0.003539774 | 0.026556481 | 0.136211836 | 0.283166259 |
| NQO2 | mRNA | 5.09E-05 | 0.020607526 | 1.530855484 | 0.675167428 | 0 | -0.00902117 | -0.14500771 | -0.05446176 | -0.49506954 |
| NRBF2 | mRNA | 4.99E-05 | 0.020208037 | 0.39673555 | 0.940916025 | 0 | 0.028857781 | 0.013952303 | 0.145974638 | 0.332777528 |
| NSRP1 | mRNA | 6.40E-05 | 0.02591271 | 0.320989373 | 0.9560338 | 0 | -0.0134299 | -0.0555864 | -0.02276809 | -0.88214791 |
| NUAK2 | mRNA | 3.51E-09 | 0.00000142 | 1.69460265 | 0.638134416 | 0 | 0.077852057 | 0.146773356 | 0.26252399 | 1.505331166 |
| NUMB | mRNA | 1.46E-05 | 0.0059274 | 0.2543476 | 0.968372603 | 0 | 0.055805906 | 0.037117844 | 0.078310803 | 0.833794915 |
| NUTM2A | mRNA | 6.07E-06 | 0.002456639 | 0.728036158 | 0.86659058 | 0 | 0.016307105 | 0.053032644 | 0.213474672 | 1.415621697 |
| OMG | mRNA | 4.77E-05 | 0.019301014 | 0.950043549 | 0.813337377 | 0 | 0.034822221 | 0.052118988 | 0.910763405 | 1.203498362 |
| OR2W3 | mRNA | 4.68E-05 | 0.018943702 | 0.467400177 | 0.925992806 | 0 | 0.036427569 | 0.161248282 | 0.470131872 | 2.903031683 |
| OR7C1 | mRNA | 1.69E-05 | 0.006832528 | 0.087132294 | 0.993335562 | 0 | 0.050470668 | 0.171772407 | 0.118713845 | 1.568853473 |
| ORC6 | mRNA | 5.74E-05 | 0.023254194 | 0.810448129 | 0.846966381 | 0 | 0.030553767 | 0.191520764 | 0.089635586 | 1.080014769 |
| OSM | mRNA | 3.26E-05 | 0.013191641 | 0.150228299 | 0.985193322 | 0 | 0.187328022 | 0.074478424 | 0.173356572 | 2.886474259 |
| OXCT2 | mRNA | 5.89E-05 | 0.023854611 | 1.669077516 | 0.64383031 | 0 | -0.03786518 | -0.27812134 | -0.12159493 | -0.13902972 |
| P4HB | mRNA | 2.62E-05 | 0.010612738 | 0.06059477 | 0.996104256 | 0 | -0.02533713 | -0.02908748 | -0.03967171 | -0.5810824 |
| PADI2 | mRNA | 6.84E-06 | 0.002768932 | 0.702315563 | 0.87265917 | 0 | 0.076466084 | 0.264379075 | 0.361166769 | 4.9177819 |
| PADI4 | mRNA | 4.43E-06 | 0.001793669 | 1.526303013 | 0.676213049 | 0 | 0.04049212 | 0.271263111 | 0.788314568 | 6.161057972 |
| PAG1 | mRNA | 7.99E-06 | 0.003237608 | 0.155392916 | 0.984447155 | 0 | 0.053072999 | 0.214966167 | 0.145024781 | 0.592644593 |
| PARP14 | mRNA | 7.22E-05 | 0.029258364 | 0.948338843 | 0.813749591 | 0 | 0.006997795 | 0.037054885 | 0.045664943 | 0.797371124 |
| PBDC1 | mRNA | 1.13E-04 | 0.045698512 | 0.33141769 | 0.954015307 | 0 | -0.03183379 | -0.05208066 | -0.08048807 | -0.17424748 |
| PCBP1 | mRNA | 8.46E-05 | 0.034262629 | 2.627930229 | 0.452614044 | 0 | 0.002652645 | 0.006968404 | 0.031039417 | 0.257272503 |
| PCED1B | mRNA | 5.23E-05 | 0.021182827 | 1.240662796 | 0.743269114 | 0 | 0.002815838 | 0.049935747 | 0.087350183 | 0.07432748 |
| PCNX2 | mRNA | 8.76E-06 | 0.003547531 | 1.10670573 | 0.775455545 | 0 | 0.035882534 | 0.090227786 | 0.057440257 | 0.098399691 |
| PDE7A | mRNA | 3.20E-05 | 0.012972704 | 1.073083863 | 0.783575394 | 0 | 0.003296707 | 0.080996713 | 0.061717793 | 0.742395257 |
| PDIA3 | mRNA | 6.87E-05 | 0.027840963 | 0.665730014 | 0.881233431 | 0 | -0.02080181 | -0.01939211 | -0.03702075 | -0.05246101 |
| PDXK | mRNA | 1.69E-05 | 0.00685954 | 2.533337443 | 0.469296377 | 0 | -0.01040445 | -0.02888844 | -0.06954568 | -0.89835712 |
| PDZK1IP1 | mRNA | 6.97E-05 | 0.028212966 | 1.483349968 | 0.686118446 | 0 | 0.006144746 | 0.120770653 | 0.097246892 | 5.098552716 |
| PELI1 | mRNA | 6.47E-05 | 0.026201308 | 0.738688496 | 0.864068465 | 0 | 0.110835016 | 0.029414419 | 0.141030931 | 0.713731436 |
| PHC3 | mRNA | 9.29E-05 | 0.037638139 | 0.064413638 | 0.995735102 | 0 | 0.007892086 | 0.023955873 | 0.016165169 | 1.193829766 |
| PHF12 | mRNA | 9.22E-05 | 0.037324898 | 0.537872562 | 0.910495583 | 0 | 0.016238301 | 0.070285695 | 0.194667777 | 0.123004186 |
| PHGDH | mRNA | 2.76E-05 | 0.011192304 | 1.603096123 | 0.658687999 | 0 | 0.055835288 | 0.153115906 | 0.046020027 | 0.782342956 |
| PHOSPHO1 | mRNA | 5.84E-05 | 0.023653682 | 0.771867113 | 0.856183528 | 0 | 0.023162571 | 0.053397133 | 0.105918827 | 4.396195307 |
| PI16 | mRNA | 1.11E-04 | 0.045071084 | 0.990107595 | 0.80364561 | 0 | 0.028920588 | 0.008171351 | 0.369909016 | 1.474395847 |
| PIF1 | mRNA | 6.66E-06 | 0.002695479 | 2.640025982 | 0.450515645 | 0 | 0.029637909 | 0.273354618 | 0.240631253 | 0.689891857 |
| PIK3CD | mRNA | 1.78E-06 | 0.000721733 | 1.636340288 | 0.65117826 | 0 | 0.046802415 | 0.091259245 | 0.217309431 | 0.68701395 |
| PKNOX2 | mRNA | 4.56E-06 | 0.001847692 | 2.537856153 | 0.468488459 | 0 | 0.03171346 | 0.817318328 | 0.157337112 | 0.829447339 |
| PLCG2 | mRNA | 3.36E-05 | 0.013624775 | 0.538267119 | 0.910407357 | 0 | 0.026954673 | 0.203409319 | 0.272044378 | 4.053325475 |
| PLEKHA6 | mRNA | 1.03E-04 | 0.041732537 | 0.839760749 | 0.83993503 | 0 | 0.019749363 | 0.121889163 | 0.048305499 | 1.418301284 |
| PLEKHF1 | mRNA | 9.31E-05 | 0.037704009 | 0.5486179 | 0.908087559 | 0 | 0.004040029 | 0.065491077 | 0.07617336 | 0.301976224 |
| PLEKHF2 | mRNA | 3.90E-05 | 0.015775756 | 1.007457344 | 0.799447506 | 0 | 0.01368201 | 0.116333876 | 0.035940671 | 0.632240885 |
| PLEKHG1 | mRNA | 1.65E-05 | 0.006690835 | 1.547708783 | 0.671303738 | 0 | -0.03651243 | -0.26952148 | -0.1933422 | -0.15641349 |
| PLEKHG3 | mRNA | 1.44E-05 | 0.005818406 | 0.946362 | 0.814227585 | 0 | 0.035558415 | 0.112506372 | 0.208059754 | 0.061964111 |
| PLEKHG4B | mRNA | 5.20E-05 | 0.021067198 | 0.194829947 | 0.978419574 | 0 | -0.00699487 | -0.05395548 | -0.10100293 | -1.5172794 |
| PLEKHM1 | mRNA | 3.46E-05 | 0.014026159 | 2.114041289 | 0.549077342 | 0 | 0.016810781 | 0.039533188 | 0.040792594 | 0.703422373 |
| PLIN4 | mRNA | 8.04E-06 | 0.003256564 | 1.622109899 | 0.654387004 | 0 | 0.000117149 | 0.24678965 | 0.242190251 | 1.131706482 |
| PLK1 | mRNA | 2.30E-05 | 0.009314283 | 0.21330807 | 0.975412856 | 0 | 0.018692327 | 0.179943832 | 0.263345827 | 0.912333472 |
| PLPP3 | mRNA | 4.64E-05 | 0.018779263 | 2.18633401 | 0.534645159 | 0 | 0.009350375 | 0.13570949 | 0.091493616 | 0.272473848 |
| PLXNC1 | mRNA | 6.45E-05 | 0.026129751 | 0.173028464 | 0.981821281 | 0 | 0.074420045 | 0.032539957 | 0.157701496 | 1.019447704 |
| PNMA2 | mRNA | 5.42E-06 | 0.002196948 | 2.642727248 | 0.450048098 | 0 | 0.031045976 | 0.077321133 | 0.575791095 | 0.955727935 |
| PNPLA3 | mRNA | 5.51E-06 | 0.002230121 | 0.248568627 | 0.969392102 | 0 | -0.07373928 | -0.17551202 | -0.15584454 | -1.32252989 |
| POLQ | mRNA | 1.33E-07 | 5.40E-05 | 0.282183783 | 0.963343648 | 0 | 0.023419692 | 0.281635311 | 0.329217147 | 1.656550511 |
| POLR2A | mRNA | 1.42E-06 | 0.000574827 | 1.413645082 | 0.702339388 | 0 | 0.020118878 | 0.131301908 | 0.037745366 | 0.312819826 |
| POU2AF1 | mRNA | 1.00E-04 | 0.04056298 | 0.404849362 | 0.939238918 | 0 | 0.018088078 | 0.592385756 | 0.232738292 | 2.469059977 |
| PPFIA1 | mRNA | 7.66E-05 | 0.031033078 | 0.353916018 | 0.949588846 | 0 | 0.010319702 | 0.081528467 | 0.044261121 | 0.132651442 |
| PPFIA4 | mRNA | 2.06E-05 | 0.008337125 | 0.531575406 | 0.911901645 | 0 | -0.02448571 | -0.40034184 | -0.19420989 | -0.6598753 |
| PPIG | mRNA | 4.83E-05 | 0.019551702 | 0.916478514 | 0.821449453 | 0 | -0.02030324 | -0.03541619 | -0.04192182 | -0.12186884 |
| PPM1F | mRNA | 5.17E-06 | 0.002092219 | 2.735231724 | 0.434273197 | 0 | 0.003112537 | 0.048963342 | 0.061318831 | 0.720000408 |
| PPM1L | mRNA | 1.03E-06 | 0.00041797 | 1.405358525 | 0.704279145 | 0 | -0.11485634 | -0.18258307 | -0.25399064 | -0.77089167 |
| PPP1R11 | mRNA | 2.54E-06 | 0.001027865 | 0.592136675 | 0.898230119 | 0 | 0.024985081 | 0.021548232 | 0.053028512 | 0.520454069 |
| PPP1R36 | mRNA | 1.01E-04 | 0.041001327 | 0.162108222 | 0.983461214 | 0 | 0.023794718 | 0.112387696 | 0.240244548 | 1.719231215 |
| PPP1R9B | mRNA | 8.93E-06 | 0.003617667 | 0.961819384 | 0.810489404 | 0 | 0.004776123 | 0.04133178 | 0.065638745 | 0.273060282 |
| PRADC1 | mRNA | 6.09E-05 | 0.024653114 | 0.997267768 | 0.801913077 | 0 | -0.05645372 | -0.03595382 | -0.052607 | -0.16086928 |
| PREX1 | mRNA | 1.86E-05 | 0.007546204 | 0.336021841 | 0.953117293 | 0 | 0.08706442 | 0.155599082 | 0.186433481 | 1.744103903 |
| PRICKLE2 | mRNA | 3.53E-05 | 0.014294854 | 0.539944285 | 0.910032161 | 0 | -0.06228637 | -0.14974316 | -0.11221757 | -0.78890951 |
| PRLR | mRNA | 3.88E-05 | 0.015732632 | 1.711375187 | 0.6344078 | 0 | 0.093161903 | 0.107399054 | 0.005311353 | 1.21822105 |
| PRND | mRNA | 4.38E-07 | 0.000177235 | 2.614507259 | 0.454951912 | 0 | -0.00028635 | -0.40361448 | -0.62886286 | -2.2757654 |
| PRPH | mRNA | 1.03E-05 | 0.004173538 | 1.56950972 | 0.666322874 | 0 | -0.01723258 | -0.12809067 | -0.02611591 | -3.25136089 |
| PRRT1 | mRNA | 5.58E-05 | 0.022606862 | 0.098485119 | 0.992018594 | 0 | 0.033677439 | 0.077624762 | 0.134085118 | 0.210199753 |
| PRSS8 | mRNA | 1.52E-05 | 0.006164819 | 0.633738062 | 0.888667217 | 0 | 0.038360384 | 0.269285114 | 0.21212158 | 2.19697578 |
| PSAPL1 | mRNA | 2.70E-06 | 0.001095157 | 1.926240207 | 0.587856055 | 0 | -0.08212582 | -0.15537705 | -0.24367119 | -1.01789535 |
| PSPC1 | mRNA | 9.86E-05 | 0.039935077 | 1.213176706 | 0.749845759 | 0 | 0.007370634 | 0.001583148 | 0.019221287 | 0.438379782 |
| PSTPIP1 | mRNA | 1.28E-05 | 0.005163966 | 1.001338547 | 0.800928068 | 0 | 0.016090793 | 0.02356605 | 0.270246336 | 3.2374921 |
| PTP4A1 | mRNA | 3.91E-05 | 0.015816984 | 2.573102068 | 0.462224627 | 0 | 0.016686096 | 0.068659353 | 0.029819642 | 0.193683148 |
| PTPRJ | mRNA | 4.44E-05 | 0.018001138 | 0.281928477 | 0.963390625 | 0 | 0.043773528 | 0.125931287 | 0.857515401 | 0.957421276 |
| QPRT | mRNA | 7.92E-07 | 0.000320823 | 0.431428786 | 0.93367619 | 0 | -0.04488217 | -0.14653478 | -0.16423297 | -1.81518373 |
| R3HDM4 | mRNA | 6.01E-05 | 0.024339399 | 1.227069505 | 0.746519515 | 0 | 0.015398426 | 0.074462279 | 0.086009946 | 0.113542271 |
| RAB15 | mRNA | 9.64E-05 | 0.039034215 | 2.483511879 | 0.478278227 | 0 | 0.01447144 | 0.134191578 | 0.017857397 | 0.114704994 |
| RAB3D | mRNA | 4.57E-06 | 0.001851484 | 2.14946947 | 0.54196976 | 0 | 0.037591526 | 0.108197252 | 0.230170465 | 1.190846525 |
| RAB41 | mRNA | 5.27E-05 | 0.021355322 | 0.318017002 | 0.956605109 | 0 | -0.01613888 | -0.1563138 | -0.19570827 | -2.31400893 |
| RABIF | mRNA | 1.20E-04 | 0.048474079 | 1.344516634 | 0.718590274 | 0 | 0.006239155 | 0.006045984 | 0.284764585 | 0.621782482 |
| RAD21 | mRNA | 1.64E-05 | 0.006633021 | 1.6405606 | 0.650228342 | 0 | 0.011095728 | 0.062655088 | 0.021621725 | 0.717431865 |
| RALGAPA2 | mRNA | 1.04E-05 | 0.004222823 | 1.397803214 | 0.706049751 | 0 | 0.067167482 | 0.12583908 | 0.092151055 | 0.172515293 |
| RASAL3 | mRNA | 3.51E-05 | 0.014212871 | 0.916582111 | 0.821424432 | 0 | 0.001469747 | 0.140171479 | 0.167505038 | 3.333416637 |
| RASGEF1C | mRNA | 4.14E-05 | 0.016751967 | 0.589718742 | 0.898781946 | 0 | -0.00941399 | -0.06587509 | -0.14385974 | -0.87453589 |
| RASSF2 | mRNA | 8.41E-06 | 0.003406312 | 0.26834916 | 0.965866918 | 0 | 0.089360911 | 0.131246825 | 0.185504826 | 0.482784737 |
| RBL2 | mRNA | 8.25E-05 | 0.033396361 | 0.069646452 | 0.995212473 | 0 | 0.007039652 | 0.050735616 | 0.034929103 | 0.490537488 |
| RBM44 | mRNA | 6.15E-05 | 0.0249147 | 1.377146121 | 0.710900442 | 0 | -0.09623066 | -0.3791856 | -0.05525901 | -0.08185143 |
| RELB | mRNA | 3.05E-05 | 0.012365179 | 1.652179006 | 0.647617291 | 0 | 0.005748301 | 0.060461401 | 0.037513944 | 0.129426941 |
| RFLNB | mRNA | 5.80E-05 | 0.0234864 | 0.690862197 | 0.875351119 | 0 | 0.007931599 | 0.096120618 | 0.115843151 | 0.183061065 |
| RGS9BP | mRNA | 3.51E-05 | 0.01420055 | 0.715753654 | 0.869492424 | 0 | -0.09702178 | -0.14422193 | -0.20563886 | -0.43311702 |
| RIPK2 | mRNA | 7.93E-05 | 0.032132026 | 1.336255278 | 0.720542432 | 0 | 0.023517214 | 0.013521058 | 0.059726491 | 0.558177248 |
| RIT1 | mRNA | 1.10E-04 | 0.044722775 | 0.33128386 | 0.954041348 | 0 | 0.004938305 | 0.04266593 | 0.036392556 | 0.432978942 |
| RNF141 | mRNA | 7.23E-05 | 0.029280163 | 1.491073104 | 0.684332181 | 0 | 0.015574038 | 0.045742917 | 0.014666662 | 0.835041826 |
| RNF167 | mRNA | 8.54E-05 | 0.03460146 | 0.124718202 | 0.988714462 | 0 | 0.030372947 | 0.035978873 | 0.021354513 | 0.489045291 |
| RNF214 | mRNA | 9.16E-05 | 0.037090797 | 0.967447029 | 0.809128116 | 0 | -0.06024994 | -0.03884729 | -0.11834776 | -0.11697011 |
| RNF43 | mRNA | 4.56E-05 | 0.018467918 | 0.704210934 | 0.872213051 | 0 | 0.035530546 | 0.140047981 | 0.138723 | 0.202737095 |
| RNF44 | mRNA | 6.76E-06 | 0.002736234 | 0.410842641 | 0.937993663 | 0 | 0.009522006 | 0.101657053 | 0.078655192 | 0.677764567 |
| RPS15 | mRNA | 4.88E-05 | 0.019773955 | 1.338172128 | 0.720089298 | 0 | -0.01047554 | -0.05176091 | -0.01056596 | -0.04585334 |
| RPS6KA5 | mRNA | 2.69E-05 | 0.010876694 | 0.989429741 | 0.803809626 | 0 | 0.071624277 | 0.165003918 | 0.102933731 | 0.101528532 |
| RRBP1 | mRNA | 9.51E-05 | 0.038523837 | 1.580386059 | 0.663845281 | 0 | -0.05188422 | -0.00449531 | -0.03459367 | -0.8257169 |
| RSPH14 | mRNA | 5.50E-05 | 0.022291252 | 1.246252232 | 0.74193381 | 0 | 0.003815114 | 0.014693587 | 0.136234648 | 1.112824123 |
| RTF2 | mRNA | 2.54E-05 | 0.010279595 | 0.891709576 | 0.827428004 | 0 | 0.006320997 | 0.030755306 | 0.022936839 | 0.532270619 |
| RTKN2 | mRNA | 1.34E-05 | 0.005426974 | 1.205708412 | 0.751635527 | 0 | 0.026886828 | 0.377196377 | 0.107830113 | 1.960273669 |
| RTN3 | mRNA | 2.21E-06 | 0.000897072 | 1.393436541 | 0.707073961 | 0 | 0.02058634 | 0.078925826 | 0.045033193 | 0.546800152 |
| RUBCNL | mRNA | 1.23E-05 | 0.004972989 | 0.940023474 | 0.815760036 | 0 | 0.10900292 | 0.211256137 | 0.303210758 | 4.192925978 |
| RYR2 | mRNA | 2.73E-05 | 0.011042555 | 1.091970308 | 0.779012829 | 0 | -0.06106259 | -0.41056386 | -0.32569468 | -0.21694936 |
| S100A12 | mRNA | 1.67E-05 | 0.00674865 | 0.069618421 | 0.995215323 | 0 | 0.14548348 | 0.163968056 | 0.232423066 | 5.428078528 |
| S100A14 | mRNA | 1.77E-05 | 0.007154772 | 0.80737397 | 0.847702476 | 0 | 0.062673214 | 0.399416759 | 0.326221805 | 0.910699429 |
| S100A8 | mRNA | 4.82E-05 | 0.019538907 | 0.612678578 | 0.893523868 | 0 | 0.03121619 | 0.039631057 | 0.131983993 | 3.348743752 |
| S100B | mRNA | 6.94E-05 | 0.028123401 | 1.03140689 | 0.793653024 | 0 | 0.018574197 | 0.191606952 | 0.167855332 | 0.113477732 |
| S1PR4 | mRNA | 4.59E-05 | 0.018573595 | 0.317584201 | 0.956688145 | 0 | 0.031251437 | 0.127237514 | 0.20758881 | 2.395147268 |
| SECTM1 | mRNA | 2.36E-05 | 0.009576818 | 0.14556817 | 0.985857322 | 0 | 0.047851141 | 0.115323777 | 0.197418549 | 3.098232172 |
| SELL | mRNA | 4.59E-06 | 0.00185717 | 0.372991784 | 0.945763473 | 0 | 0.102738718 | 0.237137709 | 0.203084619 | 4.901944191 |
| SELPLG | mRNA | 7.52E-05 | 0.030460146 | 0.170362607 | 0.98222571 | 0 | 0.034613607 | 0.158075787 | 0.281202842 | 0.560686594 |
| SEMA3D | mRNA | 1.15E-05 | 0.004639371 | 0.888155187 | 0.828285254 | 0 | 0.060332827 | 0.224615129 | 0.193011584 | 2.595017826 |
| SEMA4D | mRNA | 5.90E-06 | 0.002389821 | 0.84436595 | 0.83882846 | 0 | 0.047130422 | 0.226544671 | 0.378042331 | 1.107415189 |
| SEMA5A | mRNA | 1.46E-06 | 0.000589992 | 0.844626494 | 0.838765841 | 0 | 0.048761146 | 0.186409984 | 0.150962014 | 1.407588625 |
| SERP2 | mRNA | 2.26E-05 | 0.009171643 | 0.466495446 | 0.92618809 | 0 | -0.01312277 | -0.04553922 | -0.11368089 | -0.49672429 |
| SERTAD4 | mRNA | 5.47E-05 | 0.022173254 | 0.226724415 | 0.973163945 | 0 | 0.032841172 | 0.29369193 | 0.596010897 | 1.757471438 |
| SETD5 | mRNA | 4.58E-05 | 0.018564591 | 0.410194016 | 0.938128692 | 0 | 0.018838588 | 0.055778598 | 0.029210659 | 0.573667395 |
| SETX | mRNA | 1.14E-04 | 0.046306038 | 0.598045593 | 0.896879644 | 0 | 0.0078862 | 0.023194023 | 0.026318675 | 0.585712605 |
| SFR1 | mRNA | 5.76E-05 | 0.023342337 | 0.131517645 | 0.987803811 | 0 | 0.017923318 | 0.023709233 | 0.03085717 | 0.763726391 |
| SFRP4 | mRNA | 4.46E-05 | 0.018081699 | 0.626619178 | 0.890312389 | 0 | 0.099349214 | 0.184835881 | 0.365899643 | 1.283309038 |
| SGCG | mRNA | 3.67E-05 | 0.014867311 | 1.652383427 | 0.647571404 | 0 | 0.04089785 | 0.657629493 | 0.223228372 | 0.787803456 |
| SH2D7 | mRNA | 3.52E-05 | 0.014250308 | 0.050144513 | 0.997058085 | 0 | 0.015961771 | 0.172750152 | 0.013900137 | 1.164388092 |
| SH3BGRL2 | mRNA | 1.26E-05 | 0.005090513 | 2.675384854 | 0.444426601 | 0 | 0.046993396 | 0.130055404 | 0.148282767 | 0.768669005 |
| SH3BP5L | mRNA | 2.57E-05 | 0.010407545 | 0.188202251 | 0.979471005 | 0 | 0.008945278 | 0.035897869 | 0.048047406 | 0.491500449 |
| SHC3 | mRNA | 8.72E-06 | 0.003530945 | 0.561503129 | 0.905185956 | 0 | 0.028933691 | 0.312363655 | 0.526184278 | 1.118411666 |
| SHCBP1L | mRNA | 2.18E-05 | 0.008829021 | 2.541971019 | 0.467753709 | 0 | 0.00933981 | 0.263401064 | 0.510641888 | 1.225690964 |
| SHROOM4 | mRNA | 8.57E-06 | 0.003470287 | 0.853453945 | 0.836643369 | 0 | 0.042046509 | 0.072015813 | 0.037470065 | 0.689051365 |
| SIPA1L1 | mRNA | 4.88E-06 | 0.001977538 | 1.750762511 | 0.625707715 | 0 | 0.029460732 | 0.029028365 | 0.038808579 | 0.421414173 |
| SKAP1 | mRNA | 7.86E-05 | 0.031832528 | 0.265195491 | 0.966435597 | 0 | 0.04099691 | 0.135805484 | 0.193571856 | 2.653967824 |
| SLC19A3 | mRNA | 8.08E-06 | 0.003271728 | 2.179311658 | 0.536034817 | 0 | -0.03504502 | -0.11133164 | -0.1580344 | -0.40826897 |
| SLC1A6 | mRNA | 3.84E-05 | 0.015556346 | 1.392075172 | 0.707393402 | 0 | 0.008302054 | 0.326170063 | 0.169204091 | 1.5612128 |
| SLC22A10 | mRNA | 2.20E-05 | 0.008923325 | 0.566229566 | 0.904117911 | 0 | 0.087402323 | 0.186029277 | 0.140463482 | 3.707069991 |
| SLC24A1 | mRNA | 7.51E-06 | 0.003043314 | 1.768546991 | 0.621803263 | 0 | -0.01263315 | -0.10824611 | -0.10520479 | -0.71070579 |
| SLC24A4 | mRNA | 1.97E-05 | 0.00796607 | 0.329660706 | 0.954356903 | 0 | 0.061418721 | 0.076660735 | 0.258260797 | 1.878013341 |
| SLC25A37 | mRNA | 3.36E-05 | 0.013603924 | 0.39655204 | 0.940953838 | 0 | 0.109622279 | 0.03061826 | 0.15646239 | 2.014206433 |
| SLC26A2 | mRNA | 6.88E-05 | 0.027871292 | 0.721278091 | 0.86818808 | 0 | 0.032688196 | 0.060906054 | 0.021623155 | 1.164554108 |
| SLC29A2 | mRNA | 1.01E-04 | 0.040844944 | 0.168158458 | 0.982558119 | 0 | -0.01686973 | -0.03419346 | -0.07848434 | -0.7420293 |
| SLC2A1 | mRNA | 3.55E-05 | 0.014376363 | 1.17558734 | 0.758865232 | 0 | -0.15896891 | -0.04019656 | -0.06768371 | -0.53169566 |
| SLC2A12 | mRNA | 1.57E-05 | 0.006369065 | 1.251369268 | 0.740712008 | 0 | -0.07476305 | -0.15637206 | -0.14114614 | -0.0235376 |
| SLC2A3 | mRNA | 5.81E-05 | 0.023538527 | 1.206582315 | 0.751426037 | 0 | 0.010199379 | 0.046719107 | 0.123817345 | 0.661078602 |
| SLC31A1 | mRNA | 1.54E-05 | 0.006239693 | 0.779434467 | 0.854379447 | 0 | -0.04239872 | -0.03674844 | -0.05908302 | -0.24195963 |
| SLC40A1 | mRNA | 9.91E-06 | 0.004013364 | 0.70251169 | 0.872613016 | 0 | 0.065554469 | 0.088224125 | 0.120988262 | 0.903651449 |
| SLC6A3 | mRNA | 9.55E-05 | 0.03866553 | 1.862654966 | 0.601396331 | 0 | 0.013953388 | 0.017333464 | 0.015999098 | 0.153757147 |
| SLC7A11 | mRNA | 6.14E-05 | 0.024868259 | 0.995385879 | 0.802368437 | 0 | 0.167628948 | 0.250451829 | 0.044806566 | 0.408700493 |
| SLC7A4 | mRNA | 2.42E-05 | 0.009806654 | 1.27047275 | 0.736156267 | 0 | -0.07765705 | -0.16164428 | -0.13790936 | -0.08765131 |
| SLC8A2 | mRNA | 8.76E-05 | 0.035490475 | 1.156019485 | 0.763570804 | 0 | -0.00042317 | -0.2207312 | -0.16384604 | -2.7525263 |
| SLC9A1 | mRNA | 7.79E-05 | 0.031542508 | 1.109066073 | 0.774885961 | 0 | 0.005976695 | 0.046864644 | 0.044442196 | 0.657644953 |
| SMCHD1 | mRNA | 1.09E-04 | 0.044190598 | 1.271924881 | 0.735810339 | 0 | 0.051003723 | 0.001830167 | 0.129911657 | 0.919530939 |
| SMLR1 | mRNA | 1.20E-04 | 0.048433324 | 0.619487716 | 0.891956972 | 0 | 0.127674236 | 0.105644912 | 0.757707237 | 1.027527621 |
| SMS | mRNA | 1.80E-05 | 0.007273718 | 2.22539113 | 0.526964442 | 0 | 0.013950368 | 0.072997022 | 0.015101159 | 0.41088347 |
| SNX19 | mRNA | 1.14E-04 | 0.046320728 | 0.854568772 | 0.836375204 | 0 | 0.009241434 | 0.068248494 | 0.006790212 | 0.564992158 |
| SNX8 | mRNA | 2.00E-05 | 0.008107289 | 1.019958307 | 0.79642279 | 0 | -0.00386413 | -0.05376566 | -0.05672113 | -0.41359818 |
| SORD | mRNA | 2.16E-05 | 0.00874846 | 1.086409703 | 0.780355807 | 0 | -0.08177639 | -0.05319749 | -0.16097304 | -0.24296933 |
| SORL1 | mRNA | 3.46E-07 | 0.000140271 | 0.605307253 | 0.895216331 | 0 | 0.214915546 | 0.160926244 | 0.207239448 | 3.322977136 |
| SOS2 | mRNA | 6.48E-05 | 0.026225003 | 0.219655164 | 0.974355596 | 0 | 0.007778471 | 0.036452675 | 0.038056681 | 0.514987315 |
| SOX7 | mRNA | 9.83E-05 | 0.039799071 | 1.723430043 | 0.631737385 | 0 | -0.03741541 | -0.2431545 | -0.10398077 | -0.13369117 |
| SP5 | mRNA | 4.76E-05 | 0.019276846 | 0.716755012 | 0.869256104 | 0 | -0.02363452 | -0.01402581 | -0.75041687 | -1.71059039 |
| SPAG4 | mRNA | 2.24E-05 | 0.009088238 | 0.888642128 | 0.828167824 | 0 | -0.06225138 | -0.07031326 | -0.16318142 | -0.32205768 |
| SPATA6 | mRNA | 5.06E-05 | 0.020498057 | 0.653333981 | 0.884121398 | 0 | 0.013857122 | 0.093091537 | 0.021963361 | 0.71579014 |
| SPRR2F | mRNA | 2.77E-06 | 0.001120273 | 2.484351278 | 0.478125797 | 0 | 0.044673914 | 0.112321069 | 0.317540061 | 0.428485866 |
| SPTA1 | mRNA | 1.21E-04 | 0.049020946 | 0.79211395 | 0.851352328 | 0 | 0.003904283 | 0.145499431 | 0.240304532 | 3.349609095 |
| SRA1 | mRNA | 2.07E-06 | 0.000837362 | 1.399815588 | 0.705577959 | 0 | -0.04365185 | -0.07084733 | -0.05086533 | -0.11697913 |
| SRCAP | mRNA | 7.95E-05 | 0.032191262 | 1.232311786 | 0.745265496 | 0 | 0.016622892 | 0.037528463 | 0.056490697 | 0.03753925 |
| SRD5A1 | mRNA | 1.08E-04 | 0.043749882 | 1.149798633 | 0.765068095 | 0 | 0.016370162 | 0.035764111 | 0.009138696 | 0.296594111 |
| SRPK1 | mRNA | 7.91E-05 | 0.032032983 | 0.703474352 | 0.872386444 | 0 | 0.022695553 | 0.004700145 | 0.064362605 | 0.632241145 |
| SSUH2 | mRNA | 9.31E-05 | 0.037713013 | 0.284119954 | 0.962986886 | 0 | 0.009791411 | 0.04912128 | 0.158006722 | 1.096959429 |
| ST3GAL2 | mRNA | 5.70E-05 | 0.02306606 | 0.687391399 | 0.876165536 | 0 | 0.014449258 | 0.133573187 | 0.104284772 | 0.272781522 |
| ST6GALNAC2 | mRNA | 6.43E-08 | 0.00002605 | 2.268661221 | 0.518551156 | 0 | 0.057352769 | 0.288609145 | 0.217709858 | 0.898485283 |
| STAM | mRNA | 9.91E-05 | 0.040126529 | 2.208110725 | 0.530352579 | 0 | 0.002472641 | 0.029763901 | 0.000990602 | 0.53552924 |
| STAMBPL1 | mRNA | 2.67E-05 | 0.010815563 | 2.932365282 | 0.402171102 | 0 | 0.041258938 | 0.092297054 | 0.035321089 | 1.005139364 |
| STAU2 | mRNA | 8.76E-06 | 0.003548479 | 0.978141437 | 0.806540876 | 0 | 0.006306308 | 0.03126895 | 0.032137823 | 1.019031756 |
| STEAP1B | mRNA | 4.98E-05 | 0.020182447 | 0.90355042 | 0.824570927 | 0 | -0.03447841 | -0.09902468 | -0.0817506 | -0.78981593 |
| STK10 | mRNA | 1.01E-05 | 0.00408113 | 1.158912718 | 0.762874647 | 0 | 0.011171843 | 0.047748286 | 0.148204507 | 0.668180926 |
| STK32C | mRNA | 7.56E-06 | 0.003061795 | 0.808296904 | 0.847481512 | 0 | -0.03083971 | -0.10077403 | -0.0634955 | -0.15069226 |
| STK4 | mRNA | 5.13E-05 | 0.020787129 | 0.815747005 | 0.845696968 | 0 | 0.036794825 | 0.044361614 | 0.088152317 | 0.101640137 |
| STK40 | mRNA | 8.45E-06 | 0.003422899 | 2.375726376 | 0.498169144 | 0 | 0.002000713 | 0.068401703 | 0.065907651 | 0.742246848 |
| STN1 | mRNA | 2.45E-06 | 0.000990428 | 2.362034378 | 0.500741036 | 0 | 0.020677136 | 0.027060505 | 0.067313614 | 0.511652749 |
| STYXL1 | mRNA | 5.96E-05 | 0.024131836 | 0.614087322 | 0.893199966 | 0 | 0.032602901 | 0.015803151 | 0.024536306 | 0.330777983 |
| SUDS3 | mRNA | 1.03E-04 | 0.041648659 | 0.111964217 | 0.990364 | 0 | 0.016396535 | 0.045031919 | 0.028071722 | 0.137291445 |
| SULT4A1 | mRNA | 1.01E-04 | 0.04099564 | 0.868877389 | 0.832931162 | 0 | -0.00429312 | -0.11730526 | -0.03664968 | -0.99055735 |
| SUSD6 | mRNA | 4.35E-06 | 0.001760971 | 0.841730114 | 0.839461876 | 0 | 0.030707595 | 0.049203475 | 0.118091388 | 0.541502427 |
| SUV39H1 | mRNA | 2.24E-05 | 0.009074022 | 0.32083694 | 0.956063143 | 0 | -0.04715745 | -0.0992791 | -0.12294525 | -0.32031245 |
| SVOPL | mRNA | 4.00E-05 | 0.016209364 | 1.666499379 | 0.644407251 | 0 | 0.02349563 | 0.181397668 | 0.255247929 | 0.962270302 |
| SYT9 | mRNA | 7.95E-06 | 0.0032196 | 0.442447676 | 0.931340726 | 0 | 0.034688659 | 0.095905534 | 0.017844587 | 1.471984935 |
| TACR2 | mRNA | 3.61E-05 | 0.014632263 | 0.698094308 | 0.873652095 | 0 | 0.015010816 | 0.102685685 | 0.073374791 | 0.177168957 |
| TAF7 | mRNA | 2.51E-05 | 0.010146906 | 0.626742079 | 0.890284016 | 0 | 0.003433348 | 0.062718286 | 0.015873442 | 0.383869629 |
| TAGAP | mRNA | 1.17E-05 | 0.004741257 | 0.239259362 | 0.971015665 | 0 | 0.212402274 | 0.126257072 | 0.258664471 | 3.109710106 |
| TAPT1 | mRNA | 2.07E-05 | 0.008392096 | 1.132380843 | 0.769263524 | 0 | 0.019886041 | 0.04515303 | 0.015559775 | 0.805983304 |
| TASP1 | mRNA | 5.62E-05 | 0.022760402 | 0.829867329 | 0.842310626 | 0 | 0.023216939 | 0.044421316 | 0.03665934 | 1.106583994 |
| TATDN2 | mRNA | 1.69E-05 | 0.006840584 | 1.148260812 | 0.765438326 | 0 | 0.019536273 | 0.079808583 | 0.012898852 | 0.398637436 |
| TBC1D10C | mRNA | 3.63E-06 | 0.001469529 | 0.382332298 | 0.943867554 | 0 | 0.01377573 | 0.211816873 | 0.190520082 | 3.75779729 |
| TCEA3 | mRNA | 7.63E-05 | 0.030886172 | 1.137051993 | 0.768137943 | 0 | 0.038208187 | 0.06525577 | 0.091305683 | 0.108268775 |
| TCP11L2 | mRNA | 3.14E-05 | 0.012712065 | 0.377785152 | 0.944792347 | 0 | 0.040812176 | 0.051168645 | 0.170254896 | 0.790761568 |
| TCTE3 | mRNA | 1.14E-04 | 0.04612596 | 0.210227722 | 0.975921547 | 0 | 0.004207592 | 0.064413579 | 0.092521696 | 0.464068254 |
| TECPR2 | mRNA | 3.08E-05 | 0.012486494 | 0.467929715 | 0.925878459 | 0 | 0.088799108 | 0.179511265 | 0.117575063 | 0.142234686 |
| TECR | mRNA | 6.37E-05 | 0.02578476 | 0.40557482 | 0.939088475 | 0 | -0.0530659 | -0.07245166 | -0.04896404 | -0.09596283 |
| TFCP2L1 | mRNA | 9.35E-05 | 0.03786371 | 0.39763001 | 0.940731643 | 0 | 0.032610916 | 0.25497455 | 0.276868999 | 0.537218858 |
| THAP8 | mRNA | 6.43E-05 | 0.02604682 | 1.613487679 | 0.656335461 | 0 | -0.05621604 | -0.02075709 | -0.08437383 | -0.10422909 |
| TIAM2 | mRNA | 3.14E-05 | 0.012726756 | 0.80010484 | 0.849441957 | 0 | 0.00355177 | 0.097599224 | 0.080845852 | 0.846408098 |
| TIMP1 | mRNA | 5.63E-05 | 0.022787888 | 2.663534287 | 0.446459872 | 0 | -0.04579512 | -0.06863072 | -0.02731791 | -0.00085453 |
| TLR1 | mRNA | 8.68E-05 | 0.035139324 | 1.028674678 | 0.794313993 | 0 | 0.088494153 | 0.027435127 | 0.229679428 | 4.007980339 |
| TLR6 | mRNA | 3.95E-05 | 0.015978107 | 0.06822232 | 0.995356587 | 0 | 0.130655896 | 0.111893315 | 0.256256498 | 1.116364815 |
| TMCC3 | mRNA | 7.48E-05 | 0.03027533 | 0.472721293 | 0.924842227 | 0 | 0.013545701 | 0.028408482 | 0.100767956 | 0.311812738 |
| TMEM154 | mRNA | 3.70E-06 | 0.001499384 | 0.191584863 | 0.978936219 | 0 | 0.156972777 | 0.293555235 | 0.287747535 | 4.415882033 |
| TMEM160 | mRNA | 1.13E-04 | 0.045805137 | 1.336442763 | 0.720498106 | 0 | -0.10563274 | -0.03383489 | -0.01186366 | -0.36407483 |
| TMEM215 | mRNA | 4.54E-05 | 0.018390674 | 0.372270459 | 0.945909275 | 0 | 0.026320656 | 0.132493548 | 0.845353671 | 1.556890709 |
| TMEM30B | mRNA | 8.21E-05 | 0.033250403 | 0.410042177 | 0.938160292 | 0 | 0.029579366 | 0.121796219 | 0.157858363 | 1.730921754 |
| TMEM35A | mRNA | 4.30E-05 | 0.017406881 | 0.366675407 | 0.947037184 | 0 | 0.024942216 | 0.194954752 | 0.241589587 | 1.063876252 |
| TMEM53 | mRNA | 1.01E-04 | 0.040751588 | 0.428278403 | 0.934340838 | 0 | -0.04318568 | -0.01979833 | -0.03125331 | -0.1647087 |
| TMEM63C | mRNA | 5.22E-05 | 0.021143494 | 0.545655946 | 0.908752414 | 0 | 0.025499715 | 0.0598169 | 0.064771186 | 1.340186166 |
| TMEM74 | mRNA | 9.58E-05 | 0.038795849 | 0.402976031 | 0.939627033 | 0 | 0.014828258 | 0.163197291 | 0.135696844 | 0.528080518 |
| TMEM86B | mRNA | 7.92E-05 | 0.032056677 | 0.356849856 | 0.949004697 | 0 | -0.01380835 | -0.04358092 | -0.01653993 | -1.14910483 |
| TMEM9 | mRNA | 1.03E-05 | 0.004158848 | 1.802317379 | 0.614430778 | 0 | -0.01333969 | -0.05095622 | -0.05414354 | -0.59687872 |
| TNFAIP2 | mRNA | 2.90E-05 | 0.011760023 | 0.807191539 | 0.84774615 | 0 | 0.009756048 | 0.150871876 | 0.201587768 | 1.633109465 |
| TNFRSF19 | mRNA | 9.00E-05 | 0.036455786 | 1.211020853 | 0.750362288 | 0 | -0.0061594 | -0.30951315 | -0.17409489 | -0.63318821 |
| TNFRSF4 | mRNA | 7.77E-05 | 0.031471899 | 2.257770003 | 0.520659291 | 0 | 0.000668599 | 0.051204365 | 0.145893716 | 0.272348399 |
| TNFRSF9 | mRNA | 3.33E-05 | 0.01350109 | 0.272062078 | 0.965194256 | 0 | 0.052199863 | 0.043516343 | 0.494272607 | 1.337827272 |
| TNRC18 | mRNA | 2.75E-05 | 0.011152024 | 2.003892973 | 0.571599097 | 0 | 0.011553528 | 0.009222028 | 0.04519591 | 0.352165063 |
| TPMT | mRNA | 2.24E-05 | 0.009057436 | 2.723253179 | 0.436290113 | 0 | -0.04914275 | -0.02441229 | -0.06091811 | -0.02293204 |
| TRANK1 | mRNA | 5.15E-08 | 0.00002085 | 0.326489439 | 0.954971922 | 0 | 0.093012209 | 0.166484285 | 0.212605337 | 0.86002858 |
| TRAPPC9 | mRNA | 6.14E-05 | 0.024870155 | 0.669054886 | 0.880457268 | 0 | 0.007172106 | 0.077191343 | 0.005055217 | 0.399556091 |
| TRERF1 | mRNA | 1.06E-04 | 0.042910151 | 0.366374505 | 0.947097689 | 0 | 0.054138308 | 0.036310511 | 0.1118279 | 0.342847707 |
| TRIM33 | mRNA | 3.89E-05 | 0.015764383 | 0.55502007 | 0.906647738 | 0 | 0.007245536 | 0.118910054 | 0.029902067 | 0.495366143 |
| TRMT5 | mRNA | 8.48E-05 | 0.034355512 | 1.154791847 | 0.763866234 | 0 | 0.008011943 | 0.051744689 | 0.014518902 | 0.73584314 |
| TRPC4AP | mRNA | 1.11E-04 | 0.045122737 | 0.317340099 | 0.95673496 | 0 | 0.003241723 | 0.018335901 | 0.038508099 | 0.341169618 |
| TTL | mRNA | 2.36E-05 | 0.009541276 | 2.861075239 | 0.413544279 | 0 | 0.018098492 | 0.057157547 | 0.005625169 | 0.476763917 |
| TUBB1 | mRNA | 5.39E-05 | 0.021832054 | 1.503229199 | 0.681525234 | 0 | 0.127173657 | 0.11299035 | 0.164012926 | 3.393735107 |
| TXNIP | mRNA | 3.75E-05 | 0.015171548 | 1.560228188 | 0.668441047 | 0 | 0.012370951 | 0.078645619 | 0.104724154 | 1.525769997 |
| UBE2B | mRNA | 1.43E-05 | 0.005807033 | 1.393399674 | 0.707082611 | 0 | 0.014563383 | 0.057320933 | 0.044896664 | 0.285751483 |
| UBXN2B | mRNA | 2.20E-06 | 0.000891385 | 1.580461996 | 0.663828 | 0 | 0.026982671 | 0.129747487 | 0.093731061 | 1.019408131 |
| UBXN4 | mRNA | 3.70E-05 | 0.014965406 | 0.605576191 | 0.895154654 | 0 | -0.00956654 | -0.03725742 | -0.05405911 | -0.6345426 |
| UFM1 | mRNA | 9.04E-05 | 0.036592266 | 0.500003891 | 0.918890557 | 0 | -0.0268985 | -0.03294689 | -0.04731672 | -0.02914104 |
| UGCG | mRNA | 2.92E-05 | 0.011833476 | 0.524091466 | 0.913567624 | 0 | 0.016246518 | 0.033871308 | 0.064984653 | 0.647407575 |
| ULK4 | mRNA | 3.18E-05 | 0.012870344 | 2.130955654 | 0.545675654 | 0 | 0.020113634 | 0.094002742 | 0.066763746 | 0.390932883 |
| UNC79 | mRNA | 6.71E-05 | 0.027194579 | 0.492787078 | 0.920473178 | 0 | 0.003146614 | 0.088883941 | 0.012795012 | 1.192312583 |
| USP11 | mRNA | 6.52E-05 | 0.026405554 | 0.867310634 | 0.833308466 | 0 | -0.05795754 | -0.01986538 | -0.06116344 | -0.29981954 |
| USP35 | mRNA | 4.13E-05 | 0.016735855 | 2.254533133 | 0.521287063 | 0 | 0.043770137 | 0.021422024 | 0.020673358 | 0.319125713 |
| VASH1 | mRNA | 9.78E-05 | 0.039623259 | 1.584849088 | 0.662830046 | 0 | -0.02131511 | -0.02444522 | -0.01102332 | -1.02779999 |
| VMAC | mRNA | 1.10E-04 | 0.044455028 | 1.395583923 | 0.70657021 | 0 | 0.012692996 | 0.023159291 | 0.031154631 | 0.718810898 |
| VOPP1 | mRNA | 1.04E-05 | 0.004218084 | 2.558112379 | 0.464880353 | 0 | 0.00719679 | 0.071875365 | 0.081603238 | 0.006715183 |
| VSIR | mRNA | 4.82E-05 | 0.019519477 | 0.948608307 | 0.813684433 | 0 | 0.030324917 | 0.028316114 | 0.191645075 | 1.83946117 |
| VSTM2B | mRNA | 9.37E-05 | 0.037939532 | 0.68049901 | 0.877780909 | 0 | -0.00074133 | -0.04827 | -0.22904558 | -2.20551137 |
| VWC2 | mRNA | 9.21E-06 | 0.0037314 | 1.606746595 | 0.657861044 | 0 | 0.011961526 | 0.521519438 | 0.574480364 | 0.666823635 |
| VWDE | mRNA | 4.23E-05 | 0.017148612 | 1.045917038 | 0.790143286 | 0 | 0.02160747 | 0.058861073 | 0.107368998 | 1.26468461 |
| WAS | mRNA | 1.01E-04 | 0.040812246 | 0.614921469 | 0.893008109 | 0 | 0.010600134 | 0.026867933 | 0.171558514 | 1.807252972 |
| WBP4 | mRNA | 7.60E-06 | 0.003079329 | 2.223677878 | 0.527299638 | 0 | -0.05226722 | -0.05691132 | -0.04803841 | -0.32863946 |
| WDFY3 | mRNA | 1.12E-05 | 0.004539854 | 0.260942193 | 0.967198631 | 0 | 0.049100962 | 0.042216404 | 0.088102268 | 1.010528067 |
| WDR60 | mRNA | 8.09E-06 | 0.003276467 | 0.193444259 | 0.978640616 | 0 | -0.0479416 | -0.06421613 | -0.1148204 | -0.76065793 |
| WFDC13 | mRNA | 2.11E-05 | 0.008540423 | 0.670075628 | 0.880218856 | 0 | 0.062360369 | 0.062761233 | 0.014331811 | 0.86438254 |
| WNT10A | mRNA | 6.53E-06 | 0.002643825 | 0.566031536 | 0.904162699 | 0 | 0.029683451 | 0.11669487 | 0.319998066 | 2.080494145 |
| WNT5B | mRNA | 1.22E-05 | 0.004944081 | 0.724885936 | 0.867335498 | 0 | 0.021112403 | 0.380928365 | 0.748103777 | 1.156307423 |
| XYLB | mRNA | 1.06E-04 | 0.043109184 | 2.777078381 | 0.427287137 | 0 | -0.00308903 | -0.32015842 | -0.09035533 | -0.09489499 |
| ZBED5 | mRNA | 7.67E-05 | 0.031061511 | 2.26785725 | 0.518706556 | 0 | 0.001584242 | 0.007726141 | 0.030308221 | 0.965525522 |
| ZBTB2 | mRNA | 1.29E-06 | 0.000523647 | 1.074611589 | 0.78320621 | 0 | 0.019270579 | 0.077919122 | 0.049434804 | 0.515590231 |
| ZBTB32 | mRNA | 5.81E-05 | 0.023515781 | 1.185009514 | 0.756601849 | 0 | 0.011007771 | 0.082575768 | 0.067861118 | 2.206840174 |
| ZBTB7C | mRNA | 7.80E-05 | 0.031585158 | 0.680751876 | 0.87772169 | 0 | -0.02310214 | -0.06341624 | -0.13536014 | -0.04513151 |
| ZBTB8B | mRNA | 9.26E-05 | 0.037510663 | 0.127000489 | 0.988411149 | 0 | 0.039312061 | 0.142002948 | 0.445133133 | 0.756203196 |
| ZCCHC17 | mRNA | 8.17E-06 | 0.003309165 | 0.723753324 | 0.867603213 | 0 | -0.01927718 | -0.11132812 | -0.08887739 | -0.22101039 |
| ZCRB1 | mRNA | 1.72E-05 | 0.006980381 | 1.351299994 | 0.716988909 | 0 | -0.00973406 | -0.04200484 | -0.09196612 | -0.29753749 |
| ZDHHC18 | mRNA | 2.31E-06 | 0.00093593 | 0.448851929 | 0.929975821 | 0 | 0.08274629 | 0.11324625 | 0.230012631 | 0.617583447 |
| ZFP90 | mRNA | 5.21E-05 | 0.02110653 | 1.65428352 | 0.647144974 | 0 | 0.001418848 | 0.103754801 | 0.01894999 | 0.37908283 |
| ZG16B | mRNA | 4.77E-05 | 0.019338452 | 0.341945082 | 0.951955986 | 0 | 0.037808563 | 0.110656752 | 0.126715991 | 1.135594535 |
| ZNF100 | mRNA | 1.54E-05 | 0.006252488 | 0.502849862 | 0.918264865 | 0 | 0.076333526 | 0.075032645 | 0.065558025 | 0.509985568 |
| ZNF136 | mRNA | 6.09E-05 | 0.02466117 | 0.744919823 | 0.862590876 | 0 | 0.017218028 | 0.032475454 | 0.047173151 | 1.127284555 |
| ZNF184 | mRNA | 7.41E-05 | 0.029990996 | 1.192806181 | 0.754730199 | 0 | 6.16E-05 | 0.136140382 | 0.037783753 | 0.938588505 |
| ZNF250 | mRNA | 4.07E-05 | 0.016496067 | 2.7394461 | 0.433565411 | 0 | 0.028362157 | 0.033536759 | 0.046613236 | 0.426513855 |
| ZNF367 | mRNA | 1.67E-05 | 0.006762866 | 0.191527553 | 0.978945311 | 0 | 0.01666043 | 0.181956195 | 0.731758741 | 1.991484599 |
| ZNF395 | mRNA | 5.67E-05 | 0.022959435 | 0.760173813 | 0.858967251 | 0 | -0.03004682 | -0.05362318 | -0.03873818 | -0.55234488 |
| ZNF521 | mRNA | 4.95E-06 | 0.00200455 | 1.430731418 | 0.69834719 | 0 | -0.09849291 | -0.11980717 | -0.17596838 | -0.37415758 |
| ZNF561 | mRNA | 5.68E-05 | 0.022992134 | 0.399199546 | 0.9404078 | 0 | 0.011678087 | 0.033747368 | 0.02508748 | 0.7518919 |
| ZNF626 | mRNA | 1.12E-04 | 0.045313241 | 1.127125565 | 0.770530227 | 0 | 0.016162403 | 0.009616603 | 0.028413992 | 0.704336094 |
| ZNF646 | mRNA | 5.62E-05 | 0.02274192 | 0.913359695 | 0.822202669 | 0 | 0.00430918 | 0.090454771 | 0.043458266 | 0.1289422 |
| ZNF674 | mRNA | 1.06E-04 | 0.042976969 | 0.562924152 | 0.904865049 | 0 | 0.005996359 | 0.084083457 | 0.064410743 | 0.832595271 |
| ZNF74 | mRNA | 7.68E-05 | 0.031111269 | 1.269854477 | 0.736303569 | 0 | 0.006854489 | 0.065974132 | 0.021232877 | 0.362039283 |
| ZNF774 | mRNA | 2.40E-06 | 0.000972894 | 0.505694493 | 0.917638587 | 0 | 0.02816786 | 0.10504992 | 0.207944193 | 1.291175653 |
| ZNF808 | mRNA | 8.77E-05 | 0.035524595 | 0.588060689 | 0.899160084 | 0 | 0.02750383 | 0.041772168 | 0.01402379 | 0.385564146 |
| ZNF814 | mRNA | 3.04E-05 | 0.012304047 | 1.910723748 | 0.591141454 | 0 | -0.00012911 | -0.04565238 | -0.03810089 | -0.21961386 |
| ZNF862 | mRNA | 5.13E-06 | 0.002077528 | 1.337212845 | 0.720316054 | 0 | 0.034960637 | 0.18543739 | 0.040510282 | 0.135491383 |
| ZNFX1 | mRNA | 1.01E-04 | 0.040713677 | 0.737983557 | 0.864235521 | 0 | 0.017615381 | 0.016871064 | 0.05747307 | 0.1965601 |
