# Supplementary material for: Identification of Crucial lncRNAs, miRNAs, mRNAs, and Potential Therapeutic Compounds for Polycystic Ovary Syndrome by Bioinformatics Analysis
Source: Biomed Res Int. 2020 Nov 6;2020:1817094. doi: 10.1155/2020/1817094 (PMC7666708; doi:10.1155/2020/1817094)
Supplement: Supplementary 2 — Table S2: all differential miRNAs in GSE13572 dataset. [file 1817094.f2.docx]

**Table S2 Differentially expressed miRNAs in GSE13572**

| miRNA | P-value | FDR | logFC |
| --- | --- | --- | --- |
| hsa-miR-455 | 2.240E-04 | 1.814E-02 | 1.00782191 |
| hsa-miR-4741 | 5.783E-04 | 4.684E-02 | 1.377738204 |
| hsa-miR-4467 | 5.335E-04 | 4.321E-02 | 3.61538841 |
| hsa-miR-4665 | 1.264E-04 | 1.024E-02 | 1.40385254 |
| hsa-miR-4448 | 2.328E-04 | 1.885E-02 | -5.552700177 |
| hsa-miR-1237 | 5.165E-04 | 4.184E-02 | -3.376309456 |
| hsa-miR-3938 | 2.695E-04 | 2.183E-02 | -3.307603136 |
| hsa-miR-3159 | 3.564E-04 | 2.886E-02 | -3.224547764 |
| hsa-miR-4473 | 2.081E-04 | 1.685E-02 | -2.327549154 |
| hsa-miR-4521 | 2.603E-04 | 2.109E-02 | -2.302423228 |
| hsa-miR-1537 | 6.078E-04 | 4.923E-02 | -2.106021039 |
| hsa-miR-3139 | 3.794E-04 | 3.073E-02 | -2.035850282 |
| hsa-miR-4711 | 4.140E-04 | 3.353E-02 | -1.870010072 |
| hsa-miR-193a | 1.879E-04 | 1.522E-02 | -1.76627954 |
| hsa-miR-433 | 9.200E-05 | 7.453E-03 | -1.655222677 |
| hsa-miR-3133 | 1.339E-04 | 1.084E-02 | -1.525302642 |
| hsa-miR-431 | 1.989E-04 | 1.611E-02 | -1.385705653 |
| hsa-miR-487a | 7.930E-05 | 6.421E-03 | -1.35646518 |
| hsa-miR-4638 | 4.277E-04 | 3.464E-02 | -1.267148703 |
| hsa-miR-489 | 2.585E-04 | 2.094E-02 | -1.247960685 |
| hsa-miR-4512 | 5.965E-04 | 4.832E-02 | -1.223673242 |
| hsa-miR-487b | 1.083E-04 | 8.775E-03 | -1.210456231 |
| hsa-miR-184 | 1.241E-04 | 1.005E-02 | -1.177578577 |
| hsa-miR-1305 | 5.093E-04 | 4.126E-02 | -1.173808189 |
| hsa-miR-326 | 3.858E-04 | 3.125E-02 | -1.16947905 |
| hsa-miR-2110 | 1.587E-04 | 1.286E-02 | -1.049964858 |
| hsa-miR-493 | 7.830E-05 | 6.341E-03 | -1.020930155 |
| hsa-miR-1180 | 4.580E-05 | 3.713E-03 | -0.981517232 |
| hsa-miR-3529 | 2.921E-04 | 2.366E-02 | -0.966677179 |
| hsa-miR-494 | 3.589E-04 | 2.907E-02 | -0.959243281 |
| hsa-miR-411 | 2.007E-04 | 1.626E-02 | -0.933035369 |
| hsa-miR-382 | 9.450E-05 | 7.655E-03 | -0.89302305 |
| hsa-miR-369 | 1.961E-04 | 1.588E-02 | -0.855086384 |
| hsa-miR-543 | 3.137E-04 | 2.541E-02 | -0.828277927 |
| hsa-miR-3074 | 4.954E-04 | 4.013E-02 | -0.819218695 |
| hsa-miR-381 | 2.146E-04 | 1.739E-02 | -0.803860041 |
| hsa-miR-320a | 4.070E-05 | 3.299E-03 | -0.796613467 |
| hsa-miR-485 | 2.366E-04 | 1.917E-02 | -0.779058267 |
| hsa-miR-29a | 6.330E-05 | 5.130E-03 | -0.774997048 |
| hsa-miR-410 | 2.395E-04 | 1.940E-02 | -0.683491804 |
| hsa-miR-134 | 1.776E-04 | 1.438E-02 | -0.615643296 |
| hsa-miR-376b | 4.789E-04 | 3.879E-02 | -0.598691246 |
| hsa-miR-95 | 1.415E-04 | 1.146E-02 | -0.591072386 |
| hsa-miR-1277 | 4.852E-04 | 3.930E-02 | -0.589133344 |
| hsa-miR-539 | 2.685E-04 | 2.175E-02 | -0.581390124 |
| hsa-miR-127 | 2.886E-04 | 2.338E-02 | -0.576207346 |
| hsa-miR-299 | 4.169E-04 | 3.377E-02 | -0.571117298 |
| hsa-miR-409 | 3.790E-04 | 3.070E-02 | -0.531823797 |
| hsa-miR-181d | 6.085E-04 | 4.929E-02 | -0.50304436 |
| hsa-miR-548n | 2.890E-05 | 2.338E-03 | -1.09362973 |
| hsa-miR-4511 | 6.810E-05 | 5.515E-03 | -3.150212428 |
| hsa-miR-1197 | 8.110E-05 | 6.572E-03 | -1.847339611 |
| hsa-miR-597 | 9.910E-05 | 8.026E-03 | -4.464419701 |
| hsa-miR-4749 | 1.073E-04 | 8.693E-03 | -3.495810707 |
| hsa-miR-4781 | 1.117E-04 | 9.044E-03 | -4.819580721 |
| hsa-miR-937 | 1.622E-04 | 1.314E-02 | -1.857524329 |
| hsa-miR-188 | 1.712E-04 | 1.387E-02 | 3.440244899 |
| hsa-miR-504 | 2.201E-04 | 1.783E-02 | -1.526103199 |
| hsa-miR-877 | 2.235E-04 | 1.810E-02 | -1.744911528 |
| hsa-miR-100 | 2.321E-04 | 1.880E-02 | 2.178835063 |
| hsa-miR-758 | 2.337E-04 | 1.893E-02 | -1.033648178 |
| hsa-miR-3136 | 2.692E-04 | 2.181E-02 | 2.800767398 |
| hsa-miR-548p | 2.943E-04 | 2.384E-02 | -2.95206083 |
| hsa-miR-3188 | 3.014E-04 | 2.442E-02 | -3.150520484 |
| hsa-miR-4504 | 3.433E-04 | 2.780E-02 | 3.925972223 |
| hsa-miR-449c | 3.761E-04 | 3.046E-02 | 2.441190556 |
| hsa-miR-3615 | 3.781E-04 | 3.063E-02 | 2.749489094 |
| hsa-miR-2115 | 3.937E-04 | 3.189E-02 | -4.629072995 |
| hsa-miR-548u | 3.981E-04 | 3.225E-02 | 3.583488081 |
| hsa-miR-3121 | 3.995E-04 | 3.236E-02 | -5.082388082 |
| hsa-miR-1298 | 4.271E-04 | 3.460E-02 | 1.32190084 |
| hsa-miR-2116 | 4.372E-04 | 3.541E-02 | -2.915974195 |
| hsa-miR-412 | 4.708E-04 | 3.813E-02 | -1.360389319 |
| hsa-miR-2355 | 4.731E-04 | 3.832E-02 | 1.836818447 |
| hsa-miR-1299 | 4.992E-04 | 4.043E-02 | 1.767064732 |
| hsa-miR-4642 | 5.022E-04 | 4.068E-02 | -1.548417265 |
| hsa-miR-548t | 5.048E-04 | 4.089E-02 | 3.914741494 |
| hsa-miR-548al | 5.157E-04 | 4.177E-02 | 1.570862046 |
| hsa-miR-1276 | 5.163E-04 | 4.182E-02 | -1.998037009 |
| hsa-miR-4728 | 5.272E-04 | 4.270E-02 | -1.48195609 |
