# Supplementary material for: Identification of Crucial lncRNAs, miRNAs, mRNAs, and Potential Therapeutic Compounds for Polycystic Ovary Syndrome by Bioinformatics Analysis
Source: Biomed Res Int. 2020 Nov 6;2020:1817094. doi: 10.1155/2020/1817094 (PMC7666708; doi:10.1155/2020/1817094)
Supplement: Supplementary 3 — Table S3: all differential miRNAs in GSE84376 dataset. [file 1817094.f3.docx]

**Table S3 Differentially expressed miRNAs in GSE84376**

| miRNA | FDR | P-value | logFC |
| --- | --- | --- | --- |
| hsa-miR-4286 | 1.76E-05 | 1.43E-03 | -1.04 |
| hsa-miR-378c | 1.65E-04 | 1.34E-02 | -0.931 |
| hsa-miR-422a | 6.91E-05 | 5.60E-03 | -0.856 |
| hsa-miR-378f | 7.49E-05 | 6.07E-03 | -0.85 |
| hsa-miR-432 | 2.13E-04 | 1.73E-02 | -0.827 |
| hsa-miR-720 | 2.49E-04 | 2.02E-02 | -0.821 |
| hsa-miR-370 | 8.38E-08 | 6.79E-06 | -0.814 |
| hsa-miR-21 | 1.91E-04 | 1.55E-02 | -0.806 |
| hsa-miR-379 | 2.63E-04 | 2.13E-02 | -0.805 |
| hsa-miR-4485 | 1.84E-07 | 1.49E-05 | -0.782 |
| hsa-miR-382 | 2.91E-07 | 2.36E-05 | -0.76 |
| hsa-miR-378i | 9.53E-05 | 7.72E-03 | -0.705 |
| hsa-miR-17 | 2.04E-04 | 1.65E-02 | -0.64 |
| hsa-miR-378 | 2.66E-04 | 2.16E-02 | -0.64 |
| hsa-miR-320e | 1.26E-04 | 1.02E-02 | -0.605 |
| hsa-miR-34a | 3.19E-04 | 2.58E-02 | -0.592 |
| hsa-miR-30e | 2.11E-04 | 1.71E-02 | -0.588 |
| hsa-miR-4443 | 1.64E-04 | 1.33E-02 | -0.584 |
| hsa-miR-223 | 4.98E-04 | 4.04E-02 | -0.572 |
| hsa-miR-146b | 4.12E-04 | 3.34E-02 | -0.56 |
| hsa-miR-1260b | 3.41E-04 | 2.76E-02 | -0.533 |
| hsa-miR-19b-1 | 1.67E-05 | 1.35E-03 | -0.526 |
| hsa-miR-345 | 2.10E-07 | 1.70E-05 | -0.507 |
| hsa-miR-134 | 4.96E-07 | 4.02E-05 | -0.503 |
| hsa-miR-4284 | 3.46E-04 | 2.80E-02 | -0.503 |
| hsa-miR-376c | 1.94E-07 | 1.57E-05 | -0.466 |
| hsa-miR-409 | 3.77E-07 | 3.05E-05 | -0.466 |
| hsa-miR-197 | 4.13E-07 | 3.35E-05 | -0.439 |
| hsa-miR-494 | 3.99E-07 | 3.23E-05 | -0.424 |
| hsa-miR-487b | 7.22E-07 | 5.85E-05 | -0.344 |
| hsa-miR-487a | 5.65E-07 | 4.57E-05 | -0.335 |
| hsa-miR-381 | 1.75E-07 | 1.42E-05 | -0.246 |
| hsa-miR-195 | 1.78E-07 | 1.44E-05 | 0.27 |
| hsa-miR-4767 | 1.20E-07 | 9.71E-06 | 0.271 |
| hsa-miR-574 | 3.72E-07 | 3.01E-05 | 0.303 |
| hsa-miR-510 | 6.82E-07 | 5.52E-05 | 0.317 |
| hsa-miR-885 | 7.53E-08 | 6.10E-06 | 0.367 |
| hsa-miR-4687 | 1.05E-07 | 8.48E-06 | 0.393 |
| hsa-miR-106b | 2.02E-07 | 1.63E-05 | 0.417 |
| hsa-miR-200c | 7.13E-07 | 5.78E-05 | 0.48 |
| hsa-miR-149 | 8.11E-08 | 6.57E-06 | 0.494 |
| hsa-miR-4763 | 8.59E-05 | 6.96E-03 | 0.502 |
| hsa-miR-1228 | 1.47E-04 | 1.19E-02 | 0.506 |
| hsa-miR-4734 | 1.33E-04 | 1.08E-02 | 0.522 |
| hsa-miR-1910 | 1.88E-04 | 1.52E-02 | 0.522 |
| hsa-miR-542 | 3.16E-04 | 2.56E-02 | 0.525 |
| hsa-miR-3180-4 | 3.42E-04 | 2.77E-02 | 0.533 |
| hsa-miR-4298 | 3.91E-04 | 3.17E-02 | 0.535 |
| hsa-miR-508 | 3.95E-07 | 3.20E-05 | 0.548 |
| hsa-miR-3656 | 3.10E-05 | 2.51E-03 | 0.549 |
| hsa-miR-3648 | 2.61E-05 | 2.11E-03 | 0.555 |
| hsa-miR-3141 | 7.67E-05 | 6.21E-03 | 0.558 |
| hsa-miR-4516 | 1.92E-05 | 1.56E-03 | 0.559 |
| hsa-miR-4463 | 3.30E-05 | 2.67E-03 | 0.559 |
| hsa-miR-182 | 3.78E-07 | 3.06E-05 | 0.56 |
| hsa-miR-4270 | 1.47E-04 | 1.19E-02 | 0.56 |
| hsa-miR-3196 | 4.25E-05 | 3.44E-03 | 0.578 |
| hsa-miR-4690 | 4.01E-05 | 3.25E-03 | 0.595 |
| hsa-miR-3940 | 3.94E-05 | 3.19E-03 | 0.597 |
| hsa-miR-4281 | 5.44E-05 | 4.41E-03 | 0.597 |
| hsa-miR-4745 | 8.10E-05 | 6.56E-03 | 0.613 |
| hsa-miR-3197 | 2.49E-04 | 2.01E-02 | 0.618 |
| hsa-miR-4484 | 2.65E-07 | 2.15E-05 | 0.631 |
| hsa-miR-4505 | 1.51E-04 | 1.22E-02 | 0.635 |
| hsa-miR-3937 | 9.80E-05 | 7.93E-03 | 0.644 |
| hsa-miR-4674 | 2.40E-04 | 1.95E-02 | 0.648 |
| hsa-miR-4665 | 4.91E-05 | 3.98E-03 | 0.668 |
| hsa-miR-4532 | 2.07E-04 | 1.68E-02 | 0.693 |
| hsa-miR-514b | 2.44E-04 | 1.97E-02 | 0.699 |
| hsa-miR-3180-1 | 2.41E-04 | 1.95E-02 | 0.701 |
| hsa-miR-4651 | 6.10E-05 | 4.94E-03 | 0.704 |
| hsa-miR-509-1 | 2.78E-04 | 2.25E-02 | 0.707 |
| hsa-miR-3185 | 8.52E-05 | 6.90E-03 | 0.716 |
| hsa-miR-1587 | 8.79E-06 | 7.12E-04 | 0.738 |
| hsa-miR-4492 | 2.76E-04 | 2.24E-02 | 0.738 |
| hsa-miR-4467 | 2.90E-04 | 2.35E-02 | 0.739 |
| hsa-miR-4707 | 3.91E-08 | 3.17E-06 | 0.761 |
| hsa-miR-513a-1 | 2.60E-04 | 2.11E-02 | 0.768 |
| hsa-miR-1225 | 1.12E-05 | 9.05E-04 | 0.788 |
| hsa-miR-4486 | 1.32E-04 | 1.07E-02 | 0.813 |
| hsa-miR-509-3 | 7.40E-05 | 5.99E-03 | 0.833 |
| hsa-miR-3621 | 1.14E-04 | 9.22E-03 | 0.883 |
| hsa-miR-4689 | 4.82E-05 | 3.90E-03 | 0.887 |
| hsa-miR-4741 | 5.08E-05 | 4.11E-03 | 0.888 |
| hsa-miR-126 | 3.65E-07 | 2.96E-05 | 0.895 |
| hsa-miR-4507 | 4.47E-05 | 3.62E-03 | 0.915 |
| hsa-miR-4695 | 7.42E-05 | 6.01E-03 | 0.915 |
| hsa-miR-4449 | 1.39E-04 | 1.13E-02 | 0.92 |
| hsa-miR-3162 | 3.44E-05 | 2.79E-03 | 0.967 |
| hsa-miR-455 | 5.73E-05 | 4.64E-03 | 1 |
| hsa-miR-3609 | 3.94E-05 | 3.19E-03 | 1.04 |
| hsa-miR-486 | 6.00E-08 | 4.86E-06 | 1.05 |
| hsa-miR-4749 | 1.45E-05 | 1.17E-03 | 1.05 |
| hsa-miR-92b | 7.50E-05 | 6.07E-03 | 1.08 |
| hsa-miR-1246 | 1.80E-04 | 1.46E-02 | 1.11 |
| hsa-miR-4417 | 1.44E-05 | 1.17E-03 | 1.12 |
| hsa-miR-4649 | 3.90E-05 | 3.16E-03 | 1.12 |
| hsa-miR-4758 | 4.67E-05 | 3.79E-03 | 1.12 |
| hsa-miR-3687 | 6.38E-05 | 5.16E-03 | 1.13 |
| hsa-miR-4750 | 1.96E-08 | 1.59E-06 | 1.17 |
| hsa-miR-3135b | 7.78E-06 | 6.31E-04 | 1.18 |
| hsa-miR-1231 | 3.90E-05 | 3.16E-03 | 1.21 |
| hsa-miR-4433 | 3.04E-06 | 2.46E-04 | 1.23 |
| hsa-miR-1909 | 1.63E-08 | 1.32E-06 | 1.24 |
| hsa-miR-451 | 1.16E-04 | 9.40E-03 | 1.41 |
| hsa-miR-3188 | 2.61E-06 | 2.12E-04 | 1.48 |
